# Novel minimal physiologically-based model for the prediction of passive tubular reabsorption and renal excretion clearance

**DOI:** 10.1016/j.ejps.2016.03.018

**Published:** 2016-10-30

**Authors:** Daniel Scotcher, Christopher Jones, Amin Rostami-Hodjegan, Aleksandra Galetin

**Affiliations:** a Centre for Applied Pharmacokinetic Research, Manchester Pharmacy School, University of Manchester, Manchester, United Kingdom; b Oncology iMed, AstraZeneca, Alderley Park, United Kingdom; c Simcyp Limited (a Certara Company), Sheffield, United Kingdom

**Keywords:** AUC, area under the plasma concentration-time profile, BCRP, breast cancer resistance protein, CD, collecting duct, C_p_, plasma concentration, CL_R_, renal excretion clearance, CL_R, filt_, renal filtration clearance, CL_R, int,reab_, intrinsic permeability clearance in renal tubule, CL_R,sec_, renal secretion clearance, DT, distal tubule, f_u,p_, fraction of drug unbound in plasma, F_reab_, fraction of the drug reabsorbed in the renal tubule, F_reab_’, intermediate model parameter, representing the fraction of the equilibrium reached between unbound drug concentration in the plasma and urine, GFR, glomerular filtration rate, gmfe, geometric mean fold error, IVIVE, *in vitro–in vivo* extrapolation, LogD, octanol-buffer distribution coefficient, LoH, loop of Henle, MATE, multidrug and toxin extrusion protein, MRP, multidrug resistance protein, OAT, organic anion transporter, OCT, organic cation transporter, OATP, organic anion-transporting peptides, OCTN, organic cation/l-carnitine transporter, P_app_, apparent permeability, PBPK, physiologically-based pharmacokinetic, P-gp, P-glycoprotein, PT, proximal tubule, RMSE, root mean squared error, TFR, tubular flow rate, TSA, tubular surface area, *In vitro*–*in vivo* extrapolation, Tubular reabsorption, Renal excretion clearance

## Abstract

**Purpose:**

Develop a minimal mechanistic model based on *in vitro–in vivo* extrapolation (IVIVE) principles to predict extent of passive tubular reabsorption. Assess the ability of the model developed to predict extent of passive tubular reabsorption (F_reab_) and renal excretion clearance (CL_R_) from *in vitro* permeability data and tubular physiological parameters.

**Methods:**

Model system parameters were informed by physiological data collated following extensive literature analysis. A database of clinical CL_R_ was collated for 157 drugs. A subset of 45 drugs was selected for model validation; for those, Caco-2 permeability (P_app_) data were measured under pH 6.5–7.4 gradient conditions and used to predict F_reab_ and subsequently CL_R_. An empirical calibration approach was proposed to account for the effect of inter-assay/laboratory variation in P_app_ on the IVIVE of F_reab_.

**Results:**

The 5-compartmental model accounted for regional differences in tubular surface area and flow rates and successfully predicted the extent of tubular reabsorption of 45 drugs for which filtration and reabsorption were contributing to renal excretion. Subsequently, predicted CL_R_ was within 3-fold of the observed values for 87% of drugs in this dataset, with an overall gmfe of 1.96. Consideration of the empirical calibration method improved overall prediction of CL_R_ (gmfe = 1.73 for 34 drugs in the internal validation dataset), in particular for basic drugs and drugs with low extent of tubular reabsorption.

**Conclusions:**

The novel 5-compartment model represents an important addition to the IVIVE toolbox for physiologically-based prediction of renal tubular reabsorption and CL_R_. Physiological basis of the model proposed allows its application in future mechanistic kidney models in preclinical species and human.

## Graphical abstract

**Figure:**
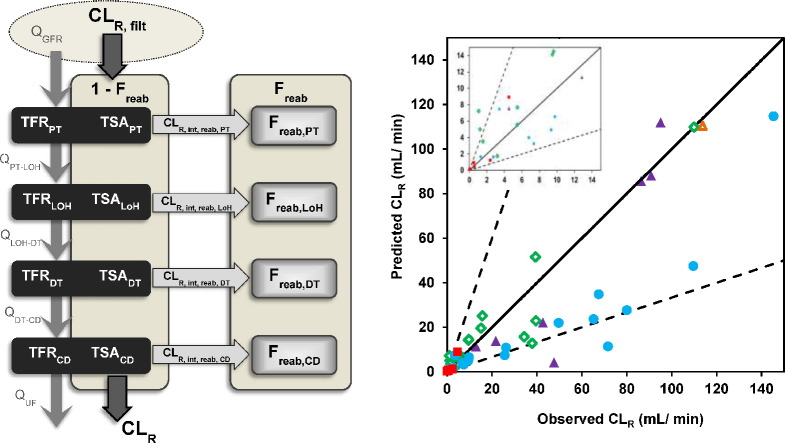

## 1 Introduction

Renal excretion is considered a major route of elimination for many drugs (*e.g.*, metformin, acyclovir and digoxin) (Morrissey et al., 2013, Tucker, 1981, Varma et al., 2009). Prediction of human renal excretion clearance (CL_R_) prior to commencing first-in-man clinical studies currently relies on *in silico* methods based on physico-chemical properties (Dave and Morris, 2015a, Ito et al., 2013, Paine et al., 2010, Varma et al., 2009) and/or allometric scaling (Huh et al., 2011, Paine et al., 2011). Despite wide use of these methods, they do not provide mechanistic insight into the underlying processes contributing to renal excretion and have limited ability to account for any changes in the renal physiology. Mechanistic understanding of various pharmacokinetic (PK) processes has become a necessary part of model-informed decision making for special populations (*e.g.*, obese or patients with renal impairment), as well as devising dosage regimens for use in such populations (Jadhav et al., 2015). The mechanistic approach becomes even more important when certain sub-groups (‘complex’ patients) exhibit various co-morbidities which make clinical studies very difficult, if not impossible (Rostami-Hodjegan, 2015). Thus, understanding various elements of renal excretion may offer advantages through prediction of potential differences in various patients under the framework of physiologically-based pharmacokinetic (PBPK) modelling (Zhao et al., 2011). In addition, many currently developed drugs undergo extensive active tubular secretion (Morrissey et al., 2013) for which prediction of CL_R_ by mechanistic PBPK models (Felmlee et al., 2013, Neuhoff et al., 2013, Posada et al., 2015) is considered more promising in comparison with *in silico* and allometric scaling.

While efforts have been made at predicting renal metabolic clearance from *in vitro* data (Gill et al., 2012, Gill et al., 2013), successful prediction of CL_R_ using *in vitro–in vivo* extrapolation (IVIVE) remains a challenge. In order to quantitatively and mechanistically predict CL_R_ using IVIVE, each of the contributing processes (glomerular filtration, active secretion and tubular reabsorption, Eq. (1)) must be considered independently.

(1) $${\mathrm{CL}}_{R}=\left({\mathrm{CL}}_{R,\text{filt}}+{\mathrm{CL}}_{R,\sec}\right)\times \left(1-F_{\text{reab}}\right)$$

Filtration clearance (CL_R,filt_) is readily predicted from glomerular filtration rate (GFR) and fraction unbound in plasma (f_u,p_). In cases where both secretion and reabsorption contribute to elimination, confidence in prediction of the fraction reabsorbed (F_reab_) is equally important as the accurate prediction of renal secretion clearance (CL_R,sec_). Whereas reabsorption is predominantly a passive process, secretion is actively mediated by a range of drug transporters expressed in the kidney such as OAT1, OAT3, OCT2 and MATE2-K (Morrissey et al., 2013).

A number of mathematical models concerning physiological functions of the kidney (*e.g.*, urine concentrating mechanism, solute transport regulation) exist (Layton, 2011, Weinstein, 2015), but may not be readily adaptable for use in renal PBPK models. Further, these models were developed based on physiological and experimental data in rat kidney (*e.g.*, from micropuncture studies) for which analogous data in human are lacking. Recently, a static model for the prediction of CL_R_ using *in vitro* permeability data from LLC-PK_1_ cell monolayers was proposed and its performance was assessed against a relatively small and restricted dataset (Kunze et al., 2014). The model considered both active secretion and tubular reabsorption, and used the proximal tubule surface area as the IVIVE scaling factor for the apparent permeability (P_app_) data. However, the remaining tubular regions (*e.g.*, collecting duct), which may contribute to passive tubular reabsorption, were not considered (Kunze et al., 2014).

A dynamic kidney model that facilitates IVIVE of renal transporter kinetics and passive permeability has recently been reported (Neuhoff et al., 2013). Although very promising, paucity of data on relevant physiological scaling factors and some of the system data (*e.g.*, transporter abundance) limit model application and validation. In addition, adequate consideration of the heterogeneity of the renal tubule, important for prediction of passive permeability clearance in each tubular segment, is lacking. Current reports on the use of physiologically-based kidney models for ‘bottom-up’ prediction of renal drug disposition often rely on clinical plasma and/or urine drug concentration data for derivation/optimisation of transporter kinetic parameters and their scaling factors (Dave and Morris, 2015b, Felmlee et al., 2013, Hsu et al., 2014, Watanabe et al., 2011), analogous to the trends seen with prediction of hepatic clearance (Galetin, 2014, Zamek-Gliszczynski et al., 2013). For example, IVIVE of human CL_R,sec_ from *in vitro* uptake data obtained in precision cut kidney slices required an empirical scaling factor of 10 in order to obtain agreement between predicted and observed values (Watanabe et al., 2011). In an analogous manner OAT3 maximal uptake rate (V_max_) was optimised using plasma concentration–time profiles to refine prediction of pemetrexed CL_R_ using a PBPK kidney model, and account for differences in transporter expression and activity between the *in vitro* transfected cell system and *in vivo* (Posada et al., 2015).

The aim of this study was to develop a mechanistic model to predict extent of passive tubular reabsorption from *in vitro* permeability data and tubular physiological parameters. The second aim was to assess the ability of the model developed to predict CL_R_ for a range of drugs for which filtration or reabsorption appeared to be the dominant mechanisms contributing to CL_R_. The physiological aspects of the model were informed from the data collated following an extensive literature analysis. A database of *in vivo* CL_R_ and corresponding F_reab_ was collated for 157 drugs. For a subset of 45 selected drugs, *in vitro* permeability data were generated in Caco-2 cell monolayer under pH 6.5–7.4 gradient conditions. Subsequently, the tubular reabsorption model developed was applied to predict regional and overall passive tubular reabsorption for the selected drug subset (n = 45). An empirical calibration approach was proposed to account for the effect of inter-assay/laboratory variation in P_app_ on the IVIVE of F_reab_ using a set of reference drugs as calibrators (n = 11). The novel mechanistic 5-compartment model developed enables prediction of the contribution of passive tubular reabsorption to CL_R_ in a physiologically-based manner and is seen as an integral part of complex kidney models.

## 2 Materials and methods

### 2.1 Clinical data collation

CL_R_ data were collated from literature sources and, wherever possible, data were acquired from primary studies. Further data were gathered from review papers where sufficient details on the trial design had been reported. In addition, data from unpublished clinical studies available at https://www.clinicaltrials.gov were also included in the analysis. Where CL_R_ values were not reported in the study, Eqs. (2), (3) were used to calculate CL_R_ from published urinary excretion and plasma concentration data. Reports of a drug not being detected unchanged in urine, or having “negligible” CL_R_, were not considered for collation. Data available in graphical format were digitised using GetData Graph Digitizer v2.25 (http://getdata-graph-digitizer.com/).

(2) $${\mathrm{CL}}_{R}=\frac{{\text{Amount excreted in urine}\;}_{0-t}}{{\mathrm{AUC}}_{0-t}}$$

(3) $${\mathrm{CL}}_{R}=\frac{\text{Urinary excretion rate}}{C_{p,\text{midpoint}}}$$

where AUC_0-t_ represents the area under the plasma concentration–time profile, and C_p,midpoint_ represents the plasma concentration at the midpoint of the urinary collection interval from which the urinary excretion rate was measured.

Only CL_R_ data acquired following administration of a drug to healthy adult subjects were included in the database. Data from diseased, obese, elderly or alcoholic subjects were excluded, but exclusion criteria based on sex or ethnicity were not applied. Data acquired after co-administration of multiple drugs (*e.g.*, from drug–drug interaction studies) were generally excluded. An exception was made for trimethoprim and sulfamethoxazole because these drugs are generally co-administered and there is a paucity of data following single drug administration. These studies were considered acceptable as there have been no reports in the literature of interactions at the level of renal excretion between sulfamethoxazole–trimethoprim. Aminoglycosides (amikacin, gentamicin, isepamicin, netilmicin, sisomicin and tobramycin) were excluded. These drugs are reported to accumulate in proximal tubule cells, possibly due to endocytotic luminal uptake mediated by the megalin receptor, causing nephrotoxicity (Moestrup et al., 1995, Nagai and Takano, 2004, Schmitz et al., 2002). Drugs with enantiomer specific renal excretion were excluded, an example being cetirizine (Strolin et al., 2008).

In contrast to previous databases (Varma et al., 2009), CL_R_ data in this database are reported as absolute values, *i.e.* without normalisation for body weight or body surface area. Normalisation was not considered as the majority of literature studies (> 75%) reported absolute CL_R_ values and substantial portion of studies did not report either body weight or body surface area of subjects. In addition, recent publications favour the use of absolute values of markers like GFR and creatinine clearance for the drug dosing recommendation, in contrast to body surface area normalised values (Chew-Harris et al., 2015, Dooley and Poole, 2000, Jones, 2011, Pai, 2010). In cases where CL_R_ data were reported following normalisation to body weight (*e.g.,* mL/min/kg or mL/min/70 kg), CL_R_ data were corrected according to the mean weight or midpoint of the range reported in the study. The CL_R_ data were corrected using an assumed body weight of 70 kg for those studies in which body weights had not been reported, which could have introduced some bias. An analogous approach was applied for CL_R_ data reported following normalisation to body surface area, using a standard value of 1.73 m^2^ for studies where subject body surface area data was not reported.

When clearance values for the same drug were available from multiple sources, anomalies in studies/trials reported for individual drugs were initially identified using the I^2^ statistic for data heterogeneity (Eqs. (4), (5)) (Higgins and Thompson, 2002, Higgins et al., 2003). High heterogeneity was observed for 20 drugs (I^2^ greater than 0.5), of which 8 had very high heterogeneity (I^2^ greater than 0.75). The presence and subsequent exclusion of anomalous studies/trials was identified for drugs with I^2^ greater than 0.5 through visual analyses of study/trial mean and standard deviation data.

(4) $$I^{2}=100\%\times \frac{Q-\mathrm{df}}{Q}$$

where Q is Cochran's heterogeneity statistic, and df is the degrees of freedom

(5) $$Q=\sum \frac{{\left(y_{i}-\frac{\sum \frac{y_{i}}{{\sigma^{2}}_{i}}}{\sum \frac{1}{{\sigma^{2}}_{i}}}\right)}^{2}}{{\sigma^{2}}_{i}}$$

where y_i_ is the mean CL_R_ reported by study i, and σ^2^_i_ is the variance in CL_R_ reported by study i.

In addition to CL_R,_ any measured or estimated creatinine clearances and GFR that had been reported in the same clinical studies were collated where available. These data were corrected for any normalisations that had been applied, as performed for CL_R_. Data reported on f_u,p_ were also collated from the same study. Further f_u,p_ data were obtained using other literature sources. Where nonlinear plasma protein binding was reported for a drug, the f_u,p_ at concentrations consistent with plasma concentrations reported for CL_R_ clinical studies were used for the analysis.

The f_u,p_ data were collated from a variety of *in vivo* and *in vitro* sources using a number of different experimental techniques (ultrafiltration or membrane dialysis). Therefore, the average f_u,p_ value for each drug was obtained without applying a weighting, whilst ensuring good agreement was achieved between the data used to obtain the average.

Overall weighted mean CL_R_ and standard deviation were calculated using Eqs. (6), (7), respectively.

(6) $$W\overset{̅}{X}=\frac{\sum_{j=1}^{J}n_{j}∙{\overset{̅}{x}}_{j}}{\sum_{j=1}^{J}n_{j}}$$

where $W\overset{̅}{X}$ is the weighted mean, n_j_ is the number of subjects in the j^th^ study, and $\overset{̅}{x}j$ is the mean of the j^th^ study. Here a “study” is defined as the data associated with a group of subjects being administered a specific dose regime, on a particular occasion, with “n” number of subjects.

(7) $$\sigma =\sqrt{\frac{\left[\sum_{j=1}^{J}\left[\left({\sigma_{j}}^{2}+{{\overset{̅}{x}}_{j}}^{2}\right)n_{j}\right]\right]-\left[\left(\sum_{j=1}^{J}n_{j}\right)∙{W\overset{̅}{X}}^{2}\right]\;}{\sum_{j=1}^{J}n_{j}}}$$

where σ is the overall weighted standard deviation and σ_j_ is the standard deviation of the j^th^ study.

### 2.2 Calculation of observed clearance ratio and F_reab_

The clearance ratio was calculated from clinical data using Eqs. (8), (9), in agreement with reported studies (Giacomini et al., 2010, Ito et al., 2013). Drugs with a clearance ratio greater than 1.5 were considered to undergo net secretion, and were therefore excluded from subsequent analyses and assessment of the model developed for prediction of tubular reabsorption. For the remaining drugs the fraction reabsorbed (F_reab_) was calculated using Eq. (10).

(8) $${\mathrm{CL}}_{R,\text{filt}}=\mathrm{GFR}\times f_{u,p}$$

(9) $$\text{Clearance Ratio}=\frac{{\mathrm{CL}}_{R}}{{\mathrm{CL}}_{R,\text{filt}}}$$

(10) $$F_{\text{reab}}=1-\frac{{\;\mathrm{CL}}_{R}}{{\mathrm{CL}}_{R,\text{filt}}}$$

N.B. In certain instances, calculated F_reab_ was negative based on Eq. (10). For these drugs (6/45 drugs), data in figures were presented as F_reab_ = 0 (actual negative values were used for numerical analyses). The most extreme example was atenolol, with the observed weighted overall CL_R_ of 145 ± 48 mL/min (f_u,p_ of 0.97) which exceeded GFR assumed in the current study.

GFR values for healthy subjects may vary as a result of biological as well as inter-individual variation and also the method of measurement. As the more robust methods for measuring GFR, such as inulin or iohexol renal clearance, are impractical and time consuming (Sterner et al., 2008), GFR measurements during clinical studies are typically based on the renal clearance of endogenous creatinine. This method is associated with a degree of inaccuracy, due to the proposed contribution of active secretion by the OCT2, MATE1 and MATE2-K transporters to creatinine clearance (Soveri et al., 2014, Tanihara et al., 2007, Urakami et al., 2004). More frequently GFR is not measured but estimated using creatinine plasma concentrations using either the ‘Cockcroft–Gault’ or ‘Modification of Diet in Renal Disease’ equations (Nyman et al., 2011). Individual GFR values for subjects in clinical trials databases were generally not available. Therefore, for the purposes of this study, a GFR value of 120 mL/min was assumed for all drugs, consistent with precedent set in the literature and value used in the recently developed physiologically-based kidney model (Delanaye et al., 2012, Neuhoff et al., 2013).

### 2.3 *In vitro* permeability data, physico-chemical properties and drug affinity for renal transporter proteins

In the absence of a robust and validated *in vitro* model for assessing passive permeability in the renal nephron tubule, it was hypothesised that permeability data obtained in Caco-2 cell monolayers may offer a potential substitute. Apparent permeability (P_app_) of drugs was measured in the apical to basolateral direction in Caco-2 cell monolayers using an AstraZeneca in-house assay (Hilgendorf, C. and Fredlund, L., Intrinsic permeability *in-vitro* — a transporter independent measure of Caco-2 permeability in drug design and development; poster presented at World Conference on Drug Absorption, Transport and Delivery (WCDATD): responding to challenging situations; Uppsala, Sweden June 24–26, 2013). The assay was performed using the apical to basolateral pH gradient format of pH 6.5 to pH 7.4. Permeability assays were performed in the presence of an efflux transporter inhibitor cocktail (50 μM quinidine, 20 μM sulfasalazine, 100 μM benzbromarone) over a 2 h incubation period. The pH gradient was applied in order to mimic typical conditions observed in the renal tubule, where the urine pH can vary between 4.5 and 8, but is typically more acidic relative to plasma (pH 5.5–6.5) (Simerville et al., 2005). The efflux transporter inhibitor cocktail was used to minimise the effect of efflux transporters expressed in Caco-2 cells (*e.g.* P-glycoprotein, BCRP and MRP2) on the estimate of apical to basolateral P_app_ of substrates for such transporters. P_app_ data were obtained for a subset of drugs that exhibited a variety of physico-chemical properties, range of CL_R_, F_reab_ and expected P_app_ values (based on historical AstraZeneca in-house data and literature analysis of permeability data generated under isotonic pH 7.4 conditions).

The octanol-buffer (pH 7.4) distribution coefficient (LogD_7.4_) and pK_a_ data were generated using slight modification of the shake-flask (Wenlock et al., 2011) and potentiometric AstraZeneca in-house assays respectively. Where measured LogD_7.4_ or pK_a_ data could not be obtained, values reported in the BioByte Masterfile database (BioByte Corporation, Claremont, CA, USA, http://www.biobyte.com/index.html) or calculated using ACD/LogD program v.14.02 (Advanced Chemistry Development, Inc., Toronto, On, Canada, www.acdlabs.com, 2014) were used. The pK_a_ values associated with ionisable centres identified within a given drug were used to calculate the fraction of drug ionised at pH 6.5. Drugs were then classified into groups as follows: drugs with > 50% unionised at pH 6.5 were classified as “Neutral”; drugs with > 50% ionised as mono −/di −/tri-protic acids or bases were classified as “Acid” or “Base”, respectively; “Zwitterions” were classified as drugs with > 50% ionised, with the major ionised species having no net charge; all other drugs were classified as amphoteric. The LogD_6.5_ of acids and bases were estimated from LogD_7.4_ and pK_a_ data assuming that only the neutral species partitions into the octanol phase (Kah and Brown, 2008). LogD_6.5_ for neutral, amphoteric and zwitterion drugs were assumed to be equal to LogD_7.4_. Although melagatran, oxytetracycline and tetracycline have an isoelectric point > 7.4 or < 6.5, the difference between LogD_7.4_and LogD_6.5_ predicted by ACD was less than 0.5, confirming the validity of the assumption above.

The potential impact of renal transporter mediated secretion of drugs on the assessment of the prediction of CL_R_ using the minimal model of reabsorption was investigated. A thorough literature search was performed in PubMed (http://www.ncbi.nlm.nih.gov/pubmed) to identify drugs reported to be substrates of OAT1, OAT3, OAT4, OATP4C1, OCT2, OCTN1, OCTN2, MATE1, MATE2K, P-gp, MRP2, MRP4 or BCRP (Morrissey et al., 2013). To expand this dataset, the UCSF-FDA Transportal and TP-search databases were also searched (Morrissey et al., 2012, Ozawa et al., 2004). Only reports of drugs interacting as substrates of human drug transporters were included. In addition, clinical data indicating occurrence of renal transporter mediated drug–drug interactions (*i.e.*, decrease in CL_R_ following co-administration of another drug) were used as indirect evidence of drugs being substrates of renal drug transporters.

### 2.4 Overall structure of the minimal model of tubular reabsorption

**Fig. 1:**
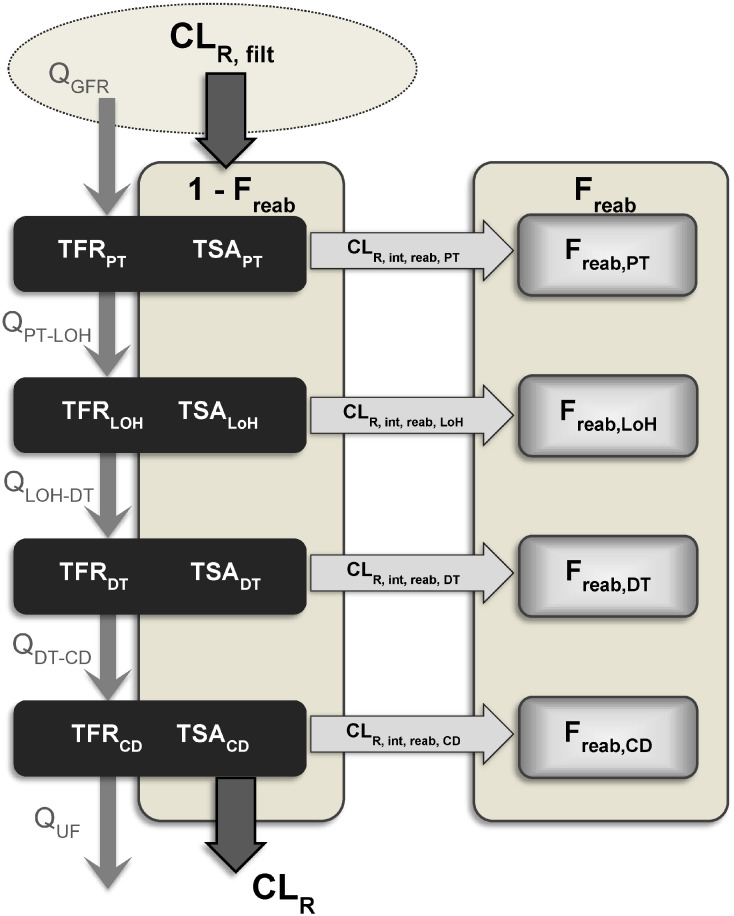
Schematic diagram of the minimal physiologically-based model for tubular reabsorption of drugs in the kidney. The nephron is represented by a glomerulus compartment in addition to four compartments representing different regions of the nephron tubule (proximal tubule (PT), loop of Henle (LoH), distal tubule (DT) and collecting duct (CD) in descending anatomical order). Physiological parameters, tubular flow rate (TFR_i_) and tubular surface area (TSA_i_), for each individual tubular region are indicated. Tubular filtrate flow (Q) is represented by grey arrows connecting tubular compartments and used to calculate TFR_i_ (average midpoint flow rates). The intrinsic reabsorption clearance of each individual region (CL_R,int,reab,i_) is calculated using the corresponding TSA_i_. Total renal excretion clearance (CL_R_) is obtained from the filtration clearance (CL_R,filt_) and the overall fraction reabsorbed (F_reab_), by rearrangement of Eq. (9).

In the proposed model, the nephron is represented as five compartments, namely the glomerulus and four tubular regions, as depicted in Fig. 1. The tubular compartments in the model, listed in anatomical order starting from the glomerulus are: the proximal tubule (PT), the loop of Henle (LoH), the distal tubule (DT) and the collecting duct (CD). In the absence of active processes, tubular reabsorption proceeds until the urinary concentration is in equilibrium with the unbound drug plasma concentration. Thus a F_reab_ value of 1, and CL_R_ of 0, is not strictly possible in this case. The minimal model of reabsorption was developed by introducing an intermediary parameter, F_reab_′, the value of which could range from 0 to 1, representing the fraction of the equilibrium reached between urine and plasma. The relationship between F_reab_′ and F_reab_ is shown in Eq. (11). The predicted overall F_reab_′ was calculated from the predicted F_reab_′ in each (i) tubular region (F_reab,i_′), as shown in Eq. (12). Regional F_reab,i_′ (Eq. (13)) were predicted from the intrinsic permeability clearance of the drug of interest for each tubule region (CL_R,int,reab,i_) and the tubular flow rate (TFR) of the filtrate/urine in the corresponding region (TFR_i_), approach adapted from previous studies (Kunze et al., 2014).

(11) $${F_{\text{reab}}}^{'}=F_{\text{reab}}\times \frac{{\mathrm{CL}}_{R,\text{filt}}}{{\mathrm{CL}}_{R,\text{filt}}-\left(\mathrm{UF}\times f_{u,p}\right)\;}$$

where UF is the urine flow, which was assumed to be 1 mL/min

(12) Freab'=1-∏1-Freab,i'

(13) $$F_{\text{reab},i}^{'}=\frac{{\mathrm{CL}}_{R,\mathrm{int},\text{reab},i}}{{\mathrm{TFR}}_{i}+{\mathrm{CL}}_{R,\mathrm{int},\text{reab},i}}.$$

CL_R,int,reab,i_ for each drug and tubular region combination was calculated using an IVIVE approach, as per Eq. (14) (Kunze et al., 2014).

(14) $${\mathrm{CL}}_{R,\mathrm{int},\text{reab},i}=P_{\mathrm{app}}\times {\mathrm{TSA}}_{i}$$

where TSA_i_ is the tubule surface area for each individual tubular region.

Based on the scope of the model, renal drug metabolism was not considered. In addition, there was no evidence of renal metabolism for the majority of drugs used to assess the predictive performance of the model of reabsorption.

### 2.5 Minimal model of tubular reabsorption: Physiological system parameters

Final values of physiological input parameters for the four tubular compartments are shown in Table 1, with the full detail on the individual parameters (*e.g.,* tubular region diameters and length) presented in the Supplementary Methods. In general, TFR_i_ values were the midpoint flow rates for each tubular compartment (Table S1.1 and S1.2). TSA_i_ values were initially calculated following the assumption of the surface area of a cylinder, using length and diameter of tubular region (Tables S1.3 and S1.4), and the number of nephrons of 900,000 nephrons/kidney. Special consideration was made for the CD compartment, due to the merging of nephrons to form the cortical CD, and merging of CDs in the inner medulla. To account for this, the number of nephrons was reduced to 90,000 nephrons/kidney for the cortical and outer medulla CD, with surface area calculated following assumption of a cylinder. The surface area of the inner medulla CD was calculated using an exponential function shown in Eq. (15) (details provided in Supplementary Methods, Section 2 and Fig. S1.1) which accounts for the concomitant decrease in number and increase in diameter of CD, as they traverse towards the renal pelvis.

(15) $$C_{x}=\left(d_{0}\times {\mathrm{NCD}}_{0}\times \pi \right)e^{\left(\;\left(\frac{x\times F}{n}\right)\times \ln\left(\frac{2}{{\frac{d_{0}}{d_{n}}}^{\frac{1}{F}}}\right)\right)}$$

where d_0_ and d_n_ are the diameter of inner medulla collecting ducts at the papilla apex and at the outer medulla-inner medulla boundary, NCD_0_ is the number of inner medulla CD at the papilla apex, and F is the number of fusion events. C_x_ represents the total circumference of inner medulla CD throughout this region at ‘x’ mm from the papilla apex. The inner medulla CD surface area is the area under the curve of this function between 0 and n, where n is the length of the IMCD (length = inner medulla width = 11 mm).

Microvilli are present extensively in epithelial cells of the proximal tubule where they form a brush border; in contrast, microvilli are sparse in tubular cells of other regions (Welling et al., 1981, Welling and Welling, 1988). Additionally, there is evidence of the expression of microvilli in the Caco-2 cells. Therefore, a microvilli correction factor was applied to TSA_i_ values in the DT, LoH and CD compartments to account for the differential presence of microvilli along the renal tubule relative to the PT region and Caco-2 cells. This approach resulted in a 7.5-fold decrease in the estimated surface area compared to that calculated using the assumption of open cylinder for the LoH, DT and CD compartments. The method used here is analogous to a recently published approach to estimate specific regional effective permeability for intestinal PBPK models used to predict drug absorption (Olivares-Morales et al., 2015).

### 2.6 Empirical relationship between P_app_ and observed F_reab_′

The empirical relationship between P_app_ and F_reab_′ was best described by the Hill model (Eq. (16)), consistent with the relationship already defined between P_app_ across Caco-2 cell monolayers and fraction absorbed following oral administration (Artursson et al., 2001).

(16) Freab'=Pappaba+Pappa

where a represents the slope factor and b is the value of P_app_ at which F_reab_′ equals 0.5. The Hill model was fitted to the data using nonlinear regression to estimate best-fit values and 95% confidence interval (CI) of parameters.

### 2.7 Calibration of Caco-2 permeability data

Permeability data from Caco-2 cell monolayer assays generally show inter-laboratory variation (Artursson et al., 2001). To account for this and to allow the Caco-2 data in the present study to be transferable to other assay formats/laboratories, a P_app–_F_reab_′ calibration method was explored by splitting the available P_app_ and F_reab_′ data into ‘reference’ and internal ‘validation’ subsets. It is important to clarify that this calibration is not intended to predict F_reab_′ using a data driven empirical model, as done in quantitative structure–pharmacokinetic relationship models, which require formal internal and external validation (Dave and Morris, 2015a). The calibration approach is proposed as a pragmatic method to account for inter-assay/inter-laboratory differences; herein it is applied to the current dataset only, and not external data as intended for future studies.

The reference drugs (n = 11) were selected to cover a range of P_app_ and F_reab_′ values and were representative of the overall relationship between P_app_ and F_reab_′. The relationship between P_app_ and the predicted F_reab_′ based on the minimal reabsorption model was best described by the Hill model, analogous to the relationship between P_app_ and observed F_reab_′ discussed above. Empirical calibration was performed using the reference dataset of 11 drugs to account for any discrepancy between predicted and observed F_reab_′. This approach allowed subsequent calculation of calibrated P_app_ values for the drugs in the internal validation dataset (n = 34), using Eq. (17) (see Supplementary Methods for derivation).

(17) $$P_{\mathrm{app},\text{calibrated}}=\frac{b_{1}\times {P_{\mathrm{app}}}^{\left(\frac{a_{2}}{a_{1}}\right)}}{{b_{2}}^{\left(\frac{a_{2}}{a_{1}}\right)}}$$

where a_1_ and b_1_ are the slope factor and P_app_ value at which F_reab_′ is equal to 0.5 for the P_app_ vs F_reab_′ Hill equation fitted against data predicted by the minimal model for the reference dataset; a_2_ and b_2_ are the Hill equation parameters obtained from fitting P_app_ values for the reference drugs (P_app,ref_) and corresponding observed F_reab_′.

### 2.8 Data analysis

Nonlinear regression was carried out using MATLAB R2012a (The MathWorks Inc., Natick, MA, USA, www.mathworks.com). All other data analyses were performed using MS Excel. The model performance was assessed based on the R^2^ of the predicted vs. observed linear regression, and by considering the number and percent of drugs predicted within 3-fold of the observed CL_R_. The performance of models with different physiological complexity was assessed for drugs with low (F_reab_ < 0.25), medium (F_reab_ = 0.25–0.75) and high passive tubular reabsorption (F_reab_ > 0.75). These cut-off values were arbitrarily selected to assess potential differences in trends between these groups of drugs.

Bias and precision in predicting CL_R_ and F_reab_ were calculated as geometric fold error (gmfe) in Eq. (18) and root mean squared error (rmse) in Eq. (19) (Gertz et al., 2010). The gmfe indicates an absolute deviation from the observed data, as this metric does not allow over- and under-predictions to cancel each other out.

(18) $$\text{gmfe}=10^{\frac{1}{n}\sum \left|\log_{10}\left(\frac{\text{Predicted}}{\text{Observed}}\right)\right|}$$

(19) $$\text{RMSE}=\sqrt{\frac{1}{n}\sum {\left(\log\left(\text{Observed}\right)-\log\left(\text{Predicted}\right)\;\right)}^{2}}.$$

## 3 Results

### 3.1 Collation of a comprehensive renal clearance database

CL_R_, f_u,p_ and transporter interaction data were collated for 157 drugs; details are listed in Supplementary Material, Tables S2.1 and S3.1. On average 4–5 clinical studies and 40 CL_R_ measurements were obtained per individual drug, although this varied depending on availability of data. No attempt was made to separate intra- and inter-subject variability. Following analysis using the I^2^ statistic, three studies/trials were classified as anomalous results and excluded; details are presented in the Supplementary Results, Table S4.1. Overall weighted mean CL_R_ ranged from 0.022 (isoxicam) to 526 mL/min (metformin), while f_u,p_ ranged from 0.01 (olmesartan) to 1 (metformin). Measurements and estimates of GFR (n = 1686) were reported from 200 clinical studies (28% of studies collated). Only two of the studies collated used inulin to measure GFR; the remainder used either creatinine clearance, estimated GFR from plasma creatinine concentrations, or did not specify the method. Overall weighted mean GFR was 112.9 ± 25.2 mL/min, with study mean values ranging from 69.2 to 168.0 mL/min.

Of the 157 drugs, 72 were classified as net secreted (clearance ratio > 1.5). As such, they were regarded unsuitable for assessing a model of tubular reabsorption and therefore excluded from further analysis. Glomerular filtration or reabsorption was the dominant mechanism for the remaining 85 drugs (clearance ratio < 1.5). Of these, a representative subset of 45 drugs was selected for the assessment of the predictive performance of the mechanistic tubular reabsorption model. These drugs covered a range of CL_R_ values, from 0.02 (isoxicam) to 145 mL/min (atenolol); the extent of tubular reabsorption ranged from none (six drugs including atenolol and verapamil) to reaching complete equilibrium (F_reab_′ = 1, isoxicam). Drugs were also selected to ensure a range of physico-chemical properties were represented, although neutral and basic drugs represented the majority, with 15 and 16 drugs, out of the set of 45, falling into these categories, respectively (Table 2). Approximately one third of the drugs have been reported to be substrates of human drug transporters known to be expressed in the kidney. Corresponding P_app_ values obtained in the Caco-2 cells under the 6.5–7.4 pH gradient conditions covered approximately 3 orders of magnitude, as shown in Table 2.

Lipophilic drugs, and those unionised at physiological pH, were associated with low CL_R_, (Supplementary Results, Figs. S4.1 and S4.2), in agreement with previous studies (Paine et al., 2010, Varma et al., 2009). No clear trends could be established between LogD_7.4_ or LogD_6.5_ and observed F_reab_ of 45 drugs investigated (data not shown). High LogD_7.4_ did not appear to be predictive of high tubular reabsorption (F_reab_ > 0.75), as a wide range of LogD_7.4_ values were associated with drugs in moderate to low F_reab_ category (Supplementary Results Fig. S4.1). The majority of neutral drugs (62%) had high tubular reabsorption (F_reab_ > 0.75), whereas the majority of ionised drugs had low F_reab_ (< 0.25). The percent of acidic, basic, zwitterion and amphoteric drugs with F_reab_ < 0.25 was 43%, 56%, 61% and 100%, respectively (data presented in detail in the Supplementary Results, Fig. S4.2).

### 3.2 Prediction of CL_R_ from glomerular filtration only

**Fig. 2:**
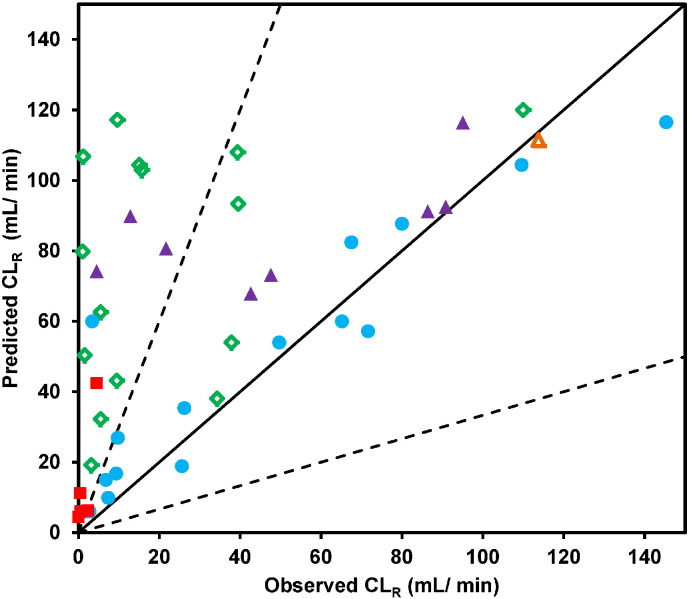
Comparison of predicted and observed CL_R_ using CL_R,filt_ alone to predict CL_R_. Neutral (), basic (), acidic (), zwitterion () and amphoteric () drugs are indicated. Solid and dashed lines represent line of unity and 3-fold error, respectively.

Predictive performance of the tubular reabsorption model was assessed using a subset of 45 drugs. Initial analysis was performed by assessing CL_R_ prediction assuming that the glomerular filtration was the only contributing mechanism (Eq. (8)). This approach resulted in general over-prediction of CL_R_, as shown in Fig. 2 and Table 3; the predicted CL_R_ for each individual drug are listed in the Supplementary Results, Table S4.2. The extent of over-prediction (> 3-fold) was particularly apparent for neutral (10 out of 15 drugs) and acidic drugs (4 out of 5 drugs). Antipyrine (neutral), caffeine (neutral), and isoxicam (acid) were the most pronounced outliers with the extent of over-prediction of CL_R_ ranging from 75- to 202-fold for these drugs. Conversely, the majority of basic drugs were predicted well, especially betaxolol, citalopram, metoprolol and venlafaxine. For these four basic drugs, the assumption of glomerular filtration in isolation resulted in predicted CL_R_ within 10% of the observed values.

### 3.3 Prediction of F_reab_ using the minimal physiologically-based tubular reabsorption model

**Fig. 3:**
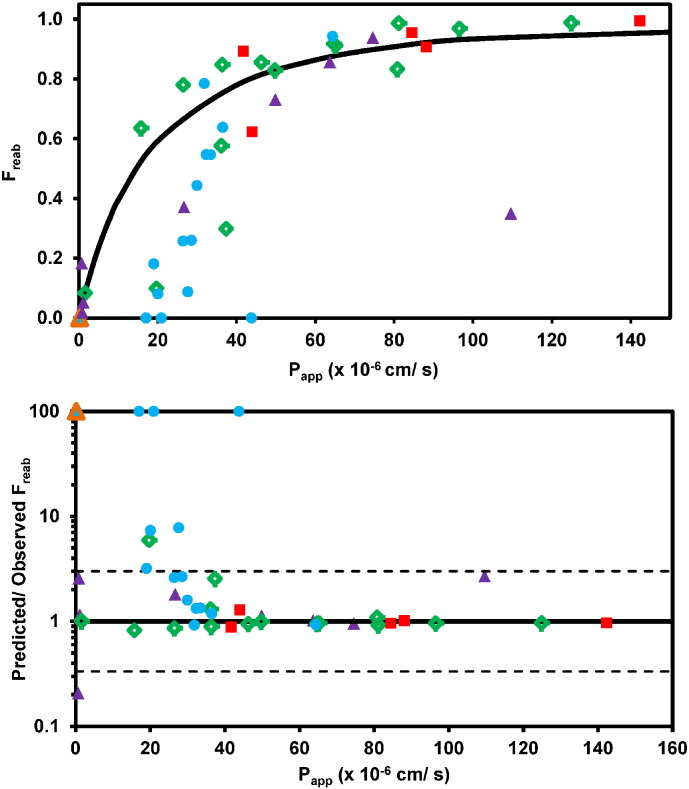
Comparison of observed and predicted F_reab_ using the mechanistic tubular reabsorption model and P_app_ data obtained in Caco-2 cells. Panel A: Predicted relationship between F_reab_ and P_app_ is shown by the solid black line, whereas observed data are shown by symbols; Panel B: Predicted/observed F_reab_ are plotted as symbols, whereas solid and dashed black lines indicate Predicted/observed = 1 and 3-fold error, respectively. Symbols indicate neutral (), basic (), acidic (), zwitterion () and amphoteric () drugs. Drugs with negative values for observed F_reab_ are plotted as F_reab_ = 0 in Panel A, or predicted/observed F_reab_ = 100 in Panel B, as described in the Materials and methods.

The IVIVE approach was used to predict the F_reab_ and CL_R_ from Caco-2 P_app_ data using the minimal mechanistic tubular reabsorption model, as outlined in the Materials and methods (Eqs. (11), (14)). The observed F_reab_ was calculated from reported CL_R_ and f_u,p_ data, assuming the GFR value of 120 mL/min (Fig. 3). In some instances, apparently negative F_reab_ and F_reab_′ values were obtained (*e.g.,* atenolol and verapamil); this is an artefact of the inclusion criteria applied, given that the renal clearance ratio was used as cut off for net secretion (> 1.5). Overall there was a good agreement between predicted and observed F_reab_, albeit with over-prediction of F_reab_ for some drugs with moderate P_app_ values (~ 20–40 × 10^− 6^ cm/s).

**Fig. 4:**
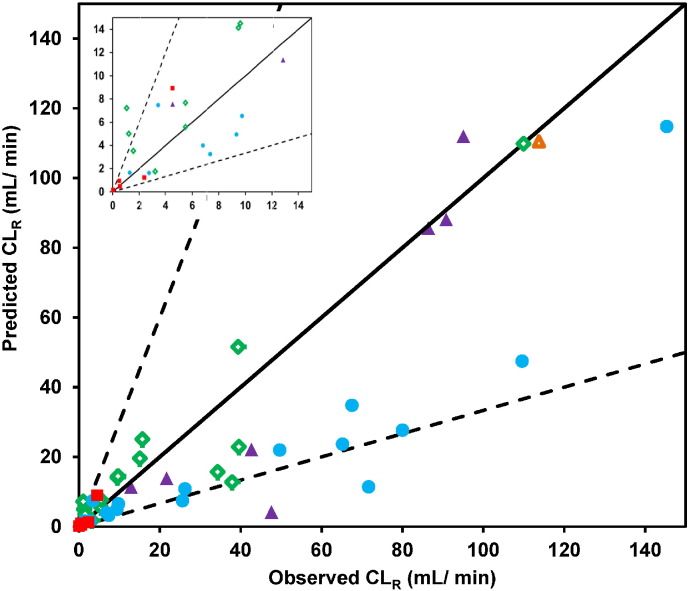
Comparison between observed and predicted CL_R_ by the mechanistic tubular reabsorption model. Symbols indicate neutral (), basic (), acidic (), zwitterion () and amphoteric () drugs respectively. Solid and dashed lines represent line of unity and 3-fold error, respectively. The inset shows the data for lower CL_R_ values for clarity.

The predicted CL_R_ were calculated for 45 drugs using the predicted F_reab_, together with GFR and urine flow (full results listed in Supplementary Results, Table S4.2). There was a good agreement between predicted and observed CL_R_ data with < 2-fold bias for the whole dataset (Fig. 4 and Table 3). In particular, this trend was evident for neutral drugs (gmfe = 1.86), where 87% of CL_R_ were predicted within 3-fold error of the observed value with antipyrine and caffeine being the only exceptions (over-prediction of 4.1 and 6.8-fold, respectively). In the case of basic drugs, a general CL_R_ under-prediction trend was noted, as well as poor precision (RMSE = 31.0), in agreement with the over-prediction of F_reab_ seen for this class of drugs (Fig. 3). Consideration of both glomerular filtration and reabsorption reduced the prediction accuracy in the case of betaxolol, citalopram, metoprolol and venlafaxine. However, the predicted CL_R_ was still within 50–65% of the observed data. Despite this overall CL_R_ under-prediction trend, it is important to note that the values for the majority of basic drugs (14/16) were predicted within 3-fold of the observed. Mexiletine and verapamil were the most pronounced outliers as the predicted CL_R_ represented only 16 and 29% of the observed value, respectively. Consideration of pH gradient and ionisation was of particular relevance for this class of drugs, as the use of P_app_ data obtained under isotonic pH 7.4 conditions resulted in pronounced under-prediction of CL_R_ for half of basic drugs in the dataset, with predicted CL_R_ < 35% of the observed value (Supplementary Results Fig. S4.3).

Excluding drugs which were known/reported substrates of human kidney transporters had negligible impact on the success of CL_R_ prediction (Table 3). In contrast, failing to account for the presence/absence of microvilli expressed by Caco-2 cell monolayers and nephron tubular cells resulted in reduced prediction success with only 27% of drugs predicted within 3-fold of the observed CL_R_. Comparison of predictive performances of different models stratified according to low, medium and highly reabsorption drug status is shown in Table 4.

### 3.4 Empirical relationship between F_reab_′ and P_app_ and calibration approach

**Fig. 5:**
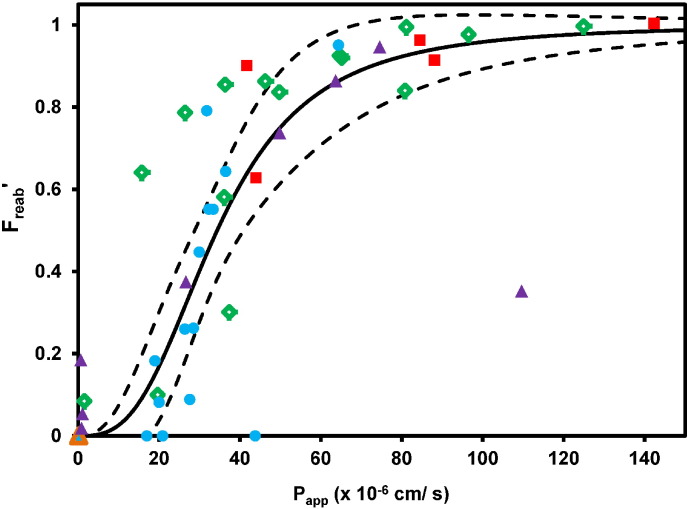
Best-fit curve and 90% confidence interval of the Hill equation to the P_app_ and F_reab_′ data for 45 drugs (solid and dashed lines respectively). Symbols indicate neutral (), basic (), acidic (), zwitterion () and amphoteric () drugs. Drugs with negative values for observed F_reab_′ are plotted as F_reab_′ = 0, as described in the text.

The Hill model was fitted to the observed F_reab_′ and P_app_ data for the 45 drugs selected (Fig. 5). The best-fit value and 95% confidence interval for Caco-2 P_app_ corresponding to F_reab_′ = 0.5 was estimated to be 34.4 (28.9–39.9) × 10^− 6^ cm/s. In addition, the Hill model was fitted to the P_app_ values and F_reab_′ predicted by the mechanistic tubular reabsorption model for 45 drugs. The resultant estimate of P_app_ corresponding to F_reab_′ = 0.5 was 14.8 (14.3–15.2) × 10^− 6^ cm/s.

**Fig. 6:**
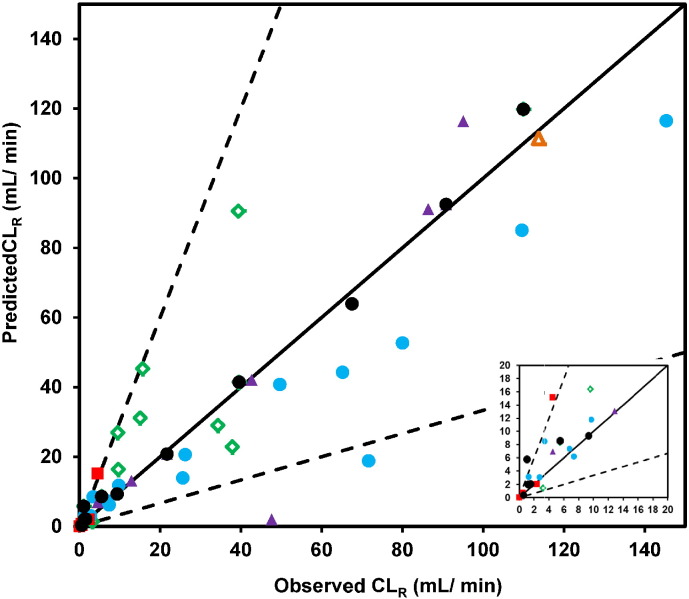
Prediction of CL_R_ using the minimal model following calibration of P_app_ data using reference drugs (n = 45 drugs). Symbols indicate neutral (), basic (), acidic (), zwitterion () and amphoteric () drugs in the internal ‘validation’ dataset and drugs in the reference dataset (●; n = 11). Solid and dashed lines represent line of unity and 3-fold error, respectively. The inset shows the data for lower CL_R_ values for clarity.

In order to evaluate the application of P_app_–F_reab_′ calibration (Eq. (17)), 11 drugs were selected as reference ‘calibrator’ drugs (P_app,ref_) covering a representative range of F_reab_′ and P_app_ values (Table 5). The remaining 34 drugs were treated as an internal ‘validation’ set. Following the fitting of the Hill equation to observed F_reab_′ and P_app,ref_ data for these 11 reference drugs, the best-fit estimates for a_2_ and b_2_ were 2.74 and 33.1 × 10^− 6^ cm/s. These were used to calculate values of P_app,calibrated_ for the validation dataset, and were subsequently applied for prediction of F_reab_′, F_reab_ and CL_R_ using the minimal physiologically-based reabsorption model, as done initially. Use of this calibrated approach led to considerable improvement in the predictive performance with 31/34 drugs in the internal validation set predicted within 3-fold of the observed CL_R_ (gmfe = 1.73, Fig. 6). Comparable success was seen for the full dataset, with 41/45 drugs predicted within 3-fold of the observed CL_R_, and reduced bias (gmfe = 1.65) compared to the model before applying the P_app_ calibration. Particular improvement in the prediction of CL_R_ following the P_app_–F_reab_′ calibration was apparent for basic drugs, as 15/16 drugs were successfully predicted. An increase in prediction accuracy was also observed for neutral drugs (*e.g.*, prednisone) and zwitterions (*e.g.*, moxifloxacin) with moderate P_app_ values (20–40 × 10^− 6^ cm/s). The calibration approach resulted in marginal overall improvement in the prediction of CL_R_ for drugs with F_reab_ > 0.75. However, substantial improvement was noted for 9/11 highly reabsorbed drugs (F_reab_ > 0.9), including isoxicam, probenecid, caffeine and antipyrine.

## 4 Discussion

### 4.1 Physiological considerations for predicting tubular reabsorption

The 5-compartment minimal physiologically-based model was developed for the prediction of tubular reabsorption. Although static, the model accounted for physiological differences between regions of the nephron and captured complex underlying physiology of the kidney in a mechanistic manner. In addition to the model development, a comprehensive database of physiological parameters with relevance to pharmacokinetics of drugs in human kidney was collated. Quantitative human physiological data were sparse in general and available from a few primary research articles, but were supported by data in preclinical species where possible (see Supplementary Methods, Tables S1.1–4). For some physiological parameters, such as glomerular filtration rate, considerable inter-individual variability, as well as bias in some of the commonly used methods has been reported (Delanaye et al., 2012, Soveri et al., 2014, Sterner et al., 2008). The lack of data and information on variability in reported values for physiological parameters will inevitably result in a level of uncertainty associated with the relevant input model parameters. For example, calculation of apparent F_reab_ was dependent on the value of GFR; the sensitivity of F_reab_ to changes in GFR was evident for drugs exhibiting low tubular reabsorption (F_reab_ < 0.25). However, as the reported data on the inter-individual variability in GFR were limited in the clinical studies in the database, its potential impact on the estimation of apparent F_reab_ was not considered in the current analysis.

Predictive performance of the mechanistic tubular reabsorption model was assessed against a representative set of 45 drugs, focusing in particular on the impact of the tubular surface area in the model development (Table 1, Table 4). The mechanistic tubular reabsorption model was applied to predict both regional and overall F_reab_′ for the selected dataset. The analysis has shown that reabsorption in the proximal tubule compartment was the major contributor to the overall predicted F_reab_ for most of the drugs. Consideration of tubular reabsorption solely in this region within the model had marginal impact on the prediction of CL_R_ for drugs with low F_reab_ (*e.g.*, atenolol and melagatran). In contrast, this approach resulted in reduced CL_R_ prediction accuracy compared with the ultimate 5-compartment model for extensively reabsorbed drugs such as antipyrine and isoxicam (Table 4). The reabsorption in other tubular regions was not considered in the previously reported static model (Kunze et al., 2014). However, it is important to note that in the study by Kunze et al. only one drug in the dataset exhibited notable net reabsorption (desipramine, apparent F_reab_ of 0.31) and drugs showing extensive reabsorption (F_reab_′ ~ 1, CL_R_ approaching 1–2 mL/min, (Tucker, 1981)) were not included in the analysis.

The 5-compartment tubular reabsorption model was also able to account for regional differences in the expression of microvilli and subsequent effect on the surface area to be used for scaling of permeability data. A pronounced under-prediction of CL_R_ was observed for most of the drugs in the dataset if differences in microvilli related surface area between Caco-2 monolayers and the loop of Henle, distal tubule and collecting duct regions were ignored (Supplementary Results Fig. S.4.4); the exceptions were drugs with low P_app_ (*e.g.*, melagatran). All of the above emphasises the necessity for appropriate interpretation and implementation of complex physiological features of human kidney in the model to allow mechanistic prediction of CL_R_.

### 4.2 Validity of Caco-2 cell monolayers as *in vitro* model for renal tubular reabsorption

The ability to cross biological membranes is an important determinant of rate of absorption and rate and route of elimination (Smith et al., 2014, Varma et al., 2015). Existing *in vitro* proximal tubule models express a range of functional drug transporters, confounding measurement of passive permeability in the nephron (Brown et al., 2008, Fouda et al., 1990, Kunze et al., 2014). Although primary cultured collecting duct cells can be used for this purpose, these methods require a consistent supply of high quality kidney tissue (Trifillis and Kahng, 1990). Caco-2 cell monolayer assay performed in the presence of a transporter inhibitor cocktail is widely used to measure passive permeability and was therefore considered in the current work. Alternatively, permeability data obtained in MDCK cells (Avdeef and Tam, 2010, Irvine et al., 1999) can be considered for prediction of F_reab_. The application of the MDCK data in this model would require adequate implementation of the microvilli/surface area considerations highlighted, together with the use of transporter inhibitor cocktail in the *in vitro* assay to minimise the impact of differences in endogenous transporter expression on P_app_ data.

Beyond inter-system differences in permeability (*e.g.* Caco-2 vs. MDCK *vs.* nephron tubule cells), inter-laboratory variability has been widely reported for experimental data generated in either Caco-2 or MDCK cells (Artursson et al., 2001, Bentz et al., 2013). Reference compounds are often used to standardise *in vitro* assay data and minimise the impact of this variability on the subsequent IVIVE (Artursson et al., 2001, Hasegawa et al., 2003, Sohlenius-Sternbeck et al., 2012). The P_app_–F_reab_′ calibration approach proposed here can be used in that context. While this method resulted in the improved prediction of CL_R_ (Fig. 6 and Table 4), the choice of drugs used as reference dataset was fundamental. As such, the P_app_–F_reab_′ calibration proposed here should be applied with caution; further work is needed to refine and validate this approach using an external dataset of Caco-2 (or MDCK) P_app_ data.

The Caco-2 permeability data were measured under a pH-gradient in order to mimic the slightly acidic nature of urine typically found *in vivo*. The pH consideration was particularly important for basic drugs because the use of permeability data under iso-pH conditions resulted in systematic under-prediction of CL_R_ for this class (Supplementary Results, Fig. S4.3). However, despite the use of the pH-gradient, an apparent over-prediction of F_reab_ was still evident for some basic drugs, mostly those with moderate P_app_ (approx. 20–40 × 10^− 6^ cm/s). Two pH-related mechanisms could potentially contribute to this under-prediction. Firstly, a reduced fraction of unionised drug in acidic conditions may affect the permeation rate of the total (ionised and unionised) drug. Secondly, the concentration gradient of the unionised basic drug *in vivo* could be reduced in acidic conditions (due to water reabsorption), or in extreme cases reversed which would not be represented by the typical *in vitro* permeability assay. Additionally, it is important to consider that if left uncontrolled, urine pH can vary substantially in clinical studies (Özdemir et al., 2004), which can confound subsequent pharmacokinetic analyses and estimation of CL_R_ and F_reab_ used for model validation. Finally, potential contribution of tubular reabsorption via active transport *in vivo* via as yet unknown transporter-substrate interactions should not be disregarded.

### 4.3 Application of the mechanistic tubular reabsorption model and existing gaps

Use of the mechanistic tubular reabsorption model in conjunction with *in vitro* permeability data in a pure ‘bottom-up’ manner resulted in CL_R_ prediction accuracy comparable with quantitative structure-pharmacokinetic relationships and allometric approaches. In contrast to other methods, the added advantage of this IVIVE approach is the mechanistic insight into renal drug elimination because of the physiological nature of the model. This model provides solid foundation to inform future PBPK efforts towards understanding mechanisms behind changes in CL_R_ following pathophysiological changes in kidney, by accounting for the effects of factors such as age and renal impairment on the values of physiological parameters. For example, the lengths of each region of the tubule used in the model are representative of values reported for the healthy adult population (see Table S1.4 in the supplementary methods). Proximal tubule length has been reported to change with age (Darmady et al., 1973, Fetterman et al., 1965). Accounting for this, as well as any changes in other physiological parameters, could be used to investigate potential differences in tubular reabsorption in paediatric and geriatric populations.

Accuracy of CL_R_ prediction using the tubular reabsorption model was consistent across all ionisation groups, with a slightly higher bias seen for acidic drugs (Table 3). CL_R_ was over-predicted by more than 4-fold for three drugs (antipyrine, caffeine and isoxicam), which all had apparent F_reab_ values ≥ 0.99. For these drugs, predicted CL_R_ were very sensitive to even minor relative changes in F_reab_. However, the differences between predicted and observed CL_R_ for drugs with very high F_reab_ represented ≤ 15% of the plasma clearance of these drugs in healthy subjects, as they are also extensively metabolised by the liver (Huffman et al., 1974, Shaw et al., 1985, Tang-Liu et al., 1982). Therefore, potential errors in prediction of CL_R_ for extensively reabsorbed drugs were less likely to have a substantial impact on the prediction of the overall *in vivo* clearance for such drugs.

The dataset used for model assessment here included several drugs (*e.g.*, caffeine, antipyrine) which are extensively reabsorbed and are known to exhibit urine flow and pH dependent CL_R_ (Birkett and Miners, 1991, Mawer and Lee, 1968, Taylor and Blaschke, 1984). It is beyond capability (and purpose) of the model to describe/predict urine flow dependent CL_R_ quantitatively, as reported by some of the previous modelling efforts (Hall and Rowland, 1984, Neuhoff et al., 2013, Tang-Liu et al., 1983). However, modulation of the TFR_CD_ and urine flow rate parameters in the model can be used to indicate whether a drug is expected to exhibit urine flow dependent CL_R_, as shown by the sensitivity analysis presented in Supplementary Results, Fig. S4.5. It is anticipated that incorporation of the mechanistic prediction of tubular reabsorption into existing PBPK kidney models, *e.g.*, (Neuhoff et al., 2013), will allow for more accurate predictions of changes in tubular reabsorption and CL_R_ due to changes in urine flow rates.

IVIVE of renal metabolic clearance has been investigated in several studies (Gill et al., 2012, Gill et al., 2013, Knights et al., 2016). In contrast, prediction of CL_R,sec_ using ‘bottom-up’ approach is currently challenging due to lack of a ‘gold standard’ *in vitro* system and physiologically relevant scaling factors (Felmlee et al., 2013, Hsu et al., 2014, Kunze et al., 2014, Posada et al., 2015, Watanabe et al., 2011). Static models of CL_R,sec_ (Watanabe et al., 2011) could be used alongside the current reabsorption model for prediction of CL_R_; however, such an approach is unlikely to be adequate when metabolism is simultaneously involved. Consideration of metabolism and secretion was outside of the scope of the current model, but these elements could potentially be incorporated by expansion of the current model. While such expansion would not be beneficial for the majority of drugs investigated in the current study, it might be of interest for probenecid which has high apparent F_reab_ (0.955) and may undergo renal glucuronidation (Vree et al., 1993). Dynamic models, such as PBPK kidney models, which feature reabsorption, secretion and metabolism processes can be adapted/refined to incorporate mechanistic description of tubular reabsorption using the principles of the current model. Although some PBPK kidney models allow for metabolism-transport interplay to be investigated (Neuhoff et al., 2013), obtaining suitable clinical data to validate such models remains challenging (Tucker, 1981). Finally, considering a conservation of the tissue level organisation between mammalian species (*e.g.*, regional differentiation of nephron), the mechanistic tubular reabsorption model could be adapted for prediction of F_reab_ and CL_R_ in preclinical species, by accounting for specific differences in surface area and flow rate parameters.

### 4.4 Conclusion

A novel 5-compartment mechanistic tubular reabsorption model was developed for prediction of F_reab_ and CL_R_ from Caco-2 P_app_ data. A database of clinical CL_R_ values for 157 drugs was collated, as well as a comprehensive database of physiological parameters with relevance to IVIVE of renal excretion clearance. The mechanistic model successfully predicted CL_R_ for 45 chemically diverse drugs for which filtration or reabsorption appeared to be the dominant mechanism. In addition, empirical P_app_–F_reab_′ calibration method was proposed to account for inter-assay variability in permeability data. The physiological assumptions of the model represent an excellent basis for future studies, *e.g.*, simultaneous consideration of secretion and reabsorption for mechanistic predictions of CL_R_ and prediction of F_reab_ in different pathophysiological conditions (*e.g.*, renal impairment). Overall, the mechanistic model represents an important addition to the currently existing IVIVE toolbox, and a step towards enabling physiologically-based predictions of renal tubular reabsorption, and its contribution to CL_R_.

## Conflict of interest

The authors have no conflict of interest to declare.

## Figures and Tables

**Table 1:** Physiological parameter values used for tubular compartments in the minimal physiologically-based reabsorption model.

	TSA_i_ (m^2^)	TFR_i_ (mL/min)^a^
PT	6.1	81.6 (120–43.2)
LoH	0.16^b^	33.6 (43.2–24.0)
DT	0.21^b^	17.8 (24.0–11.6)
CD	0.045^b^, ^c^	6.3 (11.6–1.0)

a Values represent midpoint flow rates, ranges in parentheses represent flows at beginning and end of tubule regions.

b TSA_LoH_, TSA_DT_ and TSA_CD_ calculated accounting for microvilli.

c TSA_CD_ includes an exponential function for calculating surface area of inner medulla CD. Details are listed in the Supplementary Methods.

**Table 2:** *In vivo*, physico-chemical properties and *in vitro* data for 45 drugs used to assess the minimal model of tubular reabsorption. References for CL_R_ and indications of transporter affinity are provided in Supplementary Results Tables S2.1 and S3.1.

Drug	CL_R_ (mL/min)	F_reab_^a^	P_app_ (× 10^− 6^ cm/s)	f_u,p_	pK_a_ (acid)	pK_a_ (base)	Ionisation at pH 6.5^b^	LogD_7.4_	LogD_6.5_^c^	Indication of transporter affinity
Antipyrine	1.22	0.99	125	0.89	–	–	Neutral	0.11	0.11	N/A
Aprindine	1.28	0.78	31.8	0.05	–	9.95, 5.85^d^	Base	2.50	1.53	N/A
Atenolol	145	− 0.25	0.256	0.97	–	9.39	Base	− 2.65 c	− 3.55	OCT2
Betamethasone	9.50	0.78	26.5	0.36	–	–	Neutral	1.99	1.99	P-gp
Betaxolol	49.6	0.08	20.0	0.45	–	9.53	Base	0.63	− 0.27	N/A
Caffeine	1.06	0.99	81.1	0.67	–	–	Neutral	0.11 c	0.11	N/A
Chlorpheniramine	26.2	0.26	28.5	0.30	–	9.35, 5.15	Base	1.22	0.31	N/A
Chlorpropamide	0.56	0.91	88.1	0.05	4.69	–	Acid	− 0.33	0.56	N/A
Citalopram	65.2	− 0.09	20.9	0.50	–	9.57^d^	Base	1.55	0.65	N/A
Dapsone	5.50	0.83	49.7	0.27	–	–	Neutral	0.85	0.85	N/A
Difloxacin	4.54	0.94	74.6	0.62	5.81	7.39	Zwitterion	0.70	0.70	N/A
Doxepin	9.75	0.64	36.5	0.22	–	8.00^d^	Base	2.23	1.41	N/A
Fluconazole	15.7	0.85	36.3	0.86	–	–	Neutral	0.46	0.46	P-gp
Gabapentin	95.1	0.18	0.67	0.97	4.72^d^	10.27^d^	Zwitterion	− 1.10	− 1.79	OCTN1
Grepafloxacin	47.6	0.35	110	0.61	6.44^d^	8.74^d^	Zwitterion	0.50	0.50	P-gp
Imipramine	6.80	0.55	33.4	0.13	–	9.17	Base	2.49	1.60	N/A
Irbesartan	2.37	0.62	44.0	0.05	3.91	–	Acid	1.29	2.19	N/A
Isoxicam	0.02	1.00	142	0.04	3.84	–	Acid	− 0.20	0.70	N/A
Levetiracetam	39.4	0.64	15.7	0.90	–	–	Neutral	− 0.63 c	− 0.63	N/A
Linezolid	39.5	0.58	36.1	0.78	–	–	Neutral	0.67	0.67	N/A
Melagatran	114	− 0.02	0.145	0.93	2.12	11.62, 8.16^d^	Amphoteric	− 1.42	− 1.42	P-gp
Metoprolol	110	− 0.05	17.0	0.87	–	9.40	Base	− 0.29	− 1.19	OCT2
Metronidazole	9.64	0.92	64.7	0.98	–	–	Neutral	− 0.11	− 0.11	N/A
Mexiletine	71.6	− 0.25	43.8	0.48	–	9.13	Base	0.62	− 0.27	N/A
Moclobemide	3.42	0.94	64.4	0.50	–	6.53^d^	Base	1.60	1.34	N/A
Moxifloxacin	42.7	0.37	26.7	0.57	6.31	9.51	Zwitterion	− 0.24	− 0.24	P-gp, MRP2
Oxprenolol	9.35	0.44	30.0	0.14	–	9.60	Base	0.23	− 0.67	N/A
Oxytetracycline	90.8	0.02	0.850	0.77	3.27, 7.32, 9.11^d^	10.80^d^	Zwitterion	− 4.7^c^	− 4.70	P-gp
Pefloxacin	12.9	0.86	63.7	0.75	6.28	7.55	Zwitterion	0.30	0.35	P-gp
Prednisolone	34.3	0.10	19.7	0.32	–	–	Neutral	1.62^c^	1.62	P-gp
Prednisone	37.9	0.30	37.3	0.45	–	–	Neutral	1.25	1.25	Weak P-gp interaction
Probenecid	0.50	0.96	84.5	0.09	3.36	–	Acid	− 0.14	0.76	N/A
Propafenone	7.37	0.26	26.4	0.08	–	9.62^d^	Base	1.72	0.82	N/A
Propylthiouracil	3.20	0.83	80.8	0.16	8.01	–	Neutral	0.70	0.78	N/A
Ribavirin	109.9	0.08	1.55	1.00	–	–	Neutral	− 2.14^c^	− 2.14	N/A
Ropivacaine	2.73	0.55	32.3	0.05	–	8.10	Base	2.10	1.27	N/A
Sparfloxacin	21.7	0.73	49.8	0.67	6.31	8.94	Zwitterion	− 0.15	− 0.91	P-gp
Sulfamethoxazole	4.52	0.89	41.7	0.35	5.81	–	Acid	− 0.63	0.20	N/A
Tetracycline	86.4	0.05	1.10	0.76	5.06, 7.63, 8.77^d^	10.52^d^	Zwitterion	− 0.90	− 0.90	Weak OAT3 interaction
Theophylline	5.50	0.91	65.1	0.52	8.51	–	Neutral	− 0.09	− 0.06	N/A
Tocainide	67.5	0.18	19.0	0.69	–	7.75^d^	Base	0.03	− 0.73	N/A
Topiramate	15.1	0.86	46.2	0.87	9.22^d^	–	Neutral	0.60	0.61	MATE2K
Venlafaxine	80.0	0.09	27.6	0.73	–	9.64	Base	0.95	0.05	N/A
Verapamil	25.6	− 0.35	20.9	0.16	–	8.74	Base	2.61	1.73	P-gp, OCTN1, OCTN2
Voriconazole	1.57	0.97	96.5	0.42	–	–	Neutral	1.70	1.70	N/A

N/A No information available whether a drug was a substrate for renal drug transporters.

a Apparent negative F_reab_ are result of inclusion criteria (CR < 1.5) and were changed to 0 for graphical presentation.

b Drugs were classified using the predominant species (> 50%) at pH 6.5, using either measured or calculated pK_a_ data.

c LogD_6.5_ calculated from pK_a_ and LogD_7.4_, as described in the methods.

d LogD_7.4_ and pK_a_ values predicted using ACD (v.14.02) or obtained from BioBytes Masterfile (otherwise measured experimentally as described in the methods).

**Table 3:** Assessment of the physiologically-based tubular reabsorption model for prediction of CL_R_. Performance of the mechanistic model was assessed initially for all drugs with a measured Caco-2 P_app_ value with the exception of those that showed evidence of net secretion (clearance ratio > 1.5). Subsequently, the tubular reabsorption model was reassessed after excluding drugs currently identified as substrates for drug transporters expressed within kidney.

	R^2^	# (%) of drugs within 3-fold of observed CL_R_	gmfe	RMSE
*CL_R,filt_ only*				
All drugs (45)	0.38	26 (58%)	3.73	43.0
Neutral (15)	0.15	5 (33%)	6.69	63.4
Acid (5)	0.75	1 (20%)	16.45	18.0
Basic (16)	0.84	14 (88%)	1.80	18.0
Zwitterion (8)	0.41	5 (63%)	2.47	44.9
Amphoteric (1)	N/A	1 (100%)	1.02	2.3
*Minimal model*				
All drugs (45)	0.76	39 (87%)	1.96	20.9
Neutral (15)	0.86	13 (87%)	1.86	10.4
Acid (5)	0.84	4 (80%)	2.29	2.1
Basic (16)	0.79	14 (88%)	2.18	31.0
Zwitterion (8)	0.84	7 (88%)	1.73	18.3
Amphoteric (1)	N/A	1 (100%)	1.03	3.3
Non-substrates of renal transporters (29)	0.65	25 (86%)	2.07	19.3
Neutral (10)	0.95	8 (80%)	1.88	7.2
Acid (5)	0.84	4 (80%)	2.29	2.1
Basic (13)	0.76	12 (92%)	2.19	28.0
Zwitterion (1)	N/A	1 (100%)	1.66	3.0
Amphoteric (0)	N/A	N/A	N/A	N/A

**Table 4:** Assessment of the predictive performance of various CL_R_ prediction methods using gmfe and % predicted within 3-fold of observed CL_R_.

	gmfe (% predicted within 3-fold of observed)				
	Filtration only^a^	No correction for microvilli^b^	Proximal tubule only^c^	Reabsorption model^d^	P_app_–F_reab_′ calibration^e^
All drugs (n = 45)	3.73 (58%)	5.35 (27%)	2.17 (76%)	1.96 (87%)	1.65 (91%)
Low F_reab_ (n = 17)	1.17 (100%)	5.02 (35%)	1.59 (94%)	1.97 (88%)	1.34 (94%)
Medium F_reab_ (n = 12)	2.56 (75%)	8.52 (17%)	1.44 (92%)	1.90 (92%)	1.73 (92%)
High F_reab_ (n = 16)	16.86 (0%)	4.03 (25%)	4.11 (44%)	2.01 (81%)	1.98 (88%)

a CL_R,filt_ calculated using Eq. (7) in main text.

b No correction was made for surface area attributable to the presence/absence of microvilli when calculating CL_R,int,reab,i_.

c CL_R_ predicted using a model with only one tubular compartment representing proximal tubule (main contributor to reabsorption predicted by the model).

d CL_R_ predicted using tubular reabsorption model, as per Eq. (10), (12).

e CL_R_ predicted using the tubular reabsorption model after calibration of P_app_ data using Eq. (14), data are for all drugs including reference subset.

**Table 5:** Reference drugs used for calibration of Caco-2 P_app_ data.

Drug	CL_R_ (mL/min)	F_reab_	P_app,ref_ (× 10^− 6^ cm/s)	P_app,ref,calibrated_^a^ (× 10^− 6^ cm/s)	Ionisation at pH 6.5
Antipyrine	1.22	0.99	124.9	229.49	Neutral
Caffeine	1.06	0.99	81.12	94.06	Neutral
Chlorpropamide	0.56	0.91	88.12	111.60	Acid
Linezolid	39.5	0.58	36.1	17.65	Neutral
Oxprenolol	9.35	0.44	29.95	12.00	Base
Oxytetracycline	90.8	0.02	0.85	0.01	Zwitterion
Ribavirin	110	0.08	1.55	0.03	Neutral
Sparfloxacin	21.7	0.73	49.83	34.35	Zwitterion
Theophylline	5.50	0.91	65.13	59.75	Neutral
Tocainide	67.5	0.18	19	4.68	Base
Voriconazole	1.57	0.97	96.51	134.68	Neutral

a P_app,ref,calibrated_ calculated using Eq. (15) after fitting of the Hill equation to the Caco-2 P_app_ data and either observed or predicted (using minimal model of reabsorption) F_reab_.

## Appendix A Supplementary data

Supplementary Methods Image 27

Supplementary Results (Table S2.1) Image 28

Supplementary Results (Table S3.1) Image 29

Supplementary Results Image 30
