# Supplementary material for: Novel minimal physiologically-based model for the prediction of passive tubular reabsorption and renal excretion clearance
Source: Eur J Pharm Sci. 2016 Oct 30;94:59–71. doi: 10.1016/j.ejps.2016.03.018 (PMC5074076; doi:10.1016/j.ejps.2016.03.018)
Supplement: Supplementary file 1 — Supplementary Methods [file mmc1.docx]

**Supplementary Methods for the manuscript: “Novel minimal physiologically-based model for the prediction of passive tubular reabsorption and renal excretion clearance”**

Daniel Scotcher ^a^, Christopher Jones ^b^, Amin Rostami-Hodjegan ^a,c^ and Aleksandra Galetin ^a^

^a^ Centre for Applied Pharmacokinetic Research, Manchester Pharmacy School, University of Manchester, Manchester, United Kingdom

^b^ Oncology iMed, AstraZeneca, Alderley Park, United Kingdom

^c^ Simcyp Limited (a Certara Company), Sheffield, United Kingdom

Contents

[1. Tubular flow rates 2](#_Toc425162088)

[2. Tubular surface areas 4](#_Toc425162089)

[3. Derivation of P_app_ calibration 9](#_Toc425162090)

[4. Figure 10](#_Toc425162091)

[5. Tables 11](#_Toc425162092)

[6. References 16](#_Toc425162093)

# 1. Tubular flow rates

Physiology background

Major functions of kidney include electrolyte and water homeostasis. To achieve this, ultrafiltration of the plasma at the glomeruli result in the formation a relatively large volume of tubular filtrate. As the filtrate flows through the nephron tubules, much of the water, and other substances such as sodium and glucose, are reabsorbed through the nephron tubule, to alter the final composition of the urine. Water reabsorption across the nephron tubule is mediated by various aquaporin water channel proteins. Aquaporin channels are expressed at specific sections of the nephron tubule, namely the proximal tubule, the descending limb of the loop of Henle, and in response to vasopressin (a.k.a. antidiuretic hormone) at the connecting tubule, which for simplicity here is considered as part of the late distal tubule, and collecting duct. As such, water reabsorption varies along the nephron tubule.

Calculation of appropriate flow rates for each compartment in the model

Due to the difficulty of measuring filtrate flow rates along each section of the nephron tubule, data are scarce. In the absence of robust data, we assume that water reabsorption in each section of the nephron can be described linearly. Therefore the flow rates applied to each compartment were the ‘midpoint’ flow rate of that tubular section; that is the average of the flow rates at the beginning and end of each tubular section. As shown in table S1.1., values for the flow at the beginning and end of each nephron tubule section were taken as described for the MechKiM module in the SimCYP simulator ([Neuhoff et al., 2013](#_ENREF_27)). The fraction of water reabsorbed was calculated for each tubular section, assuming a glomerular filtration rate of 120 mL/ min. These values were compared with data from our own literature review, in order to highlight the level of confidence/ uncertainty in these values.

Tubular reabsorption of water literature review

The extent of water reabsorption can be experimentally determined *in vivo* through micropuncture techniques, whereby the tubular fluid to plasma concentration ratio of inulin (TF/P_inulin_) is measured at different points along the nephron tubule. The requirement for accessing the tubule sections while also maintaining the integrity of the kidney as a functional organ precludes micropuncture measurements being taken at sites other than in the cortex, immediately below the renal capsule. TF/P_inulin_ data are typically available only in animal species, and only at the proximal convoluted tubule (e.g. S1 and S2) and distal convoluted tubule which are found at the kidney surface. As the proximal straight tubule (S3), loop of Henle, and collecting ducts cannot be measured directly, tubular water reabsorption for these sections must be calculated using data available in the proximal convoluted tubule and distal convoluted tubule, along with glomerular filtration rate and urine flow rate data. Such TF/P_inulin_ measurements were not found for the human kidney; however the data for a study which used the micropuncture technique in rhesus monkeys is summarised in table S1.2 ([Bennett et al., 1968](#_ENREF_1)). Although data were available for rodent species, these were deemed not to be appropriate due to rodent urine being typically more concentrated than human (although such data could inform analogous models for rodent species).

Indirect measurements of filtrate reabsorption, by measuring urine composition in the presence/ absence of diuretics provide some further indications of water reabsorption potential of different sections of the tubule, but such interpretations depend on the broad assumption that the effects of diuretics are isolated to specific tubule sections, which may not reflect the “physiologically normal” state. For example furosemide is a loop diuretic which inhibit active chloride reabsorption in the thick ascending limb of the loop of Henle, and therefore should affect water reabsorption in the tubular sections distal to the ascending limb of the loop of Henle, namely the late distal tubule and the collecting duct. Following the administration of furosemide, to healthy subjects or cardiac surgery patients under ‘well hydrated’ conditions, urine flow increased by up to 20 mL/ min compared to the control conditions ([Hammarlund et al., 1985](#_ENREF_13); [Swärd et al., 2005](#_ENREF_40)). In the cardiac surgery patients, this was even accompanied by a reduced glomerular filtration rate following furosemide administration. These data suggest that the late DT and CD together have the capacity to reabsorb approximately 20 mL/ min, in agreement with the values used to calculate midpoint flow rates for the minimal model (table S1.1)

Further evidence is provided from studies involving nephrogenic diabetes insipidus patients, who have deficiencies in the expression, function or regulation of the aquaporin-2 channel ([Moeller et al., 2013](#_ENREF_25)). Aquaporin-2 is expressed in the late distal tubule and the collecting duct and is regulated by vasopressin ([Biner et al., 2002](#_ENREF_2); [Nielsen et al., 2002](#_ENREF_28)). Urine flow rates of up to 14 mL/ min have been reported for patients with nephrogenic diabetes insipidus, compared with a typical value of 1 mL/ min for healthy subjects ([Deen and Robben, 2010](#_ENREF_6); [Dicker and Eggleton, 1960](#_ENREF_7); [Jin et al., 2009](#_ENREF_17); [Weitzman and Kleeman, 1979](#_ENREF_44)). These data suggest an approximate fraction of water reabsorbed of 0.11, assuming glomerular filtration rate of 120 mL/ min, which is lower than the evidence presented above.

Further values of tubular flow rates and fractional water reabsorption for various nephron sections are reported throughout the scientific literature, as shown in table S1.2. These include values obtained from uncited secondary sources, ‘anecdotal’ assumed values used for calculations without citation of source, as well as values used as inputs for mathematical models of fluid transport in kidney and values generated by such models. Overall the tubular sections’ fraction water reabsorption assumed to calculate the filtrate flow rates are in agreement with the range of values found in the literature survey. The proximal tubule fraction water reabsorption (0.64) was at the lower end of the range of such values, whereas the loop of Henle fraction water reabsorption was at the higher end of the respective range of values.

# 2. Tubular surface areas

Physiology background

The nephron tubules consist of structurally different sections which are specifically adapted for various functional roles, reflected by a variety of different cell types. As such, factors which determine the surface area of any given section, such as tubule diameter, tubule length and microvilli, will vary between and even within tubule sections. Furthermore, these factors can vary between individuals. For example proximal convoluted tubule length increases from birth to adulthood ([Fetterman et al., 1965](#_ENREF_9)), and decreases after the age of 30-40 years ([Darmady et al., 1973](#_ENREF_5)), which is reflected by a decrease in the number of proximal tubule cells with age ([Goyal, 1982](#_ENREF_11)).

Another factor, which varies dramatically between individuals and determines the tubule surface area per kidney, is the number of nephrons per kidney. Nephron number has been linked to various demographic determinants, including age, ethnicity and birth weight. The nephron tubules remain as distinct parallel units from formation at the glomeruli through the proximal tubule, loop of Henle and distal tubule sections. At the collecting duct section the number of distinct tubules decreases as a result of the merging of multiple tubules. This process of merging occurs in two distinct phases, initially in the cortex, and then throughout the inner medulla, and has the net effect of reducing the surface area available for tubular reabsorption.

As indicated above, the number and size of microvilli vary between tubular cell types. Microvilli will increase the absolute surface areas of tubule sections available to mediate tubular reabsorption, compared with the surface area provided by a cylindrical tube. Caco-2 cell layers also have microvilli on their apical membranes, which is not taken into account during the calculation of P_app_ from assay data. Depending on whether the extent of microvilli is larger, smaller or equivalent in tubule sections compared with Caco-2 cell monolayers, the microvilli relative surface area expansion factor will be positive, negative or negligible.

Nephron tubule dimensions literature review

In order to calculate the surface area of a ‘cylindrical’ tubule section, the length and diameter of the tubule section are required. In order to support the chosen values with experimental data, a literature survey was performed. Such measurements are typically made using either during histological analysis of the kidney, or following the isolation of tubule sections which may be used for *in vitro* experiments.

Values for the tubule section length and diameter parameters were taken as those reported by Pitts (1974). These values were in agreement with the results of the wider literature survey, as shown in tables S1.3 and S1.4.

Nephron number literature review

The number of nephrons in human kidney has been extensively studied, typically through the measurement of the number of glomeruli ([Dunnill and Halley, 1973](#_ENREF_8); [Nyengaard and Bendtsen, 1992](#_ENREF_30)). In recent years a number of publications have reported human glomeruli number from the ‘Monash series’, which are summarised in a review paper ([Puelles et al., 2011](#_ENREF_37)). Although the number of nephrons varies widely between individuals, a value of 900,000 nephrons per kidney is reasonable for application in the reabsorption model for the proximal tubule, loop of Henle and distal tubule compartments.

Calculation of collecting duct surface area

The surface area of the collecting duct was calculated following a procedure similar to that published for a rodent collecting duct model ([Lory et al., 1983](#_ENREF_24)). The cortical collecting ducts (CCD) are each formed following the merging of approximately 10 tubules, as shown in figure S1.1 ([Hall, 2010](#_ENREF_12); [Kriz, 1981](#_ENREF_21); [Loffing and Kaissling, 2003](#_ENREF_23)). Assuming there are 900,000 nephrons per kidney, the surface area of the CCD is calculated following the assumption of 90,000 cylindrical tubules per kidney. Whereas the diameter of CCD (50 µm) is taken from the literature (see table S1.3), values for length were not found. Therefore it was assumed that the CCD is formed immediately adjacent to the renal capsule, and therefore the length is equivalent to the thickness of the cortex (8 mm; table S1.4). Although this assumption may slightly overestimate the surface area of the CCD, it is expected that this will be offset by the absence of the connecting tubule from this model, and is unlikely to have any noteworthy impact on overall F_reab_ predictions anyway.

The collecting ducts traverse the outer medulla (OMCD) without fusing, and therefore surface area was calculated as for CCD (length = outer medulla width = 2mm; diameter = 50 µm). Upon reaching the inner medulla, the collecting ducts (IMCD) undergo successive dichotomous fusions S1.1. In each human kidney, there are a reported 250 terminal/ papillary collecting ducts (tCD), known as ducts of Bellini, which are distributed across 8 – 18 renal papillae at the end s of the renal pyramids. tCDs empty from the papillae apices into the renal pelvis of each kidney ([Hall, 2010](#_ENREF_12); [Nielsen et al., 2012](#_ENREF_29)). Assuming an initial 90,000 IMCD, this suggests and average of 360 IMCD per tCD, requiring an average of 8.49 dichotomous fusion events per tCD. This is in agreement with a published value of 8 fusion events ([Kriz, 1981](#_ENREF_21)). Although the diameter, and therefore circumference, of individual IMCD increase as they extend towards the papilla apex, the total circumference decreases due to reducing number of CD. The total circumference of IMCD throughout the inner medulla at x mm from the papilla apex (C_x_) can be mathematically expressed as an exponential function (equation S1.1) ([Kainer, 1975](#_ENREF_18)). The IMCD surface area is the area under the curve, of this function between 0 and n, where n is the length of the IMCD (length = inner medulla width = 11 mm).

$C_{x}= \left( d_{0}\times{NCD}_{0} \times\pi\right)e^{\left( \left( \frac{x \times F}{n} \right) \times\ln\left( \frac{2}{{\frac{d_{0}}{d_{n}}}^{\frac{1}{F}}} \right) \right)}$ Equation S1.1 (Eq. 13 in main text)

Where d_0_ and d_n_ are the diameter of IMCD at the papilla apex and at the outer medulla-inner medulla boundary, NCD_0_ is the number of IMCDs at the papilla apex, and F is the number of fusion events.

The resulting exponential function is shown in figure S1.1, with the area under the curve (i.e. IMCD surface area) calculated by integration.

Microvilli expansion factors

As indicated above, microvilli are present, to differing extents, on the apical membranes of cells of both renal tubules and Caco-2 cell monolayers. Whereas Caco-2 cells and PT cells have extensive microvilli making up a brush border, the microvilli on the cells of other tubular sections of the nephron tend to be shorter and of fewer number ([Kriz, 1981](#_ENREF_21); [Møller and Skriver, 1985](#_ENREF_26); [Takahashi-Iwanaga et al., 1989](#_ENREF_41); [Welling et al., 1981](#_ENREF_45); [Welling and Welling, 1988](#_ENREF_47)). For example the PT microvilli are between approximately 1.5 and 2.5 µ, in length, whereas the microvilli in the descending limb of the LoH, the microvilli are approximately 0.4 µm in length ([Orloff and Berliner, 1973](#_ENREF_33)). The density and dimensions of microvilli may vary between species ([Kriz, 1981](#_ENREF_21)).

In rabbit PT the apical and basolateral membranes have similar surface areas, after accounting for microvilli (apical) and basolateral membrane folding ([Welling and Welling, 1988](#_ENREF_47)). In the absence of data on the apical membrane surface area associated with microvilli, human PT surface area was calculated using data published for the basolateral membrane of PT ([Møller and Skriver, 1985](#_ENREF_26)). Comparison with the surface area calculated using the assumption of a cylinder, as recently published for a model of PT reabsorption ([Kunze et al., 2014](#_ENREF_22)), revealed a 7.5-fold difference. Preliminary sensitivity analysis was performed during the development of the minimal model to assess the importance of changes in surface area of this order of magnitude on prediction of F_reab_. Using only the PT section as an exemplar, a 7.5 fold change of TSA_PT_ had a substantial impact on predicted F_reab,PT_’. Therefore, in order to account for the scarcity/ lack of microvilli in the LoH, DT and CD, verses presence of microvilli in Caco-2 cells, the TSA_i_ for these segments in the final model included a surface area correction factor as a 7.5 fold decrease on that calculated using the assumption of open cylinder.

Larger values of microvilli expansion factors have been reported for rabbit proximal tubule (15 to 40-fold), rat jejunum (24-fold) and human duodenum, jejunum and ileum (9.2, 14.1 and 15.7 fold), although a smaller value was calculated for the colon (6.4 fold), from which Caco-2 cells are derived ([Helander and Fändriks, 2014](#_ENREF_14); [Orloff and Berliner, 1973](#_ENREF_33); [Palay and Karlin, 1959](#_ENREF_35); [Welling and Welling, 1975](#_ENREF_46), [1988](#_ENREF_47)). Therefore a further sensitivity analysis on predicted F_reab_ and CL_R_ was performed by varying the microvilli correction factor from 1 to 50 in the final minimal model.

# 3. Derivation of P_app_ calibration

Starting point:

Hill function 1: Best fit to the predicted F_reab_’ by minimal reabsorption model vs. original P_app_ data (**P_1_**), with slope factor **a_1_** and F_0.5_ (P_app_ at which F­_reab_’ = 0.5) **b_1_.**

$${Hill}_{1}= \frac{{P_{1}}^{a_{1}}}{{b_{1}}^{a_{1}}+ {P_{1}}^{a_{1}}}$$

Hill function 2: Best fit of Hill function to F_reab_’ vs. original P_app_ data of reference drugs (**P_1_**), with slope factor **a_2_** and F_0.5_ **b_2_.**

$${Hill}_{2}= \frac{{P_{1}}^{a_{2}}}{{b_{2}}^{a_{2}}+ {P_{1}}^{a_{2}}}$$

Aim:

Hill function 3: Minimal reabsorption model vs. calibrated P_app_ data (**P_2_**), with slope factor **a_1_** and F_0.5_ **b_1_.**

$${Hill}_{3}= \frac{{P_{2}}^{a_{1}}}{{b_{1}}^{a_{1}}+ {P_{2}}^{a_{1}}}$$

**a_1_** and **b_1_** are obtained from the minimal model (i.e. Hill_1_). P_2_ must be obtained.

Rearrangement to obtain P_2_.

The calibration procedure will make Hill function 2 and Hill function 3 effectively equivalent.

$${{Hill}_{2}=Hill}_{3}$$

$$\frac{{P_{1}}^{a_{2}}}{{b_{2}}^{a_{2}}+ {P_{1}}^{a_{2}}}= \frac{{P_{2}}^{a_{1}}}{{b_{1}}^{a_{1}}+ {P_{2}}^{a_{1}}}$$

Rearrange to find P_2_.

$${P_{2}}^{a_{1}}= \left( {b_{1}}^{a_{1}}+ {P_{2}}^{a_{1}} \right)\left( \frac{{P_{1}}^{a_{2}}}{{b_{2}}^{a_{2}}+ {P_{1}}^{a_{2}}} \right)$$

$${P_{2}}^{a_{1}}= \frac{\left( \frac{{b_{1}}^{a_{1}}\times{P_{1}}^{a_{2}}}{{b_{2}}^{a_{2}}+ {P_{1}}^{a_{2}}} \right)}{1-\left( \frac{{P_{1}}^{a_{2}}}{{b_{2}}^{a_{2}}+ {P_{1}}^{a_{2}}} \right)}$$

$$P_{2}=\left( \frac{\left( {b_{1}}^{a_{1}}\times{P_{1}}^{a_{2}} \right)}{{b_{2}}^{a_{2}}} \right)^{\frac{1}{a_{1}}}$$

$$P_{2}=\frac{b_{1}\times{P_{1}}^{\left( \frac{a_{2}}{a_{1}} \right)}}{{b_{2}}^{\left( \frac{a_{2}}{a_{1}} \right)}}$$

# 4. Figure

**Figure S1.1. Exponential function used to calculate TSA_IMCD_.** Upon descending into the IMCD from the OMCD, the collecting ducts begin fusing, reducing the overall surface area. This reduction is described by an exponential function used to calculate the total circumference of IMCD as it descends towards the papilla apex, where the final urine pass through the ducts of Bellini (aka “terminal ducts”). The total circumference (mm) can also be considered as the surface area density (mm^2^/ mm IMCD length). The grey shaded area represents the area under the curve, which is the surface area of the IMCD.

# 5. Tables

Table S1.1. Regional filtrate flow rates along nephron tubule, from which midpoint TFR_i_ values were derived for model. The contribution of overall filtrate reabsorption from each region of nephron is also indicated

|  | Flow rate at beginning of tubule section | Flow rate at end of tubule section | TFR_i_ (mL/ min) | Fraction reabsorbed for section |
| --- | --- | --- | --- | --- |
| **PT** | 120 | 43.2 | 81.6 | 0.640 |
| **LoH** | 43.2 | 24.0 | 33.6 | 0.160 |
| **DT** | 24.0 | 11.6 | 17.8 | 0.103 |
| **CD** | 11.6 | 1.0 | 6.3 | 0.088 |

Table S1.2. Literature survey of tubular water reabsorption along nephron tubule

| **Fraction water reabsorption** | | | | | **Comments** | **Type of source/ Methods** | **Reference** |
| --- | --- | --- | --- | --- | --- | --- | --- |
| **PT** | **LoH** | **DT** | **DT+CD ^a^** | **CD** |  |  |  |
| 0.72 | 0.03 | 0.09 | - | 0.15 | Calculated from TF/P_inulin_ | Micropuncture; Rhesus monkey | ([Bennett et al., 1968](#_ENREF_1)) |
| 0.88-0.90 | - | - | - | - | - | Observations during maximal water diuresis/ NDI | ([Smith, 1947](#_ENREF_39)) |
| 0.80 | 0.05 | - | - | 0.14 | - | PT/LoH: assumed; CD: difference between presence and absence of vasopressin | ([Weitzman and Kleeman, 1979](#_ENREF_44)) |
| 0.67-0.88 | - | - | 0.13-0.20 | - | Cited Smith 1947 | Book | ([Pitts, 1974](#_ENREF_36)) |
| 0.67 | 0.22 | 0.01 – 0.08 | - | 0.00 - 0.02 | DT and CD ranges: water diuresis and antidiuresis | Simulation using model of human renal medulla | ([Cage et al., 1977](#_ENREF_3)) |
| 0.75 | 0.084 | - | 0.16 | - | - | Mathematical model | ([Uttamsingh et al., 1985](#_ENREF_42)) |
| 0.75 | 0.09 | - | 0.16 | - | Cited Uttamsingh et al 1985 | Mathematical model | ([Goldstein and Rypins, 1992](#_ENREF_10)) |
| 0.91 | - | - | - | 0.08 | (0.66 PCT; 0.25 PST) | Review article | ([Knauf and Mutschler, 1991](#_ENREF_19)) |
| 0.65 | 0.20 | - | 0.34 | - | - | Book chapter | ([Hall, 2010](#_ENREF_12)) |
| 0.63 | 0.17 | 0.11 | - | 0.09 | MechKiM; Cited Pitts 1974 and Hall 2010 | Book chapter; PBPK model | ([Neuhoff et al., 2013](#_ENREF_27)) |
| *0.63 – 0.91* | *0.03 – 0.22* | *0.01-0.11* | *0.13 – 0.34* | *0.00 – 0.14* | *Ranges* |  |  |
| *0.64* | *0.16* | *0.10* | *0.19* | *0.09* | *Minimal model* |  |  |

^a^ Some studies published a single value or range for the DT and CD in combination. The value for the minimal model was calculated by addition of values for DT and CD.

Table S1.3. Literature survey of tubular section diameters. Mean values, with ranges in square parenthesis.

| **Tubule diameter (µm)** | | | | **Comments** | **Type of source/ Methods** | **Reference** |
| --- | --- | --- | --- | --- | --- | --- |
| **PT** | **LoH** | **DT** | **CD** |  |  |  |
| [50 – 65] | [14 – 22] | [20 – 50] | 200 | Outer diameters; CD is at terminal end (ducts of Bellini) | Book | ([Pitts, 1974](#_ENREF_36)) |
| [51.0 – 79.7] | [28 – 53] | [51 – 60] | - | PT: Convoluted 75 - 79 | Maceration in HCl; 5 tubules from 1 kidney | ([Pai, 1935](#_ENREF_34)) |
| 41.5 | - | - | - | Luminal diameter | Morphometric analysis | ([Møller and Skriver, 1985](#_ENREF_26)) |
| [45.8 – 53.6] | [30.0 - 31.6] | [38.0 – 47.0] | [46.8 – 50.1] | Outer diameter; CD is for cortical collecting duct and outer medulla collecting duct. | Maceration in HCl | ([Chabardes et al., 1980](#_ENREF_4)) |
| 60 | 18 | 50 | 200 | MechKiM; Cited Pitts 1974 and Hall 2010 | Book chapter; PBPK model | ([Neuhoff et al., 2013](#_ENREF_27)) |
| - | - | - | 50 - 300 | Diameter of CD changes with distance from apex of renal pyramid, in agreement with two cited microscopy studies | Urography | ([Ohlson, 1989](#_ENREF_31)) |
| - | 12 - 16 | 16 | 20 | References not cited | Model of human renal medulla | ([Cage et al., 1977](#_ENREF_3)) |
| - | 22 [14 – 50] | - | - | - | Model of human renal medulla | ([Jacquez et al., 1976](#_ENREF_16)) |
| 30 | 20 | - | [25 – 35] | LoH is for thick ascending limb only; CD is range of cortical (25 µm) and medullary (35 µm) portions | Model of human nephron | ([Roman and Sias, 1986](#_ENREF_38)) |
| *30 – 79.7* | *12 - 53* | *16 - 60* | *25 – 300* | *Ranges* |  |  |
| *60* | *18* | *50* | *40 – 200 ^a^* | *Minimal model* |  |  |

^a^ Diameter of collecting ducts changes between cortex and outer medulla (50 µm), and the inner medulla. Within the inner medulla, as the collecting ducts progress toward the apex, number of collecting ducts reduces, while the diameter of the collecting ducts increase (from 40 to 200 µm).

Table S1.4. Literature survey of tubular section lengths. Mean values, with ranges in square parenthesis.

| **Tubule length (mm)** | | | | **Comments** | **Type of source/ Methods** | **Reference** |
| --- | --- | --- | --- | --- | --- | --- |
| **PT** | **LoH** | **DT** | **CD** |  |  |  |
| [12 – 24] | [6 – 32] | [2 – 9] | 22 | LoH: Thin limbs 0 – 14 mm; Thick ascending limbs 6 – 18. Total length exc. CD is 20 – 44 mm | Book | ([Pitts, 1974](#_ENREF_36)) |
| 19.7 [13.4 – 25.6] | - | - | - | - | Maceration in HCl; 104 tubules from 3 kidneys | ([Oliver and MacDowell, 1961](#_ENREF_32)) |
| [14.14 – 21.88] | [6.09 – 20.6] | [3.44 – 6.34] | - | Adult data and complete tubule sections only. LoH: Thin limbs 0.00 – 5.60 mm; Thick ascending limbs 6.09 – 15.00 mm. | Maceration in HCl; 5 tubules from 1 kidney | ([Pai, 1935](#_ENREF_34)) |
| 14.0 | [11.0 – 19.0] | 4.0 – 6.0 | - | LoH: Thin limbs 2.0 – 10.0 mm; Thick ascending limbs 9.0 mm. Total length exc CD is 30 - 38 mm | Peter, K. (1909); data reported as comparator | ([Pai, 1935](#_ENREF_34)) |
| 19.36 | - | - | - | Average for age range 20 – 39 years old | Maceration in HCl | ([Darmady et al., 1973](#_ENREF_5)) |
| - | 7.9 | 4.9 | - | LoH is for thick ascending limb only | Microdissection by collagenase digestion | ([Chabardes et al., 1980](#_ENREF_4)) |
| 18 | 7 | 5.5 | 22 | MechKiM; Cited Pitts 1974 and Hall 2010 | Book chapter; PBPK model | ([Neuhoff et al., 2013](#_ENREF_27)) |
| - | 20 | 8 | 10 | References not cited; Medulla only | Model of human renal medulla | ([Cage et al., 1977](#_ENREF_3)) |
| 14 | 20 | 12 | 22 | CD: Cortical CD 10 mm; Medullary CD 10 mm; Papillary CD 2 mm. | Model of human nephron | ([Koushanpour et al., 1971](#_ENREF_20)) |
| - | - | - | 20 | Width of outer medulla 10 mm; width of inner medulla 10 mm | Model of human renal medulla | ([Jacquez et al., 1976](#_ENREF_16)) |
| 20 | 5 - 30 | 10 | 15 | - | Model of renal cortex and medula | ([Jacquez et al., 1967](#_ENREF_15)) |
| 15 | 10 | - | 25 | LoH is for thick ascending limb only; CD is combination of cortical (10 mm) and medullary (15 mm) portions | Model of human nephron | ([Roman and Sias, 1986](#_ENREF_38)) |
| - | - | - | [19 – 21] | Cortex (6 – 8 mm), outer medulla (2 mm) and inner medulla (13 mm) | Estimated thickness of kidney section thicknesses | ([Wallace et al., 2002](#_ENREF_43)) |
| *12 – 25.6* | *5 - 32* | *2 - 12* | *10 - 22* | *Ranges* |  |  |
| *18* | *12* | *5.5* | *21^a^* | *Minimal model* |  |  |

^a^ Collecting ducts separated into three sub-sections, with lengths of 8, 2 and 11 mm for the cortex, outer medulla and inner medulla respectively ([Wallace et al., 2002](#_ENREF_43)).

# 6. References

Bennett, C., Brenner, B., Berliner, R., 1968. Micropuncture study of nephron function in the rhesus monkey. J Clin Invest. 47 (1), 203. DOI: 10.1172/JCI105710

Biner, H., Arpin-Bott, M., Loffing, J., Wang, X., Knepper, M., Hebert, S., Kaissling, B., 2002. Human cortical distal nephron: distribution of electrolyte and water transport pathways. J Am Soc Nephrol. 13 (4), 836.

Cage, P., Carson, E., Britton, K., 1977. A model of the human renal medulla. Comput Biomed Res. 10 (6), 561-584.

Chabardes, D., Gagnan-Brunette, M., Imbert-Teboul, M., Gontcharevskaia, O., Montegut, M., Clique, A., Morel, F., 1980. Adenylate cyclase responsiveness to hormones in various portions of the human nephron. J Clin Invest. 65 (2), 439.

Darmady, E., Offer, J., Woodhouse, M., 1973. The parameters of the ageing kidney. J Pathol. 109 (3), 195.

Deen, P., Robben, J., 2010. Potential of nonpeptide (ant) agonists to rescue vasopressin V2 receptor mutants for the treatment of X-linked nephrogenic diabetes insipidus. J Neuroendocrinol. 22 (5), 393.

Dicker, S., Eggleton, M.G., 1960. Hyaluronidase and antidiuretic activity in urine of man. J Physiol (Lond). 154 (2), 378.

Dunnill, M., Halley, W., 1973. Some observations on the quantitative anatomy of the kidney. J Pathol. 110 (2), 113.

Fetterman, G., Shuplock, N., Philipp, F., Gregg, H., 1965. The Growth And Maturation Of Human Glomeruli And Proximal Convolutions From Term To Adulthood: Studies By Microdissection. Pediatrics. 35, 601.

Goldstein, L., Rypins, E., 1992. A computer model of the kidney. Comput Methods Programs Biomed. 37 (3), 191.

Goyal, V., 1982. Changes with age in the human kidney. Exp Gerontol. 17 (5), 321.

Hall, J.E., 2010. Textbook of medical physiology / John E. Hall, Arthur C. Guyton, 12th ed. ed. Saunders Philadelphia, Pa.

Hammarlund, M., Odlind, B., Paalzow, L., 1985. Acute tolerance to furosemide diuresis in humans. Pharmacokinetic-pharmacodynamic modeling. J Pharmacol Exp Ther. 233 (2), 447.

Helander, H., Fändriks, L., 2014. Surface area of the digestive tract-revisited. Scand J Gastroenterol. 49 (6), 681.

Jacquez, J.A., Carnahan, B., Abbrecht, P., 1967. A model of the renal cortex and medulla. Math Biosci. 1 (2), 227-261.

Jacquez, J.A., Foster, D., Daniels, E., 1976. Solute concentration in the kidney—I. A model of the renal medulla and its limit cases. Math Biosci. 32 (3), 307-335.

Jin, X., Chen, Z., Cai, S., Chen, S., 2009. Nephrogenic diabetes insipidus with dilatation of bilateral renal pelvis, ureter and bladder. Scand J Urol Nephrol. 43 (1), 73.

Kainer, R., 1975. A geometric model of the rat kidney. Anat Embryol (Berl). 147 (1), 91.

Knauf, H., Mutschler, E., 1991. Pharmacodynamic and kinetic considerations on diuretics as a basis for differential therapy. Klin Wochenschr. 69 (6), 239.

Koushanpour, E., Tarica, R., Stevens, W., 1971. Mathematical simulation of normal nephron function in rat and man. J Theor Biol. 31 (2), 177.

Kriz, W., 1981. Structural organization of the renal medulla: comparative and functional aspects. Am J Physiol. 241 (1), R3.

Kunze, A., Huwyler, J., Poller, B., Gutmann, H., Camenisch, G., 2014. In vitro-in vivo extrapolation method to predict human renal clearance of drugs. J Pharm Sci. 103 (3), 994-1001. DOI: 10.1002/jps.23851

Loffing, J., Kaissling, B., 2003. Sodium and calcium transport pathways along the mammalian distal nephron: from rabbit to human. Am J Physiol Renal Physiol. 284 (4), F628.

Lory, P., Gilg, A., Horster, M., 1983. Renal countercurrent system: role of collecting duct convergence and pelvic urea predicted from a mathematical model. J Math Biol. 16 (3), 281.

Moeller, H.B., Rittig, S., Fenton, R.A., 2013. Nephrogenic Diabetes Insipidus: Essential Insights into the Molecular Background and Potential Therapies for Treatment. Endocr Rev. 34 (2), 278.

Møller, J., Skriver, E., 1985. Quantitative ultrastructure of human proximal tubules and cortical interstitium in chronic renal disease (hydronephrosis). Virchows Arch A Pathol Anat Histopathol. 406 (4), 389.

Neuhoff, S., Gaohua, L., Burt, H., Jamei, M., Li, L., Tucker, G.T., Rostami-Hodjegan, A., 2013. Accounting for transporters in renal clearance: towards a mechanistic kidney model (Mech KiM), in: Sugiyama, Y., Steffansen, B. (Eds.), Transporters in Drug Development. Springer, pp. 155-177.

Nielsen, S., Frøkiaer, J., Marples, D., Kwon, T., Agre, P., Knepper, M., 2002. Aquaporins in the kidney: from molecules to medicine. Physiol Rev. 82 (1), 205.

Nielsen, S., Kwon, T., Fenton, R., Praetorius, J., 2012. Anatomy of the Kidney, in: Taal, M., Chertow, G., Marsden, P., Skorecki, K., Yu, A., Brenner, B. (Eds.), Brenner and Rector's The Kidney, 9th ed. Elsevier, Philadelphia, PA, pp. 326-352.

Nyengaard, J., Bendtsen, T., 1992. Glomerular number and size in relation to age, kidney weight, and body surface in normal man. Anat Rec. 232 (2), 194.

Ohlson, L., 1989. Normal collecting ducts: visualization at urography. Radiology. 170 (1 Pt 1), 33.

Oliver, J., MacDowell, M., 1961. The structural and functional aspects of the handling of glucose by the nephrons and the kidney and their correlation by means of structural-functional equivalents. J Clin Invest. 40 (7), 1093.

Orloff, J., Berliner, R.W., 1973. Handbook of physiology. Section 8: renal physiology. American Physiological Society, Washington.

Pai, H.-C., 1935. Dissection of nephrons from the human kidney. J Anat. 69 (Pt 3), 344.

Palay, S.L., Karlin, L.J., 1959. An Electron Microscopic Study of the Intestinal Villus: II. The Pathway of Fat Absorption. J Biophys Biochem Cytol. 5 (3), 373.

Pitts, R.F., 1974. Physiology of the kidney and body fluids: an introductory text. Chicago: Year Book Medical Publishers.

Puelles, V., Hoy, W., Hughson, M., Diouf, B., Douglas-Denton, R., Bertram, J., 2011. Glomerular number and size variability and risk for kidney disease. Curr Opin Nephrol Hypertens. 20 (1), 7.

Roman, R.J., Sias, F.R., 1986. Network computer analysis of the human kidney. Math Modelling. 7 (5), 1045-1069.

Smith, H.W., 1947. The excretion of water. Bull N Y Acad Med. 23 (4), 177.

Swärd, K., Valsson, F., Sellgren, J., Ricksten, S., 2005. Differential effects of human atrial natriuretic peptide and furosemide on glomerular filtration rate and renal oxygen consumption in humans. Intensive Care Med. 31 (1), 79.

Takahashi-Iwanaga, H., Iwata, Y., Adachi, K., Fujita, T., 1989. The histotopography and ultrastructure of the thin limb of the Henle's loop: a scanning electron microscopic study of the rat kidney. Arch Histol Cytol. 52 (4), 395.

Uttamsingh, R., Leaning, M., Bushman, J., Carson, E., Finkelstein, L., 1985. Mathematical model of the human renal system. Med Biol Eng Comput. 23 (6), 525.

Wallace, D., Christensen, M., Reif, G., Belibi, F., Thrasher, B., Herrell, D., Grantham, J., 2002. Electrolyte and fluid secretion by cultured human inner medullary collecting duct cells. Am J Physiol Renal Physiol. 283 (6), F1337.

Weitzman, R.E., Kleeman, C.R., 1979. The clinical physiology of water metabolism: Part II: renal mechanisms for urinary concentration; diabetes insipidus. West J Med. 131 (6), 486.

Welling, L., Evan, A., Welling, D., 1981. Shape of cells and extracellular channels in rabbit cortical collecting ducts. Kidney Int. 20 (2), 211-222. DOI: 10.1038/ki.1981.123

Welling, L., Welling, D., 1975. Surface areas of brush border and lateral cell walls in the rabbit proximal nephron. Kidney Int. 8 (6), 343.

Welling, L., Welling, D., 1988. Relationship between structure and function in renal proximal tubule. J Electron Microsc Tech. 9 (2), 171-185. DOI: 10.1002/jemt.1060090205
