# Supplementary material for: Novel minimal physiologically-based model for the prediction of passive tubular reabsorption and renal excretion clearance
Source: Eur J Pharm Sci. 2016 Oct 30;94:59–71. doi: 10.1016/j.ejps.2016.03.018 (PMC5074076; doi:10.1016/j.ejps.2016.03.018)
Supplement: Supplementary file 2 — Supplementary Results (Table S2.1) [file mmc2.docx]

**Supplementary Results (Table S2.1) for the manuscript: “Novel minimal physiologically-based model for the prediction of passive tubular reabsorption and renal excretion clearance”**

Daniel Scotcher ^a^, Christopher Jones ^b^, Amin Rostami-Hodjegan ^a,c^ and Aleksandra Galetin ^a^

^a^ Centre for Applied Pharmacokinetic Research, Manchester Pharmacy School, University of Manchester, Manchester, United Kingdom

^b^ Oncology iMed, AstraZeneca, Alderley Park, United Kingdom

^c^ Simcyp Limited (a Certara Company), Sheffield, United Kingdom

**Table S2.1. Database of clinical CL_R_ values collated from the scientific literature. CL_R_ values were calculated using plasma and urine drug concentration data measured in the same healthy subjects, and were normalised for subject weight and body surface area where necessary.**

| **Drug** | **Overall weighted mean CL_R_**  **(mL/ min)** | **Overall weighted standard deviation CL_R_**  **(mL/ min)** | **Number of trials** | **Number of observations/ measurements** | **f_u,p_** | **References** |
| --- | --- | --- | --- | --- | --- | --- |
| Acebutolol | 222.8 | 58.2 | 4 | 36 | 0.88 | ([1-6](#_ENREF_1)) |
| Acecainide | 226.7 | 26.3 | 1 | 6 | 0.90 | ([7](#_ENREF_7), [8](#_ENREF_8)) |
| Acetaminophen | 11.5 | 0.3 | 2 | 20 | 0.81 | ([9](#_ENREF_9), [10](#_ENREF_10)) |
| Acyclovir | 275.7 | 93.2 | 5 | 33 | 0.77 | ([11-14](#_ENREF_11)) |
| Adefovir | 200.4 | 0.0 | 1 | 24 | 0.97 | ([15](#_ENREF_15), [16](#_ENREF_16)) |
| Allopurinol | 117.3 | 44.8 | 1 | 6 | 0.98 | ([17](#_ENREF_17), [18](#_ENREF_18)) |
| Almotriptan | 399.5 | 109.8 | 3 | 48 | 0.74 | ([19](#_ENREF_19), [20](#_ENREF_20)) |
| Amantadine | 219.2 | 113.6 | 1 | 9 | 0.33 | ([21](#_ENREF_21)) |
| Amifloxacin | 107.0 | 28.5 | 6 | 35 | 0.70 | ([22](#_ENREF_22), [23](#_ENREF_23)) |
| Amoxicillin | 167.0 | 41.3 | 2 | 15 | 0.83 | ([24-26](#_ENREF_24)) |
| Ampicillin | 163.6 | 42.8 | 2 | 15 | 0.80 | ([25](#_ENREF_25), [27](#_ENREF_27), [28](#_ENREF_28)) |
| Antipyrine | 1.2 | 0.4 | 2 | 14 | 0.89 | ([29-31](#_ENREF_29)) |
| Apalcillin | 29.0 | 6.1 | 1 | 10 | 0.14 | ([32](#_ENREF_32)) |
| Aprindine | 1.3 | 1.0 | 3 | 15 | 0.05 | ([33-35](#_ENREF_33)) |
| Atenolol | 145.3 | 48.1 | 10 | 97 | 0.97 | ([36-42](#_ENREF_36)) |
| Azlocillin | 83.3 | 17.1 | 3 | 29 | 0.64 | ([43-46](#_ENREF_43)) |
| Aztreonam | 71.7 | 10.9 | 5 | 44 | 0.45 | ([47](#_ENREF_47), [48](#_ENREF_48)) |
| Benzylpenicillin | 316.8 | 122.4 | 2 | 14 | 0.42 | ([28](#_ENREF_28), [45](#_ENREF_45), [49](#_ENREF_49)) |
| Betamethasone | 9.5 | 3.0 | 1 | 6 | 0.36 | ([50](#_ENREF_50)) |
| Betaxolol | 49.6 | 18.8 | 6 | 64 | 0.45 | ([51-53](#_ENREF_51)) |
| Bisoprolol | 157.0 | 40.1 | 5 | 36 | 0.67 | ([54-57](#_ENREF_54)) |
| Caffeine | 1.1 | 1.6 | 7 | 46 | 0.67 | ([58-62](#_ENREF_58)) |
| Captopril | 517.3 | 82.7 | 1 | 4 | 0.70 | ([63](#_ENREF_63), [64](#_ENREF_64)) ([63](#_ENREF_63)) |
| Carbenicillin | 127.0 | 29.2 | 2 | 8 | 0.47 | ([28](#_ENREF_28), [45](#_ENREF_45), [65](#_ENREF_65)) |
| Cefamandole | 162.3 | 31.2 | 1 | 8 | 0.32 | ([66](#_ENREF_66), [67](#_ENREF_67)) |
| Cefazolin | 53.0 | 14.7 | 8 | 59 | 0.21 | ([68-74](#_ENREF_68)) |
| Cefepime | 112.8 | 29.5 | 10 | 175 | 0.82 | ([75-78](#_ENREF_75)) |
| Cefixime | 32.5 | 5.4 | 20 | 257 | 0.37 | ([79](#_ENREF_79), [80](#_ENREF_80)) |
| Cefmetazole | 110.2 | 18.1 | 1 | 5 | 0.24 | ([81](#_ENREF_81), [82](#_ENREF_82)) |
| Cefodizime | 29.1 | 6.6 | 9 | 92 | 0.16 | ([83-90](#_ENREF_83)) |
| Cefonicid | 18.3 | 5.8 | 2 | 13 | 0.22 | ([91-93](#_ENREF_91)) |
| Cefoperazone | 23.2 | 8.0 | 1 | 8 | 0.12 | ([78](#_ENREF_78), [94-96](#_ENREF_94)) |
| Ceforanide | 46.8 | 4.4 | 4 | 28 | 0.19 | ([68](#_ENREF_68), [70](#_ENREF_70), [97](#_ENREF_97)) |
| Cefotaxime | 119.7 | 35.3 | 5 | 39 | 0.63 | ([78](#_ENREF_78), [98-102](#_ENREF_98)) |
| Cefotetan | 23.9 | 9.4 | 7 | 54 | 0.14 | ([69](#_ENREF_69), [103-107](#_ENREF_103)) |
| Cefotiam | 193.7 | 54.3 | 5 | 40 | 0.55 | ([108](#_ENREF_108), [109](#_ENREF_109)) |
| Cefpirome | 97.5 | 5.2 | 3 | 32 | 0.90 | ([110-113](#_ENREF_110)) |
| Ceftazidime | 98.0 | 15.9 | 5 | 41 | 0.87 | ([66](#_ENREF_66), [67](#_ENREF_67), [78](#_ENREF_78), [96](#_ENREF_96), [100](#_ENREF_100), [114](#_ENREF_114)) |
| Ceftizoxime | 102.2 | 39.6 | 3 | 29 | 0.51 | ([98](#_ENREF_98), [115](#_ENREF_115), [116](#_ENREF_116)) |
| Chloroquine | 167.2 | 97.8 | 2 | 13 | 0.41 | ([117-120](#_ENREF_117)) |
| Chlorpheniramine | 26.2 | 1.3 | 1 | 24 | 0.30 | ([121](#_ENREF_121), [122](#_ENREF_122)) |
| Chlorpropamide | 0.6 | 0.4 | 1 | 8 | 0.05 | ([123](#_ENREF_123)) |
| Chlorthalidone | 62.3 | 10.6 | 4 | 17 | 0.24 | ([124-127](#_ENREF_124)) |
| Cimetidine | 417.0 | 109.0 | 1 | 6 | 0.87 | ([31](#_ENREF_31), [128](#_ENREF_128), [129](#_ENREF_129)) |
| Ciprofloxacin | 339.5 | 99.9 | 10 | 111 | 0.71 | ([130-135](#_ENREF_130)) |
| Citalopram | 65.2 | 22.0 | 11 | 88 | 0.50 | ([136-141](#_ENREF_136)) |
| Clinafloxacin | 191.0 | 65.0 | 4 | 16 | 0.80 | ([142-144](#_ENREF_142)) |
| Dapsone | 5.5 | 6.3 | 1 | 7 | 0.27 | ([145](#_ENREF_145)) |
| Desipramine | 28.6 | 15.9 | 3 | 20 | 0.13 | ([146-149](#_ENREF_146)) |
| Dexrazoxane | 133.3 | 18.3 | 1 | 6 | 0.98 | ([150](#_ENREF_150), [151](#_ENREF_151)) |
| Difloxacin | 4.5 | 1.2 | 3 | 23 | 0.62 | ([134](#_ENREF_134), [152](#_ENREF_152), [153](#_ENREF_153)) |
| Digoxin | 151.1 | 74.3 | 18 | 160 | 0.72 | ([154-167](#_ENREF_154)) |
| Diltiazem | 48.0 | 15.9 | 1 | 6 | 0.26 | ([31](#_ENREF_31), [168-170](#_ENREF_168)) |
| Dofetilide | 273.7 | 38.7 | 1 | 12 | 0.36 | ([171-173](#_ENREF_171)) |
| Doxepin | 9.8 | 5.6 | 4 | 48 | 0.22 | ([174-176](#_ENREF_174)) |
| Enoxacin | 256.7 | 93.2 | 6 | 53 | 0.46 | ([134](#_ENREF_134), [177-180](#_ENREF_177)) |
| Enoximone | 3.4 | 2.2 | 7 | 41 | 0.21 | ([181-184](#_ENREF_181)) |
| Enprofylline | 272.6 | 71.5 | 3 | 21 | 0.57 | ([185-188](#_ENREF_185)) |
| Fexofenadine | 67.8 | 20.4 | 12 | 78 | 0.35 | ([23](#_ENREF_23), [189](#_ENREF_189), [190](#_ENREF_190)) ([189](#_ENREF_189), [190](#_ENREF_190)) |
| Fleroxacin | 67.5 | 17.2 | 2 | 12 | 0.74 | ([134](#_ENREF_134), [153](#_ENREF_153), [191](#_ENREF_191), [192](#_ENREF_192)) |
| Flucloxacillin | 90.9 | 0.2 | 2 | 20 | 0.05 | ([45](#_ENREF_45), [193](#_ENREF_193), [194](#_ENREF_194)) |
| Fluconazole | 15.7 | 5.2 | 3 | 22 | 0.86 | ([31](#_ENREF_31), [135](#_ENREF_135), [195-197](#_ENREF_195)) |
| Frovatriptan | 73.5 | 8.5 | 2 | 2 | 0.85 | ([198](#_ENREF_198)) |
| Furosemide | 97.4 | 42.1 | 3 | 25 | 0.02 | ([199-202](#_ENREF_199)) |
| Gabapentin | 95.1 | 30.8 | 7 | 90 | 0.97 | ([203-208](#_ENREF_203)) |
| Galantamine | 57.7 | 31.2 | 6 | 64 | 0.83 | ([209-212](#_ENREF_209)) |
| Garenoxacin | 41.8 | 11.9 | 5 | 29 | 0.25 | ([213](#_ENREF_213), [214](#_ENREF_214)) |
| Gatifloxacin | 168.4 | 46.4 | 8 | 54 | 0.81 | ([131](#_ENREF_131), [215-217](#_ENREF_215)) |
| Gefitinib | 8.6 | 3.4 | 4 | 16 | 0.07 | ([218-220](#_ENREF_218)) |
| Gemifloxacin | 176.8 | 43.8 | 9 | 57 | 0.40 | ([221-225](#_ENREF_221)) |
| Grepafloxacin | 47.6 | 17.7 | 6 | 74 | 0.61 | ([131](#_ENREF_131), [134](#_ENREF_134), [226](#_ENREF_226)) |
| Imipramine | 6.8 | 8.2 | 3 | 18 | 0.13 | ([120](#_ENREF_120), [147](#_ENREF_147), [227-230](#_ENREF_227)) |
| Irbesartan | 2.4 | 1.7 | 8 | 84 | 0.05 | ([231-233](#_ENREF_231)) |
| Isoxicam | 0.0 | 0.0 | 2 | 18 | 0.04 | ([234-237](#_ENREF_234)) |
| Lamivudine | 192.3 | 0.0 | 1 | 9 | 0.62 | ([23](#_ENREF_23), [238-241](#_ENREF_238)) |
| Lamotrigine | 2.5 | 1.5 | 3 | 27 | 0.45 | ([242-246](#_ENREF_242)) |
| Lenalidomide | 190.4 | 59.2 | 6 | 35 | 0.65 | ([247-249](#_ENREF_247)) |
| Levetiracetam | 39.4 | 0.7 | 2 | 2 | 0.90 | ([250](#_ENREF_250), [251](#_ENREF_251)) |
| Levofloxacin | 116.9 | 29.7 | 3 | 36 | 0.76 | ([131](#_ENREF_131), [135](#_ENREF_135), [153](#_ENREF_153), [216](#_ENREF_216), [252](#_ENREF_252), [253](#_ENREF_253)) |
| Linezolid | 39.5 | 9.6 | 10 | 60 | 0.78 | ([254-257](#_ENREF_254)) |
| Lomefloxacin | 155.8 | 29.1 | 3 | 24 | 0.85 | ([200](#_ENREF_200), [258-262](#_ENREF_258)) ([200](#_ENREF_200), [258](#_ENREF_258), [260](#_ENREF_260)) |
| Lorazepam | 0.3 | 0.1 | 3 | 23 | 0.11 | ([263-265](#_ENREF_263)) |
| Maraviroc | 177.2 | 27.8 | 3 | 24 | 0.25 | ([266](#_ENREF_266), [267](#_ENREF_267)) |
| MDMA | 215.0 | 91.7 | 2 | 16 | 0.65 | ([268-270](#_ENREF_268)) |
| Melagatran | 113.7 | 24.2 | 7 | 89 | 0.93 | ([271-274](#_ENREF_271)) |
| Memantine | 117.1 | 41.2 | 2 | 20 | 0.56 | ([275-278](#_ENREF_275)) |
| Mesna | 265.8 | 127.5 | 2 | 18 | 0.90 | ([279-281](#_ENREF_279)) |
| Metformin | 525.9 | 70.1 | 10 | 159 | 1.00 | ([282-286](#_ENREF_282)) |
| Methadone | 27.7 | 16.1 | 11 | 125 | 0.14 | ([287-292](#_ENREF_287)) |
| Metoprolol | 109.6 | 46.8 | 5 | 66 | 0.87 | ([293-295](#_ENREF_293)) |
| Metronidazole | 9.6 | 2.8 | 7 | 58 | 0.98 | ([296-299](#_ENREF_296)) |
| Mexiletine | 71.6 | 79.5 | 5 | 72 | 0.48 | ([300-304](#_ENREF_300)) |
| Moclobemide | 3.4 | 3.1 | 2 | 16 | 0.50 | ([305](#_ENREF_305), [306](#_ENREF_306)) |
| Morphine | 125.5 | 39.4 | 4 | 36 | 0.72 | ([203](#_ENREF_203), [307-310](#_ENREF_307)) |
| Moxalactam | 69.5 | 23.1 | 5 | 44 | 0.47 | ([311-317](#_ENREF_311)) |
| Moxifloxacin | 42.6 | 20.1 | 11 | 92 | 0.57 | ([131](#_ENREF_131), [318-322](#_ENREF_318)) |
| Nafcillin | 138.4 | 37.0 | 1 | 6 | 0.12 | ([28](#_ENREF_28), [45](#_ENREF_45), [323](#_ENREF_323)) |
| Ofloxacin | 184.4 | 42.0 | 8 | 100 | 0.76 | ([134](#_ENREF_134), [153](#_ENREF_153), [324](#_ENREF_324), [325](#_ENREF_325)) |
| Olmesartan | 11.4 | 2.1 | 3 | 21 | 0.01 | ([326-328](#_ENREF_326)) |
| Oseltamivir carboxylate | 231.7 | 47.2 | 4 | 96 | 0.97 | ([329](#_ENREF_329), [330](#_ENREF_330)) |
| Oxprenolol | 9.4 | 2.1 | 2 | 16 | 0.14 | ([39](#_ENREF_39), [331](#_ENREF_331)) |
| Oxytetracycline | 90.8 | 20.1 | 2 | 8 | 0.77 | ([332](#_ENREF_332), [333](#_ENREF_333)) |
| Pefloxacin | 12.9 | 6.1 | 8 | 33 | 0.75 | ([134](#_ENREF_134), [153](#_ENREF_153), [334-336](#_ENREF_334)) |
| Penciclovir | 439.6 | 118.2 | 4 | 77 | 0.80 | ([337](#_ENREF_337), [338](#_ENREF_338)) |
| Pilsicainide | 234.7 | 54.4 | 5 | 41 | 0.54 | ([339-341](#_ENREF_339)) |
| Pindolol | 210.9 | 77.4 | 6 | 44 | 0.41 | ([39](#_ENREF_39), [342-345](#_ENREF_342)) |
| Piperacillin | 130.7 | 54.8 | 3 | 24 | 0.67 | ([45](#_ENREF_45), [346](#_ENREF_346)) |
| Pravastatin | 414.6 | 104.5 | 3 | 24 | 0.39 | ([347-349](#_ENREF_347)) |
| Prednisolone | 34.3 | 19.4 | 4 | 24 | 0.32 | ([350-353](#_ENREF_350)) |
| Prednisone | 37.9 | 19.9 | 3 | 12 | 0.45 | ([352](#_ENREF_352), [354](#_ENREF_354)) |
| Probenecid | 0.5 | 0.3 | 6 | 18 | 0.09 | ([355-357](#_ENREF_355)) |
| Procainamide | 395.5 | 140.3 | 5 | 44 | 0.82 | ([358-363](#_ENREF_358)) |
| Promethazine | 5.9 | 2.5 | 1 | 7 | 0.10 | ([135](#_ENREF_135), [364](#_ENREF_364), [365](#_ENREF_365)) |
| Propafenone | 7.4 | 4.8 | 6 | 42 | 0.08 | ([366-370](#_ENREF_366)) |
| Propylthiouracil | 3.2 | 0.0 | 1 | 10 | 0.16 | ([371](#_ENREF_371), [372](#_ENREF_372)) |
| Pyrazinamide | 2.0 | 0.7 | 2 | 15 | 0.58 | ([373-375](#_ENREF_373)) |
| Quinidine | 85.2 | 17.8 | 9 | 61 | 0.25 | ([31](#_ENREF_31), [376-383](#_ENREF_376)) |
| Raltegravir | 55.0 | 9.8 | 9 | 54 | 0.17 | ([384](#_ENREF_384), [385](#_ENREF_385)) |
| Remoxipride | 35.6 | 24.7 | 7 | 84 | 0.16 | ([386-389](#_ENREF_386)) |
| Resveratrol | 16.6 | 20.9 | 4 | 40 | 0.02 | ([390-392](#_ENREF_390)) |
| Ribavirin | 109.9 | 30.6 | 4 | 15 | 1.00 | ([393-395](#_ENREF_393)) |
| Rifabutin | 58.4 | 0.0 | 1 | 11 | 0.24 | ([396](#_ENREF_396), [397](#_ENREF_397)) |
| Rifampin | 18.7 | 0.3 | 2 | 25 | 0.11 | ([398](#_ENREF_398), [399](#_ENREF_399)) |
| Risperidone | 41.5 | 18.6 | 8 | 35 | 0.15 | ([400-402](#_ENREF_400)) |
| Rivaroxaban | 49.8 | 15.6 | 3 | 44 | 0.06 | ([403-405](#_ENREF_403)) |
| Ropivacaine | 2.7 | 4.6 | 4 | 37 | 0.05 | ([406-410](#_ENREF_406)) |
| Rosuvastatin | 212.5 | 84.7 | 2 | 20 | 0.15 | ([411](#_ENREF_411), [412](#_ENREF_412)) |
| Rufloxacin | 16.8 | 7.6 | 9 | 90 | 0.41 | ([134](#_ENREF_134), [153](#_ENREF_153), [413-415](#_ENREF_413)) |
| Salbutamol | 289.9 | 76.7 | 4 | 50 | 0.93 | ([416](#_ENREF_416), [417](#_ENREF_417)) |
| Sematilide | 262.0 | 47.4 | 3 | 18 | 0.96 | ([418](#_ENREF_418), [419](#_ENREF_419)) |
| Sitagliptin | 371.2 | 89.3 | 14 | 110 | 0.63 | ([420-423](#_ENREF_420)) |
| Sparfloxacin | 21.7 | 10.4 | 9 | 101 | 0.67 | ([134](#_ENREF_134), [153](#_ENREF_153), [424-427](#_ENREF_424)) |
| Sulfamethoxazole | 4.5 | 4.6 | 4 | 27 | 0.35 | ([135](#_ENREF_135), [428-431](#_ENREF_428)) |
| Telbivudine | 116.7 | 37.1 | 2 | 20 | 0.97 | ([432-434](#_ENREF_432)) |
| Temafloxacin | 122.5 | 29.6 | 12 | 72 | 0.74 | ([153](#_ENREF_153), [435](#_ENREF_435), [436](#_ENREF_436)) |
| Temocillin | 39.8 | 12.5 | 2 | 8 | 0.18 | ([45](#_ENREF_45), [437](#_ENREF_437), [438](#_ENREF_438)) |
| Tenofovir | 220.0 | 0.0 | 1 | 24 | 0.99 | ([15](#_ENREF_15), [439](#_ENREF_439), [440](#_ENREF_440)) |
| Terodiline | 11.1 | 2.5 | 2 | 16 | 0.11 | ([441-444](#_ENREF_441)) |
| Tetracycline | 86.4 | 37.7 | 3 | 20 | 0.76 | ([441](#_ENREF_441)) |
| Theophylline | 5.5 | 2.4 | 2 | 25 | 0.52 | ([10](#_ENREF_10), [361](#_ENREF_361), [445-448](#_ENREF_445)) |
| Timolol | 125.1 | 61.3 | 2 | 19 | 0.40 | ([39](#_ENREF_39), [449](#_ENREF_449), [450](#_ENREF_450)) |
| Tinidazole | 11.3 | 1.1 | 3 | 20 | 0.94 | ([451-453](#_ENREF_451)) |
| Tizanidine | 66.2 | 35.1 | 2 | 30 | 0.70 | ([454-456](#_ENREF_454)) |
| Tocainide | 67.5 | 46.6 | 6 | 34 | 0.69 | ([457-461](#_ENREF_457)) |
| Tomopenem | 77.7 | 22.6 | 9 | 62 | 0.91 | ([462-464](#_ENREF_462)) |
| Topiramate | 15.1 | 5.3 | 10 | 64 | 0.87 | ([465-468](#_ENREF_465)) |
| Trimethoprim | 78.2 | 38.5 | 4 | 24 | 0.49 | ([430](#_ENREF_430), [469-471](#_ENREF_469)) |
| Trovafloxacin | 12.6 | 6.4 | 10 | 47 | 0.31 | ([134](#_ENREF_134), [153](#_ENREF_153), [322](#_ENREF_322), [472-474](#_ENREF_472)) |
| Valproic Acid | 0.2 | 0.2 | 2 | 12 | 0.07 | ([475](#_ENREF_475), [476](#_ENREF_476)) |
| Valsartan | 11.2 | 4.3 | 3 | 36 | 0.06 | ([477](#_ENREF_477), [478](#_ENREF_478)) |
| Varenicline | 118.6 | 40.2 | 10 | 91 | 0.84 | ([479-483](#_ENREF_479)) |
| Venlafaxine | 80.0 | 39.4 | 4 | 45 | 0.73 | ([216](#_ENREF_216), [484-488](#_ENREF_484)) |
| Verapamil | 25.6 | 12.3 | 2 | 12 | 0.16 | ([31](#_ENREF_31), [489](#_ENREF_489)) |
| Voriconazole | 1.6 | 0.8 | 11 | 111 | 0.42 | ([490-495](#_ENREF_490)) |
| Zanamivir | 89.1 | 40.3 | 11 | 93 | 0.94 | ([496-500](#_ENREF_496)) |
| Zidovudine | 258.1 | 141.2 | 2 | 8 | 0.84 | ([238](#_ENREF_238), [501-503](#_ENREF_501)) |
| Zopiclone | 13.0 | 10.1 | 6 | 78 | 0.36 | ([504-508](#_ENREF_504)) |

**References**

1. Roux A, Aubert P, Guedon J, Flouvat B. Pharmacokinetics of acebutolol in patients with all grades of renal failure. Eur J Clin Pharmacol. 1980;17(5):339.

2. Smith R, Warren D, Renwick A, George C. Acebutolol pharmacokinetics in renal failure. Br J Clin Pharmacol. 1983;16(3):253.

3. Lilja J, Raaska K, Neuvonen P. Effects of grapefruit juice on the pharmacokinetics of acebutolol. Br J Clin Pharmacol. 2005;60(6):659.

4. Roux A, Le Liboux A, Delhotal B, Gaillot J, Flouvat B. Pharmacokinetics in man of acebutolol and hydrochlorothiazide as single agents and in combination. Eur J Clin Pharmacol. 1983;24(6):801.

5. Coombs T, Coulson C, Smith V. Blood plasma binding of acebutolol and diacetolol in man. Br J Clin Pharmacol. 1980;9(4):395.

6. Kukes V, Gneushev E, Mamedov T, Gneusheva I. Acebutolol and diacetolol: Their binding to plasma proteins and erythrocytes and secretion with the saliva. Farmakol Toksikol. 1991;54(1):53.

7. Coyle J, Boudoulas H, Lima J. Acecainide pharmacokinetics in normal subjects of known acetylator phenotype. Biopharm Drug Dispos. 1991;12(8):599.

8. Connolly S, Kates R. Clinical pharmacokinetics of n-acetylprocainamide. Clin Pharmacokinet. 1982;7(3):206.

9. Critchley J, Critchley L, Anderson P, Tomlinson B. Differences in the single-oral-dose pharmacokinetics and urinary excretion of paracetamol and its conjugates between hong kong chinese and caucasian subjects. J Clin Pharm Ther. 2005;30(2):179.

10. Herrera A, Scott D, Lunte C. Microdialysis sampling for determination of plasma protein binding of drugs. Pharm Res. 1990;7(10):1077.

11. Brigden D, Bye A, Fowle A, Rogers H. Human pharmacokinetics of acyclovir (an antiviral agent) following rapid intravenous injection. J Antimicrob Chemoth. 1981;7(4):399.

12. Soul-Lawton J, Weatherley B, Posner J, Layton G, Peck R. Lack of interaction between valaciclovir, the l-valyl ester of aciclovir, and digoxin. Br J Clin Pharmacol. 1998;45(1):87.

13. De Bony F, Tod M, Bidault R, On N, Posner J, Rolan P. Multiple interactions of cimetidine and probenecid with valaciclovir and its metabolite acyclovir. Antimicrob Agents Chemother. 2002;46(2):458.

14. Blum M, Liao S, de Miranda P. Overview of acyclovir pharmacokinetic disposition in adults and children. Am J Med. 1982;73(1A):186.

15. Kearney B, Ramanathan S, Cheng A, Ebrahimi R, Shah J. Systemic and renal pharmacokinetics of adefovir and tenofovir upon coadministration. J Clin Pharmacol. 2005;45(8):935.

16. Cundy K, Barditch-Crovo P, Walker R, Collier A, Ebeling D, Toole J, et al. Clinical pharmacokinetics of adefovir in human immunodeficiency virus type 1-infected patients. Antimicrob Agents Chemother. 1995;39(11):2401.

17. Breithaupt B, Tittel M. Kinetics of allopurinol after single intravenous and oral doses. Noninteraction with benzbromarone and hydrochlorothiazide. Eur J Clin Pharmacol. 1982;22(1):77.

18. Elion G, Kovensky A, Hitchings G. Metabolic studies of allopurinol, an inhibitor of xanthine oxidase. Biochem Pharmacol. 1966;15(7):863.

19. Jansat J, Costa J, Salvà P, Fernandez F, Martinez-Tobed A. Absolute bioavailability, pharmacokinetics, and urinary excretion of the novel antimigraine agent almotriptan in healthy male volunteers. J Clin Pharmacol. 2002;42(12):1303.

20. Fleishaker J, Sisson T, Carel B, Azie N. Pharmacokinetic interaction between verapamil and almotriptan in healthy volunteers. Clin Pharmacol Ther. 2000;67(5):498.

21. Gaudry S, Sitar D, Smyth D, McKenzie J, Aoki F. Gender and age as factors in the inhibition of renal clearance of amantadine by quinine and quinidine. Clin Pharmacol Ther. 1993;54(1):23.

22. Cook J, Silverman M, Schelling D, Nix D, Schentag J, Brown R, et al. Multiple-dose pharmacokinetics and safety of oral amifloxacin in healthy volunteers. Antimicrob Agents Chemother. 1990;34(6):974.

23. Moffat AC, Osselton MD, Widdop B. Clarke's analysis of drugs and poisons. London: Pharmaceutical press; 2011.

24. Horber F, Frey F, Descoeudres C, Murray A, Reubi F. Differential effect of impaired renal function on the kinetics of clavulanic acid and amoxicillin. Antimicrob Agents Chemother. 1986;29(4):614.

25. Sjövall J, Westerlund D, Alván G. Renal excretion of intravenously infused amoxycillin and ampicillin. Br J Clin Pharmacol. 1985;19(2):191.

26. Brogden R, Heel R, Speight T, Avery G. Amoxycillin injectable: A review of its antibacterial spectrum, pharmacokinetics and therapeutic use. Drugs. 1979;18(3):169.

27. Blum R, Kohli R, Harrison N, Schentag J. Pharmacokinetics of ampicillin (2.0 grams) and sulbactam (1.0 gram) coadministered to subjects with normal and abnormal renal function and with end-stage renal disease on hemodialysis. Antimicrob Agents Chemother. 1989;33(9):1470.

28. Barza M, Weinstein L. Pharmacokinetics of the penicillins in man. Clin Pharmacokinet. 1976;1(4):297.

29. Staiger C, Schlicht F, Walter E, Gundert-Remy U, Hildebrandt R, de Vries J, et al. Effect of single and multiple doses of sulphinpyrazone on antipyrine metabolism and urinary excretion of 6-beta-hydroxycortisol. Eur J Clin Pharmacol. 1983;25(6):797.

30. Bax N, Lennard M, Tucker G. Inhibition of antipyrine metabolism by beta-adrenoceptor antagonists. Br J Clin Pharmacol. 1981;12(6):779.

31. Kochansky C, McMasters D, Lu P, Koeplinger K, Kerr H, Shou M, et al. Impact of pH on plasma protein binding in equilibrium dialysis. Mol Pharm. 2008;5(3):438.

32. Lode H, Elvers A, Koeppe P, Borner K. Comparative pharmacokinetics of apalcillin and piperacillin. Antimicrob Agents Chemother. 1984;25(1):105.

33. Kobari T, Itoh T, Hirakawa T, Namekawa H, Suzuki T, Satoh T, et al. Dose-dependent pharmacokinetics of aprindine in healthy volunteers. Eur J Clin Pharmacol. 1984;26(1):129.

34. Andreasen F, Husted S, Jakobsen P, Jensen E. The binding of aprindine to serum proteins with statistical considerations concerning the analysis of binding data. Acta Pharmacol Toxicol (Copenh). 1980;46(2):105.

35. Teirlynck O, Belpaire F, Andreasen F. Binding of aprindine and moxaprindine to human serum, alpha 1-acid glycoprotein and serum of healthy and diseased humans. Eur J Clin Pharmacol. 1982;21(5):427.

36. Barber H, Hawksworth G, Kitteringham N, Petersen J, Petrie J, Swann J. Protein binding of atenolol and propranolol to human serum albumin and in human plasma [proceedings]. Br J Clin Pharmacol. 1978;6(5):446P.

37. Mason W, Winer N, Kochak G, Cohen I, Bell R. Kinetics and absolute bioavailability of atenolol. Clin Pharmacol Ther. 1979;25(4):408.

38. McAinsh J, Holmes B, Smith S, Hood D, Warren D. Atenolol kinetics in renal failure. Clin Pharmacol Ther. 1980;28(3):302.

39. Belpaire F, Bogaert M, Rosseneu M. Binding of beta-adrenoceptor blocking drugs to human serum albumin, to alpha 1-acid glycoprotein and to human serum. Eur J Clin Pharmacol. 1982;22(3):253.

40. Fitzgerald J, Ruffin R, Smedstad K, Roberts R, McAinsh J. Studies on the pharmacokinetics and pharmacodynamics of atenolol in man. Eur J Clin Pharmacol. 1978;13(2):81.

41. Kirch W, Köhler H, Mutschler E, Schäfer M. Pharmacokinetics of atenolol in relation to renal function. Eur J Clin Pharmacol. 1981;19(1):65.

42. Mason W, Kochak G, Winer N, Cohen I. Effect of exercise on renal clearance of atenolol. J Pharm Sci. 1980;69(3):344.

43. Lander R, Henderson R, Pyszczynski D. Pharmacokinetic comparison of 5 g of azlocillin every 8 h and 4 g every 6 h in healthy volunteers. Antimicrob Agents Chemother. 1989;33(5):710.

44. Leroy A, Humbert G, Godin M, Fillastre J. Pharmacokinetics of azlocillin in subjects with normal and impaired renal function. Antimicrob Agents Chemother. 1980;17(3):344.

45. Nathwani D, Wood M. Penicillins. A current review of their clinical pharmacology and therapeutic use. Drugs. 1993;45(6):866.

46. Bergan T. Review of the pharmacokinetics and dose dependency of azlocillin in normal subjects and patients with renal insufficiency. J Antimicrob Chemoth. 1983;11:101.

47. Vinks AA, van Rossem RN, Mathôt RA, Heijerman HG, Mouton JW. Pharmacokinetics of aztreonam in healthy subjects and patients with cystic fibrosis and evaluation of dose-exposure relationships using monte carlo simulation. Antimicrob Agents Chemother. 2007;51(9):3049.

48. Swabb E, Sugerman A, McKinstry D. Multiple-dose pharmacokinetics of the monobactam azthreonam (sq 26,776) in healthy subjects. Antimicrob Agents Chemother. 1983;23(1):125.

49. Rumble R, Roberts M, Scott A. The effect of posture on the pharmacokinetics of intravenous benzylpenicillin. Eur J Clin Pharmacol. 1986;30(6):731.

50. Petersen M, Nation R, McBride W, Ashley J, Moore R. Pharmacokinetics of betamethasone in healthy adults after intravenous administration. Eur J Clin Pharmacol. 1983;25(5):643.

51. Ludden T, Boyle D, Gieseker D, Kennedy G, Crawford M, Ludden L, et al. Absolute bioavailability and dose proportionality of betaxolol in normal healthy subjects. J Pharm Sci. 1988;77(9):779.

52. Bianchetti G, Thiercelin J, Thenot J. Pharmacokinetics of betaxolol in middle aged patients. Eur J Clin Pharmacol. 1986;31(2):231.

53. McDevitt D. Comparison of pharmacokinetic properties of beta-adrenoceptor blocking drugs. Eur Heart J. 1987;8:9.

54. Bühring K, Sailer H, Faro H, Leopold G, Pabst J, Garbe A. Pharmacokinetics and metabolism of bisoprolol-14c in three animal species and in humans. J Cardiovasc Pharmacol. 1986;8:S21.

55. Le Coz F, Sauleman P, Poirier J, Cuche J, Midavaine M, Rames A, et al. Oral pharmacokinetics of bisoprolol in resting and exercising healthy volunteers. J Cardiovasc Pharmacol. 1991;18(1):28.

56. Leopold G, Pabst J, Ungethüm W, Bühring K. Basic pharmacokinetics of bisoprolol, a new highly beta 1-selective adrenoceptor antagonist. J Clin Pharmacol. 1986;26(8):616.

57. Horikiri Y, Suzuki T, Mizobe M. Pharmacokinetics and metabolism of bisoprolol enantiomers in humans. J Pharm Sci. 1998;87(3):289.

58. Birkett D, Miners J. Caffeine renal clearance and urine caffeine concentrations during steady state dosing. Implications for monitoring caffeine intake during sports events. Br J Clin Pharmacol. 1991;31(4):405.

59. Lelo A, Birkett D, Robson R, Miners J. Comparative pharmacokinetics of caffeine and its primary demethylated metabolites paraxanthine, theobromine and theophylline in man. Br J Clin Pharmacol. 1986;22(2):177.

60. Newton R, Broughton L, Lind M, Morrison P, Rogers H, Bradbrook I. Plasma and salivary pharmacokinetics of caffeine in man. Eur J Clin Pharmacol. 1981;21(1):45.

61. Blanchard J, Sawers S. Relationship between urine flow rate and renal clearance of caffeine in man. J Clin Pharmacol. 1983;23(4):134.

62. Blanchard J. Protein binding of caffeine in young and elderly males. J Pharm Sci. 1982;71(12):1415.

63. Sinhvi S, Duchin K, Willard D, McKinstry D, Migdalof B. Renal handling of captopril: Effect of probenecid. Clin Pharmacol Ther. 1982;32(2):182.

64. Lin S, Wei Y, Li M, Wang S. Effect of ethanol or/and captopril on the secondary structure of human serum albumin before and after protein binding. Eur J Pharm Biopharm. 2004;57(3):457.

65. Itoh T, Ishida M, Onuki Y, Tsuda Y, Shimada H, Yamada H. Stereoselective renal tubular secretion of carbenicillin. Antimicrob Agents Chemother. 1993;37(11):2327.

66. Wise E, Armstrong G, Brown R, Andrews J. The pharmacokinetics and tissue penetration of ceftazidime and cefamandole in healthy volunteers. J Antimicrob Chemoth. 1981;8:277.

67. Berkhout J, Visser L, van den Broek P, van de Klundert J, Mattie H. Clinical pharmacokinetics of cefamandole and ceftazidime administered by continuous intravenous infusion. Antimicrob Agents Chemother. 2003;47(6):1862.

68. Lee FH, Pfeffer M, Van Harken DR, Smyth RD, Hottendorf GH. Comparative pharmacokinetics of ceforanide (bl-s786r) and cefazolin in laboratory animals and humans. Antimicrob Agents Chemother. 1980;17(2):188.

69. Nakagawa K, Koyama M, Tachibana A, Komiya M, Kikuchi Y, Yano K. Pharmacokinetics of cefotetan (ym09330) in humans. Antimicrob Agents Chemother. 1982;22(6):935.

70. Smyth RD, Pfeffer M, Donald AG, Van Harken R, Hottendorf GH. Clinical pharmacokinetics and safety of high doses of ceforanide (bl-s786r) and cefazolin. Antimicrob Agents Chemother. 1979;16(5):615.

71. Lanao J, Vicente M, Dominguez-Gil A. Pharmacokinetics of cefazolin administered as a new drug delivery system in healthy volunteers. Biopharm Drug Dispos. 1988;9(4):377.

72. Vella-Brincat JW, Begg EJ, Kirkpatrick CM, Zhang M, Chambers ST, Gallagher K. Protein binding of cefazolin is saturable in vivo both between and within patients. Br J Clin Pharmacol. 2007;63(6):753.

73. Lavillaureix J, Brogard J, Pinget M, Ledoux F. Dosage adjustments of cefazolin according to the pharmacokinetics of this new cephalosporin. Infection. 1975;3(2):105.

74. Ohashi K, Tsunoo M, Tsuneoka K. Pharmacokinetics and protein binding of cefazolin and cephalothin in patients with cirrhosis. J Antimicrob Chemoth. 1986;17(3):347.

75. Barbhaiya R, Forgue S, Gleason C, Knupp C, Pittman K, Weidler D, et al. Pharmacokinetics of cefepime after single and multiple intravenous administrations in healthy subjects. Antimicrob Agents Chemother. 1992;36(3):552.

76. Barbhaiya R, Forgue S, Gleason C, Knupp C, Pittman K, Weidler D, et al. Safety, tolerance, and pharmacokinetic evaluation of cefepime after administration of single intravenous doses. Antimicrob Agents Chemother. 1990;34(6):1118.

77. Barbhaiya R, Forgue S, Shyu W, Papp E, Pittman K. High-pressure liquid chromatographic analysis of bmy-28142 in plasma and urine. Antimicrob Agents Chemother. 1987;31(1):55.

78. Kessler R, Bies M, Buck R, Chisholm D, Pursiano T, Tsai Y, et al. Comparison of a new cephalosporin, bmy 28142, with other broad-spectrum beta-lactam antibiotics. Antimicrob Agents Chemother. 1985;27(2):207.

79. Faulkner R, Yacobi A, Barone J, Kaplan S, Silber B. Pharmacokinetic profile of cefixime in man. Pediatr Infect Dis J. 1987;6(10):963.

80. Guay D, Meatherall R, Harding G, Brown G. Pharmacokinetics of cefixime (cl 284,635; fk 027) in healthy subjects and patients with renal insufficiency. Antimicrob Agents Chemother. 1986;30(3):485.

81. Ohkawa M, Orito M, Sugata T, Shimamura M, Sawaki M, Nakashita E, et al. Pharmacokinetics of cefmetazole in normal subjects and in patients with impaired renal function. Antimicrob Agents Chemother. 1980;18(3):386.

82. Tan J, Salstrom S, Signs S, Hoffman H, File T. Pharmacokinetics of intravenous cefmetazole with emphasis on comparison between predicted theoretical levels in tissue and actual skin window fluid levels. Antimicrob Agents Chemother. 1989;33(6):924.

83. Lenfant B, Namour F, Logeais C, Coussediere D, Rivault O, Bryskier A, et al. Pharmacokinetics of cefodizime following single doses of 0.5, 1.0, 2.0, and 3.0 grams administered intravenously to healthy volunteers. Antimicrob Agents Chemother. 1995;39(9):2037.

84. Loffreda A, Lampa E, Lucarelli C, Amorena M, Contaldi C, Calderaro V, et al. Pharmacokinetics of cefodizime in patients with various degrees of renal failure. Chemotherapy. 1999;45(1):1.

85. Scaglione F, Demartini G, Arcidiacono MM, Dugnani S, Fraschini F. Serum protein binding and extravascular diffusion of cefodizime and ceftriaxone. Clin Drug Invest. 1997;14(3):211-6.

86. Brockmeier D, Dagrosa E. Pharmacokinetic profile of cefodizime. Infection. 1992;20:S14.

87. Barré J. Pharmacokinetics of cefodizime: A review of the data on file. J Antimicrob Chemoth. 1990;26:95.

88. Bryskier A, Procyk T, Tremblay D, Lenfant B, Fourtillan J. Pharmacokinetics of cefodizime administered intravenously as a single-dose (1.0 and 2.0 g) to healthy adult volunteers. J Antimicrob Chemoth. 1990;26:65.

89. Bryskier A, Procyk T, Tremblay D, Lenfant B, Fourtillan J. The pharmacokinetics of cefodizime following intravenous and intramuscular administration of a single dose of 1.0 g. J Antimicrob Chemoth. 1990;26:59.

90. Conte Jr J. Pharmacokinetics of cefodizime in volunteers with normal or impaired renal function. J Clin Pharmacol. 1994;34(11):1066.

91. Barriere S, Hatheway G, Gambertoglio J, Lin E, Conte Jr J. Pharmacokinetics of cefonicid, a new broad-spectrum cephalosporin. Antimicrob Agents Chemother. 1982;21(6):935.

92. Benson J, Boudinot F, Pennell A, Cunningham F, DiPiro J. In vitro protein binding of cefonicid and cefuroxime in adult and neonatal sera. Antimicrob Agents Chemother. 1993;37(6):1343.

93. Fillastre J, Fourtillan J, Leroy A, Ramis N, Lefevre M, Reumont G, et al. Pharmacokinetics of cefonicid in uraemic patients. J Antimicrob Chemoth. 1986;18(2):203.

94. Gonik B, Feldman S, Pickering L, Doughtie C. Pharmacokinetics of cefoperazone in the parturient. Antimicrob Agents Chemother. 1986;30(6):874.

95. Guglielmo B, Flaherty J, Woods T, LaFollette G, Gambertoglio J. Pharmacokinetics of cefoperazone and tobramycin alone and in combination. Antimicrob Agents Chemother. 1987;31(2):264.

96. Lam Y, Duroux M, Gambertoglio J, Barriere S, Guglielmo B. Effect of protein binding on serum bactericidal activities of ceftazidime and cefoperazone in healthy volunteers. Antimicrob Agents Chemother. 1988;32(3):298.

97. Pfeffer M, Gaver R, Van Harken D. Human pharmacokinetics of a new braod-spectrum parenteral cephalosporin antibiotic, ceforanide. J Pharm Sci. 1980;69(4):398.

98. Gerding D, Van Etta L, Peterson L. Role of serum protein binding and multiple antibiotic doses in the extravascular distribution of ceftizoxime and cefotaxime. Antimicrob Agents Chemother. 1982;22(5):844.

99. Esmieu F, Guibert J, Rosenkilde H, Ho I, Le Go A. Pharmacokinetics of cefotaxime in normal human volunteers. J Antimicrob Chemoth. 1980;6:83.

100. Harding S, Monro A, Thornton J, Ayrton J, Hogg M. The comparative pharmacokinetics of ceftazidime and cefotaxime in healthy volunteers. J Antimicrob Chemoth. 1981;8:263.

101. Scaglione F, Raichi M, Fraschini F. Serum protein binding and extravascular diffusion of methoxyimino cephalosporins. Time courses of free and total concentrations of cefotaxime and ceftriaxone in serum and pleural exudate. J Antimicrob Chemoth. 1990;26:1.

102. Yee S, Nguyen A, Brown C, Savic R, Zhang Y, Castro R, et al. Reduced renal clearance of cefotaxime in asians with a low-frequency polymorphism of OAT3 (slc22a8). J Pharm Sci. 2013;102(9):3451.

103. Smith B, LeFrock J, Thyrum P, Doret B, Yeh C, Onesti G, et al. Cefotetan pharmacokinetics in volunteers with various degrees of renal function. Antimicrob Agents Chemother. 1986;29(5):887.

104. Martin C, Thomachot L, Albanese J. Clinical pharmacokinetics of cefotetan. Clin Pharmacokinet. 1994;26(4):248.

105. Carver P, Nightingale C, Quintiliani R. Pharmacokinetics and pharmacodynamics of total and unbound cefoxitin and cefotetan in healthy volunteers. J Antimicrob Chemoth. 1989;23(1):99.

106. Yates R, Adam H, Donnelly R, Houghton H, Charlesworth E, Laws E. Pharmacokinetics and tolerance of single intravenous doses of cefotetan disodium in male caucasian volunteers. J Antimicrob Chemoth. 1983;11:185.

107. Zimmerman J, Cohen A, Thyrum P. Absolute bioavailability and noncompartmental analysis of intravenous and intramuscular cefotan (cefotetan) in normal volunteers. J Clin Pharmacol. 1989;29(2):151.

108. Brisson A, Bryskier A, Millerioux L, Fourtillan J. Pharmacokinetics of cefotiam administered intravenously and intramuscularly to healthy adults. Antimicrob Agents Chemother. 1984;26(4):513.

109. Querol-Ferrer V, Zini R, Tillement J. The blood binding of cefotiam and cyclohexanol, metabolites of the prodrug cefotiam hexetil, in-vitro. J Pharm Pharmacol. 1991;43(12):863.

110. Bulitta J, Kinzig M, Landersdorfer C, Holzgrabe U, Stephan U, Sörgel F. Comparable population pharmacokinetics and pharmacodynamic breakpoints of cefpirome in cystic fibrosis patients and healthy volunteers. Antimicrob Agents Chemother. 2011;55(6):2927.

111. Maass L, Malerczyk V, Verho M. Pharmacokinetics of cefpirome (hr 810), a new cephalosporin derivative administered intramuscularly and intravenously to healthy volunteers. Infection. 1987;15(3):207.

112. Steiner I, Langenberger H, Marsik C, Mayer B, Fischer M, Georgopoulos A, et al. Effect of norepinephrine on cefpirome tissue concentrations in healthy subjects. J Antimicrob Chemoth. 2004;53(3):506.

113. Müller M, Rohde B, Kovar A, Georgopoulos A, Eichler H, Derendorf H. Relationship between serum and free interstitial concentrations of cefodizime and cefpirome in muscle and subcutaneous adipose tissue of healthy volunteers measured by microdialysis. J Clin Pharmacol. 1997;37(12):1108.

114. Ljungberg B, Nilsson-Ehle I. Comparative pharmacokinetics of ceftazidime in young, healthy and elderly, acutely ill males. Eur J Clin Pharmacol. 1988;34(2):179.

115. Kowalsky S, Echols R, Venezia A, Andrews E. Pharmacokinetics of ceftizoxime in subjects with various degrees of renal function. Antimicrob Agents Chemother. 1983;24(2):151.

116. LeBel M, Paone R, Lewis G. Effect of probenecid on the pharmacokinetics of ceftizoxime. J Antimicrob Chemoth. 1983;12(2):147.

117. Aderounmu A, Salako L, Lindström B, Walker O, Ekman L. Comparison of the pharmacokinetics of chloroquine after single intravenous and intramuscular administration in healthy africans. Br J Clin Pharmacol. 1986;22(5):559.

118. Ofori-Adjei D, Ericsson O, Lindström B, Sjöqvist F. Protein binding of chloroquine enantiomers and desethylchloroquine. Br J Clin Pharmacol. 1986;22(3):356.

119. Walker O, Birkett D, Alván G, Gustafsson L, Sjöqvist F. Characterization of chloroquine plasma protein binding in man. Br J Clin Pharmacol. 1983;15(3):375.

120. Onyeji C, Toriola T, Ogunbona F. Lack of pharmacokinetic interaction between chloroquine and imipramine. Ther Drug Monit. 1993;15(1):43.

121. Koch K, O'Connor-Semmes R, Davis I, Yin Y. Stereoselective pharmacokinetics of chlorpheniramine and the effect of ranitidine. J Pharm Sci. 1998;87(9):1097.

122. Peets E, Jackson M, Symchowicz S. Metabolism of chlorpheniramine maleate in man. J Pharmacol Exp Ther. 1972;180(2):364.

123. Neuvonen P, Kärkkäinen S, Lehtovaara R. Pharmacokinetics of chlorpropamide in epileptic patients: Effects of enzyme induction and urine pH on chlorpropamide elimination. Eur J Clin Pharmacol. 1987;32(3):297.

124. Dieterle W, Wagner J, Faigle J. Binding of chlorthalidone (hygroton®) to blood components in man. Eur J Clin Pharmacol. 1976;10(1):37-42.

125. Fleuren H, Thien T, Verwey-van Wissen C, van Rossum J. Absolute bioavailability of chlorthalidone in man: A cross-over study after intravenous and oral administration. Eur J Clin Pharmacol. 1979;15(1):35.

126. Mulley B, Parr G, Rye R. Pharmacokinetics of chlorthalidone. Dependence of biological half life on blood carbonic anhydrase levels. Eur J Clin Pharmacol. 1980;17(3):203.

127. Riess W, Dubach U, Burckhardt D, Theobald W, Vuillard P, Zimmerli M. Pharmacokinetic studies with chlorthalidone (hygroton) in man. Eur J Clin Pharmacol. 1977;12(5):375.

128. Gisclon L, Boyd R, Williams R, Giacomini K. The effect of probenecid on the renal elimination of cimetidine. Clin Pharmacol Ther. 1989;45(4):444.

129. Taylor D, Cresswell P, Bartlett D. The metabolism and elimination of cimetidine, a histamine h2-receptor antagonist, in the rat, dog, and man. Drug Metab Dispos. 1978;6(1):21.

130. Joos B, Ledergerber B, Flepp M, Bettex J, Lüthy R, Siegenthaler W. Comparison of high-pressure liquid chromatography and bioassay for determination of ciprofloxacin in serum and urine. Antimicrob Agents Chemother. 1985;27(3):353.

131. Lubasch A, Keller I, Borner K, Koeppe P, Lode H. Comparative pharmacokinetics of ciprofloxacin, gatifloxacin, grepafloxacin, levofloxacin, trovafloxacin, and moxifloxacin after single oral administration in healthy volunteers. Antimicrob Agents Chemother. 2000;44(10):2600.

132. Allard S, Kinzig M, Boivin G, Sörgel F, LeBel M. Intravenous ciprofloxacin disposition in obesity. Clin Pharmacol Ther. 1993;54(4):368.

133. Borner K, Höffken G, Lode H, Koeppe P, Prinzing C, Glatzel P, et al. Pharmacokinetics of ciprofloxacin in healthy volunteers after oral and intravenous administration. Eur J Clin Microbiol. 1986;5(2):179.

134. Zlotos G, Oehlmann M, Nickel P, Holzgrabe U. Determination of protein binding of gyrase inhibitors by means of continuous ultrafiltration. J Pharm Biomed Anal. 1998;18(4-5):847.

135. Waters N, Jones R, Williams G, Sohal B. Validation of a rapid equilibrium dialysis approach for the measurement of plasma protein binding. J Pharm Sci. 2008;97(10):4586.

136. Holford N. Clinical pharmacokinetics. Drug data handbook. 3rd ed. Auckland, N.Z: Adis International; 1998.

137. Herrlin K, Yasui-Furukori N, Tybring G, Widén J, Gustafsson LL, Bertilsson L. Metabolism of citalopram enantiomers in cyp2c19/CYP2D6 phenotyped panels of healthy swedes. Br J Clin Pharmacol. 2003;56(4):415.

138. Sidhu J, Priskorn M, Poulsen M, Segonzac A, Grollier G, Larsen F. Steady-state pharmacokinetics of the enantiomers of citalopram and its metabolites in humans. Chirality. 1997;9(7):686.

139. Joffe P, Larsen F, Pedersen V, Ring-Larsen H, Aaes-Jørgensen T, Sidhu J. Single-dose pharmacokinetics of citalopram in patients with moderate renal insufficiency or hepatic cirrhosis compared with healthy subjects. Eur J Clin Pharmacol. 1998;54(3):237.

140. Spigset O, Hägg S, Stegmayr B, Dahlqvist R. Citalopram pharmacokinetics in patients with chronic renal failure and the effect of haemodialysis. Eur J Clin Pharmacol. 2000;56(9-10):699.

141. Fredricson OK. Kinetics of citalopram in man; plasma levels in patients. Prog Neuropsychopharmacol Biol Psychiatry. 1982;6(3):311.

142. Randinitis EJ, Koup JR, Rausch G, Abel R, Bron NJ, Hounslow NJ, et al. Clinafloxacin pharmacokinetics in subjects with various degrees of renal function. Antimicrob Agents Chemother. 2001;45(9):2536.

143. Wise R, Ashby J, Andrews J. In vitro activity of pd 127,391, an enhanced-spectrum quinolone. Antimicrob Agents Chemother. 1988;32(8):1251.

144. Bron N, Dorr M, Mant T, Webb C, Vassos A. The tolerance and pharmacokinetics of clinafloxacin (ci-960) in healthy subjects. J Antimicrob Chemoth. 1996;38(6):1023.

145. May D, Porter J, Uetrecht J, Wilkinson G, Branch R. The contribution of n-hydroxylation and acetylation to dapsone pharmacokinetics in normal subjects. Clin Pharmacol Ther. 1990;48(6):619.

146. Rudorfer M, Lane E, Chang W, Zhang M, Potter W. Desipramine pharmacokinetics in chinese and caucasian volunteers. Br J Clin Pharmacol. 1984;17(4):433.

147. Ciraulo D, Barnhill J, Jaffe J. Clinical pharmacokinetics of imipramine and desipramine in alcoholics and normal volunteers. Clin Pharmacol Ther. 1988;43(5):509.

148. Spina E, Avenoso A, Campo G, Caputi A, Perucca E. The effect of carbamazepine on the 2-hydroxylation of desipramine. Psychopharmacology. 1995;117(4):413.

149. Spina E, Avenoso A, Campo G, Caputi A, Perucca E. Phenobarbital induces the 2-hydroxylation of desipramine. Ther Drug Monit. 1996;18(1):60.

150. Earhart R, Tutsch K, Koeller J, Rodriguez R, Robins H, Vogel C, et al. Pharmacokinetics of (+)-1, 2-di (3, 5-dioxopiperazin-1-yl) propane intravenous infusions in adult cancer patients. Cancer Res. 1982;42(12):5255.

151. Brier M, Gaylor S, McGovren J, Glue P, Fang A, Aronoff G. Pharmacokinetics of dexrazoxane in subjects with impaired kidney function. J Clin Pharmacol. 2011;51(5):731.

152. Granneman G, Snyder K, Shu V. Difloxacin metabolism and pharmacokinetics in humans after single oral doses. Antimicrob Agents Chemother. 1986;30(5):689.

153. Zlotos G, Bücker A, Kinzig-Schippers M, Sorgel F, Holzgrabe U. Plasma protein binding of gyrase inhibitors. J Pharm Sci. 1998;87(2):215.

154. Hedman A, Angelin B, Arvidsson A, Dahlqvist R. No effect of probenecid on the renal and biliary clearances of digoxin in man. Br J Clin Pharmacol. 1991;32(1):63.

155. Rengelshausen J, Göggelmann C, Burhenne J, Riedel K-D, Ludwig J, Weiss J, et al. Contribution of increased oral bioavailability and reduced nonglomerular renal clearance of digoxin to the digoxin–clarithromycin interaction. Br J Clin Pharmacol. 2003;56(1):32.

156. Sumner D, Russell A. Digoxin pharmacokinetics: Multicompartmental analysis and its clinical implications. Br J Clin Pharmacol. 1976;3(2):221.

157. Ewy G, Kapadia G, Yao L, Lullin M, Marcus F. Digoxin metabolism in the elderly. Circulation. 1969;39(4):449.

158. Belz G, Doering W, Munkes R, Matthews J. Interaction between digoxin and calcium antagonists and antiarrhythmic drugs. Clin Pharmacol Ther. 1983;33(4):410.

159. Ding R, Tayrouz Y, Riedel K, Burhenne J, Weiss J, Mikus G, et al. Substantial pharmacokinetic interaction between digoxin and ritonavir in healthy volunteers. Clin Pharmacol Ther. 2004;76(1):73.

160. Koytchev R, Alken R, Mayer O. Effect of diprafenone on the pharmacokinetics of digoxin. Eur J Clin Pharmacol. 1996;50(1-2):97.

161. Lukas D, De Martino A. Binding of digitoxin and some related cardenolides to human plasma proteins. J Clin Invest. 1969;48(6):1041.

162. Larsen F, Priskorn M, Overø K. Lack of citalopram effect on oral digoxin pharmacokinetics. J Clin Pharmacol. 2001;41(3):340.

163. Shoaf S, Ohzone Y, Ninomiya S, Furukawa M, Bricmont P, Kashiyama E, et al. In vitro p-glycoprotein interactions and steady-state pharmacokinetic interactions between tolvaptan and digoxin in healthy subjects. J Clin Pharmacol. 2011;51(5):761.

164. Tsutsumi K, Kotegawa T, Kuranari M, Otani Y, Morimoto T, Matsuki S, et al. The effect of erythromycin and clarithromycin on the pharmacokinetics of intravenous digoxin in healthy volunteers. J Clin Pharmacol. 2002;42(10):1159.

165. Kubitza D, Becka M, Roth A, Mueck W. Absence of clinically relevant interactions between rivaroxaban--an oral, direct factor xa inhibitor--and digoxin or atorvastatin in healthy subjects. J Int Med Res,. 2012;40(5):1688.

166. Hinderling P, Hartmann D. Pharmacokinetics of digoxin and main metabolites/derivatives in healthy humans. Ther Drug Monit. 1991;13(5):381.

167. Jalava K, Partanen J, Neuvonen P. Itraconazole decreases renal clearance of digoxin. Ther Drug Monit. 1997;19(6):609.

168. Boyd R, Chin S, Don-Pedro O, Verotta D, Sheiner L, Williams R, et al. The pharmacokinetics and pharmacodynamics of diltiazem and its metabolites in healthy adults after a single oral dose. Clin Pharmacol Ther. 1989;46(4):408.

169. Hung J, Hackett P, Gordon S, Ilett K. Pharmacokinetics of diltiazem in patients with unstable angina pectoris. Clin Pharmacol Ther. 1988;43(4):466.

170. Kwong T, Sparks J, Sparks C, editors. Lipoprotein and protein binding of the calcium channel blocker diltiazem. Proceedings of the Society for Experimental Biology and Medicine Society for Experimental Biology and Medicine; 1985.

171. Abel S, Nichols D, Brearley C, Eve M. Effect of cimetidine and ranitidine on pharmacokinetics and pharmacodynamics of a single dose of dofetilide. Br J Clin Pharmacol. 2000;49(1):64.

172. Mounsey J, DiMarco J. Cardiovascular drugs. Dofetilide. Circulation. 2000;102(21):2665.

173. Mahmood I. Interspecies scaling: Role of protein binding in the prediction of clearance from animals to humans. J Clin Pharmacol. 2000;40(12 Pt 2):1439.

174. Virtanen R, Iisalo E, Irjala K. Protein binding of doxepin and desmethyldoxepin. Acta Pharmacol Toxicol (Copenh). 1982;51(2):159.

175. Virtanen R, Scheinin M, Iisalo E. Single dose pharmacokinetics of doxepin in healthy volunteers. Acta Pharmacol Toxicol (Copenh). 1980;47(5):371.

176. Yan J, Hubbard J, McKay G, Korchinski E, Midha K. Absolute bioavailability and stereoselective pharmacokinetics of doxepin. Xenobiotica. 2002;32(7):615.

177. Bury R, Becker G, Kincaid-Smith P, Moulds R, Whitworth J. Elimination of enoxacin in renal disease. Clin Pharmacol Ther. 1987;41(4):434.

178. Chang T, Black A, Dunky A, Wolf R, Sedman A, Latts J, et al. Pharmacokinetics of intravenous and oral enoxacin in healthy volunteers. J Antimicrob Chemoth. 1988;21:49.

179. Somogyi A, Bochner F. The absorption and disposition of enoxacin in healthy subjects. J Clin Pharmacol. 1988;28(8):707.

180. Zhai S, Wei X, Parker B, Kunze K, Vestal R. Relation between plasma and saliva concentrations of enoxacin, ciprofloxacin, and theophylline. Ther Drug Monit. 1996;18(6):666.

181. Okerholm R, Chan K, Lang J, Thompson G, Ruberg S. Biotransformation and pharmacokinetic overview of enoximone and its sulfoxide metabolite. Am J Cardiol. 1987;60(5):21C.

182. Alken R, Belz G, Haegele K, Meinicke T, Schechter P. Kinetics of fenoximone, a new cardiotonic, in healthy subjects. Clin Pharmacol Ther. 1984;36(2):209.

183. Hook R, Boxenbaum H, Thompson G, Okerholm R. Human serum and plasma protein binding of enoximone and its sulfoxide metabolite. J Pharm Sci. 1988;77(12):1012.

184. Morita S, Sawai Y, Heeg J, Koike Y. Pharmacokinetics of enoximone after various intravenous administrations to healthy volunteers. J Pharm Sci. 1995;84(2):152.

185. Borgå O, Andersson K, Edholm L, Fagerström P, Lunell E, Persson C. Enprofylline kinetics in healthy subjects after single doses. Clin Pharmacol Ther. 1983;34(6):799.

186. Borgå O, Larsson R, Lunell E. Effects of probenecid on enprofylline kinetics in man. Eur J Clin Pharmacol. 1986;30(2):221.

187. Lunell E, Borgå O, Larsson R. Pharmacokinetics of enprofylline in patients with impaired renal function after a single intravenous dose. Eur J Clin Pharmacol. 1984;26(1):87.

188. Tegnér K, Borgå O, Svensson I. Protein binding of enprofylline. Eur J Clin Pharmacol. 1983;25(5):703.

189. Russell T, Stoltz M, Weir S. Pharmacokinetics, pharmacodynamics, and tolerance of single-and multiple-dose fexofenadine hydrochloride in healthy male volunteers. Clin Pharmacol Ther. 1998;64(6):612.

190. Yasui-Furukori N, Uno T, Sugawara K, Tateishi T. Different effects of three transporting inhibitors, verapamil, cimetidine, and probenecid, on fexofenadine pharmacokinetics. Clin Pharmacol Ther. 2005;77(1):17.

191. Shiba K, Saito A, Shimada J, Hori S, Kaji M, Miyahara T, et al. Renal handling of fleroxacin in rabbits, dogs, and humans. Antimicrob Agents Chemother. 1990;34(1):58.

192. Stuck A, Frey F, Heizmann P, Brandt R, Weidekamm E. Pharmacokinetics and metabolism of intravenous and oral fleroxacin in subjects with normal and impaired renal function and in patients on continuous ambulatory peritoneal dialysis. Antimicrob Agents Chemother. 1989;33(3):373.

193. Landersdorfer CB, Kirkpatrick CM, Kinzig M, Bulitta JB, Holzgrabe U, Sörgel F. Inhibition of flucloxacillin tubular renal secretion by piperacillin. Br J Clin Pharmacol. 2008;66(5):648.

194. Sutherland R, Croydon E, Rolinson G. Flucloxacillin, a new isoxazolyl penicillin, compared with oxacillin, cloxacillin, and dicloxacillin. Br Med J. 1970;4(5733):455.

195. Humphrey M, Jevons S, Tarbit M. Pharmacokinetic evaluation of uk-49,858, a metabolically stable triazole antifungal drug, in animals and humans. Antimicrob Agents Chemother. 1985;28(5):648.

196. Gross A, McLachlan A, Minns I, Beal J, Tett S. Simultaneous administration of a cocktail of markers to measure renal drug elimination pathways: Absence of a pharmacokinetic interaction between fluconazole and sinistrin, p-aminohippuric acid and pindolol. Br J Clin Pharmacol. 2001;51(6):547.

197. Sobue S, Tan K, Layton G, Leclerc V, Weil A. The effects of renal impairment on the pharmacokinetics and safety of fosfluconazole and fluconazole following a single intravenous bolus injection of fosfluconazole. Br J Clin Pharmacol. 2004;57(6):773.

198. Buchan P, Keywood C, Wade A, Ward C. Clinical pharmacokinetics of frovatriptan. Headache. 2002;42:S54.

199. Vree T, van den Biggelaar-Martea M, Verwey-van Wissen C. Probenecid inhibits the renal clearance of frusemide and its acyl glucuronide. Br J Clin Pharmacol. 1995;39(6):692.

200. Sudoh T, Fujimura A, Shiga T, Sasaki M, Harada K, Tateishi T, et al. Renal clearance of lomefloxacin is decreased by furosemide. Eur J Clin Pharmacol. 1994;46(3):267.

201. Chennavasin P, Seiwell R, Brater D, Liang W. Pharmacodynamic analysis of the furosemide-probenecid interaction in man. Kidney Int. 1979;16(2):187.

202. Ekblom M, Hammarlund-Udenaes M, Lundqvist T, Sjöberg P. Potential use of microdialysis in pharmacokinetics: A protein binding study. Pharm Res. 1992;9(1):155.

203. Eckhardt K, Ammon S, Hofmann U, Riebe A, Gugeler N, Mikus G. Gabapentin enhances the analgesic effect of morphine in healthy volunteers. Anesth Analg. 2000;91(1):185.

204. Hooper W, Kavanagh M, Herkes G, Eadie M. Lack of a pharmacokinetic interaction between phenobarbitone and gabapentin. Br J Clin Pharmacol. 1991;31(2):171.

205. Blum R, Comstock T, Sica D, Schultz R, Keller E, Reetze P, et al. Pharmacokinetics of gabapentin in subjects with various degrees of renal function. Clin Pharmacol Ther. 1994;56(2):154.

206. Urban T, Brown C, Castro R, Shah N, Mercer R, Huang Y, et al. Effects of genetic variation in the novel organic cation transporter, OCTN1, on the renal clearance of gabapentin. Clin Pharmacol Ther. 2008;83(3):416.

207. Radulovic L, Türck D, von Hodenberg A, Vollmer K, McNally W, DeHart P, et al. Disposition of gabapentin (neurontin) in mice, rats, dogs, and monkeys. Drug Metab Dispos. 1995;23(4):441.

208. Boyd R, Türck D, Abel R, Sedman A, Bockbrader H. Effects of age and gender on single-dose pharmacokinetics of gabapentin. Epilepsia. 1999;40(4):474.

209. Bickel U, Thomsen T, Weber W, Fischer J, Bachus R, Nitz M, et al. Pharmacokinetics of galanthamine in humans and corresponding cholinesterase inhibition. Clin Pharmacol Ther. 1991;50(4):420.

210. Tariot P. Current status and new developments with galantamine in the treatment of alzheimer's disease. Expert Opin Pharmacother. 2001;2(12):2027.

211. Zhao Q, Brett M, Van Osselaer N, Huang F, Raoult A, Van Peer A, et al. Galantamine pharmacokinetics, safety, and tolerability profiles are similar in healthy caucasian and japanese subjects. J Clin Pharmacol. 2002;42(9):1002.

212. Zhao Q, Iyer G, Verhaeghe T, Truyen L. Pharmacokinetics and safety of galantamine in subjects with hepatic impairment and healthy volunteers. J Clin Pharmacol. 2002;42(4):428.

213. Gajjar D, Bello A, Ge Z, Christopher L, Grasela D. Multiple-dose safety and pharmacokinetics of oral garenoxacin in healthy subjects. Antimicrob Agents Chemother. 2003;47(7):2256.

214. Van Wart S, Phillips L, Ludwig EA, Russo R, Gajjar DA, Bello A, et al. Population pharmacokinetics and pharmacodynamics of garenoxacin in patients with community-acquired respiratory tract infections. Antimicrob Agents Chemother. 2004;48(12):4766.

215. Nakashima M, Uematsu T, Kosuge K, Kusajima H, Ooie T, Masuda Y, et al. Single-and multiple-dose pharmacokinetics of am-1155, a new 6-fluoro-8-methoxy quinolone, in humans. Antimicrob Agents Chemother. 1995;39(12):2635.

216. Singh S, Mehta J. Measurement of drug-protein binding by immobilized human serum albumin-HPLC and comparison with ultrafiltration. J Chromatogr B Analyt Technol Biomed Life Sci. 2006;834(1-2):108.

217. Zhang X, Overholser B, Kays M, Sowinski K. Gatifloxacin pharmacokinetics in healthy men and women. J Clin Pharmacol. 2006;46(10):1154.

218. Swaisland H, Laight A, Stafford L, Jones H, Morris C, Dane A, et al. Pharmacokinetics and tolerability of the orally active selective epidermal growth factor receptor tyrosine kinase inhibitor zd1839 in healthy volunteers. Clin Pharmacokinet. 2001;40(4):297.

219. Li J, Brahmer J, Messersmith W, Hidalgo M, Baker S. Binding of gefitinib, an inhibitor of epidermal growth factor receptor-tyrosine kinase, to plasma proteins and blood cells: In vitro and in cancer patients. Invest New Drugs. 2006;24(4):291.

220. McKillop D, Hutchison M, Partridge E, Bushby N, Cooper C, Clarkson-Jones J, et al. Metabolic disposition of gefitinib, an epidermal growth factor receptor tyrosine kinase inhibitor, in rat, dog and man. Xenobiotica. 2004;34(10):917.

221. Allen A, Bygate E, Oliver S, Johnson M, Ward C, Cheon A, et al. Pharmacokinetics and tolerability of gemifloxacin (sb-265805) after administration of single oral doses to healthy volunteers. Antimicrob Agents Chemother. 2000;44(6):1604.

222. Allen A, Bygate E, Vousden M, Oliver S, Johnson M, Ward C, et al. Multiple-dose pharmacokinetics and tolerability of gemifloxacin administered orally to healthy volunteers. Antimicrob Agents Chemother. 2001;45(2):540.

223. Islinger F, Bouw R, Stahl M, Lackner E, Zeleny P, Brunner M, et al. Concentrations of gemifloxacin at the target site in healthy volunteers after a single oral dose. Antimicrob Agents Chemother. 2004;48(11):4246.

224. Landersdorfer CB, Kirkpatrick CM, Kinzig M, Bulitta JB, Holzgrabe U, Drusano GL, et al. Competitive inhibition of renal tubular secretion of gemifloxacin by probenecid. Antimicrob Agents Chemother. 2009;53(9):3902.

225. Gee T, Andrews J, Ashby J, Marshall G, Wise R. Pharmacokinetics and tissue penetration of gemifloxacin following a single oral dose. J Antimicrob Chemoth. 2001;47(4):431.

226. Efthymiopoulos C, Bramer S, Maroli A. Pharmacokinetics of grepafloxacin after oral administration of single and repeat doses in healthy young males. Clin Pharmacokinet. 1997;33:1.

227. Borgå O, Azarnoff D, Forshell G, Sjöqvist F. Plasma protein binding of tricyclic anti-depressants in man. Biochem Pharmacol. 1969;18(9):2135.

228. Brinkschulte M, Breyer-Pfaff U. Binding of tricyclic antidepressants and perazine to human plasma. Methodology and findings in normals. Naunyn Schmiedebergs Arch Pharmacol. 1979;308(1):1.

229. Nyberg G, Mårtensson E. Determination of free fractions of tricyclic antidepressants. Naunyn Schmiedebergs Arch Pharmacol. 1984;327(3):260.

230. Sutfin T, DeVane C, Jusko W. The analysis and disposition of imipramine and its active metabolites in man. Psychopharmacology. 1984;82(4):310.

231. Morsing P, Adler G, Brandt-Eliasson U, Karp L, Ohlson K, Renberg L, et al. Mechanistic differences of various at1-receptor blockers in isolated vessels of different origin. Hypertension. 1999;33(6):1406.

232. Marino M, Vachharajani N. Pharmacokinetics of irbesartan are not altered in special populations. J Cardiovasc Pharmacol. 2002;40(1):112.

233. Vachharajani N, Shyu W, Chando T, Everett D, Greene D, Barbhaiya R. Oral bioavailability and disposition characteristics of irbesartan, an angiotensin antagonist, in healthy volunteers. J Clin Pharmacol. 1998;38(8):702.

234. Esquivel M, Ogilvie R, East D, Shaw Jr D, Heathcote J. Pharmacokinetic disposition of isoxicam in hepatic cirrhosis. Clin Invest Med. 1987;10(5):363.

235. Bury R, Whitworth J, Saines D, Kincaid-Smith P, Moulds R. Effect of impairment of renal function on the accumulation and disposition of isoxicam. Eur J Clin Pharmacol. 1985;28(5):585.

236. Edwards I, Ferry D, Campbell A. Factors affecting the kinetics of two benzothiazine non-steroidal anti-inflammatory medicines, piroxicam and isoxicam. Eur J Clin Pharmacol. 1985;28(6):689.

237. Jolliet P, Simon N, Brée F, Urien S, Pagliara A, Carrupt P, et al. Blood-to-brain transfer of various oxicams: Effects of plasma binding on their brain delivery. Pharm Res. 1997;14(5):650.

238. Boffito M, Back D, Blaschke T, Rowland M, Bertz R, Gerber J, et al. Protein binding in antiretroviral therapies. AIDS Res Hum Retroviruses. 2003;19(9):825.

239. Johnson M, Verpooten G, Daniel M, Plumb R, Moss J, Van Caesbroeck D, et al. Single dose pharmacokinetics of lamivudine in subjects with impaired renal function and the effect of haemodialysis. Br J Clin Pharmacol. 1998;46(1):21.

240. Johnson M, Moore K, Yuen G, Bye A, Pakes G. Clinical pharmacokinetics of lamivudine. Clin Pharmacokinet. 1999;36(1):41.

241. Votano J, Parham M, Hall L, Hall L, Kier L, Oloff S, et al. Qsar modeling of human serum protein binding with several modeling techniques utilizing structure-information representation. J Med Chem. 2006;49(24):7169.

242. Wootton R, Soul-Lawton J, Rolan P, Sheung C, Cooper J, Posner J. Comparison of the pharmacokinetics of lamotrigine in patients with chronic renal failure and healthy volunteers. Br J Clin Pharmacol. 1997;43(1):23.

243. Yuen A, Land G, Weatherley B, Peck A. Sodium valproate acutely inhibits lamotrigine metabolism. Br J Clin Pharmacol. 1992;33(5):511.

244. Rambeck B, Wolf P. Lamotrigine clinical pharmacokinetics. Clin Pharmacokinet. 1993;25(6):433.

245. Cohen A, Land G, Breimer D, Yuen W, Winton C, Peck A. Lamotrigine, a new anticonvulsant: Pharmacokinetics in normal humans. Clin Pharmacol Ther. 1987;42(5):535.

246. Ebert U, Thong N, Oertel R, Kirch W. Effects of rifampicin and cimetidine on pharmacokinetics and pharmacodynamics of lamotrigine in healthy subjects. Eur J Clin Pharmacol. 2000;56(4):299.

247. Scott L, Lyseng-Williamson K. Spotlight on lenalidomide in relapsed or refractory multiple myeloma. BioDrugs. 2011;25(5):333.

248. Chen N, Kasserra C, Reyes J, Liu L, Lau H. Single-dose pharmacokinetics of lenalidomide in healthy volunteers: Dose proportionality, food effect, and racial sensitivity. Cancer Chemother Pharmacol. 2012;70(5):717.

249. Chen N, Lau H, Kong L, Kumar G, Zeldis J, Knight R, et al. Pharmacokinetics of lenalidomide in subjects with various degrees of renal impairment and in subjects on hemodialysis. J Clin Pharmacol. 2007;47(12):1466.

250. Radtke R. Pharmacokinetics of levetiracetam. Epilepsia. 2001;42:24.

251. Patsalos P. Pharmacokinetic profile of levetiracetam: Toward ideal characteristics. Pharmacol Ther. 2000;85(2):77.

252. Neckel U, Joukhadar C, Frossard M, Jäger W, Müller M, Mayer BX. Simultaneous determination of levofloxacin and ciprofloxacin in microdialysates and plasma by high-performance liquid chromatography. Anal Chim Acta. 2002;463(2):199-206.

253. Chien S, Rogge M, Gisclon L, Curtin C, Wong F, Natarajan J, et al. Pharmacokinetic profile of levofloxacin following once-daily 500-milligram oral or intravenous doses. Antimicrob Agents Chemother. 1997;41(10):2256.

254. Brier ME, Stalker DJ, Aronoff GR, Batts DH, Ryan KK, O'Grady M, et al. Pharmacokinetics of linezolid in subjects with renal dysfunction. Antimicrob Agents Chemother. 2003;47(9):2775.

255. Buerger C, Plock N, Dehghanyar P, Joukhadar C, Kloft C. Pharmacokinetics of unbound linezolid in plasma and tissue interstitium of critically ill patients after multiple dosing using microdialysis. Antimicrob Agents Chemother. 2006;50(7):2455.

256. Slatter J, Stalker D, Feenstra K, Welshman I, Bruss J, Sams J, et al. Pharmacokinetics, metabolism, and excretion of linezolid following an oral dose of [(14) c] linezolid to healthy human subjects. Drug Metab Dispos. 2001;29(8):1136.

257. Stalker DJ, Jungbluth GL, Hopkins NK, Batts DH. Pharmacokinetics and tolerance of single-and multiple-dose oral or intravenous linezolid, an oxazolidinone antibiotic, in healthy volunteers. J Antimicrob Chemother. 2003;51(5):1239-46.

258. Blum R, Schultz R, Schentag J. Pharmacokinetics of lomefloxacin in renally compromised patients. Antimicrob Agents Chemother. 1990;34(12):2364-8.

259. Okezaki E, Terasaki T, Nakamura M, Nagata O, Kato H, Tsuji A. Serum protein binding of lomefloxacin, a new antimicrobial agent, and its related quinolones. J Pharm Sci. 1989;78(6):504.

260. Sudoh T, Fujimura A, Harada K, Sunaga K, Ohmori M, Sakamoto K. Effect of ranitidine on renal clearance of lomefloxacin. Eur J Clin Pharmacol. 1996;51(1):95-8.

261. Turnidge J. Pharmacokinetics and pharmacodynamics of fluoroquinolones. Drugs. 1999;58:29.

262. Wise R, Andrews J, Ashby J, Matthews R. In vitro activity of lomefloxacin, a new quinolone antimicrobial agent, in comparison with those of other agents. Antimicrob Agents Chemother. 1988;32(5):617.

263. Abernethy D, Greenblatt D, Divoll M, Ameer B, Shader R. Differential effect of cimetidine on drug oxidation (antipyrine and diazepam) vs. Conjugation (acetaminophen and lorazepam): Prevention of acetaminophen toxicity by cimetidine. J Pharmacol Exp Ther. 1983;224(3):508.

264. Herman RJ, Van Pham JD, Szakacs CB. Disposition of lorazepam in human beings: Enterohepatic recirculation and first-pass effect. Clin Pharmacol Ther. 1989;46(1):18-25.

265. Samara EE, Granneman RG, Witt GF, Cavanaugh JH. Effect of valproate on the pharmacokinetics and pharmacodynamics of lorazepam. J Clin Pharmacol. 1997;37(5):442-50.

266. Abel S, Russell D, Whitlock LA, Ridgway CE, Nedderman AN, Walker DK. Assessment of the absorption, metabolism and absolute bioavailability of maraviroc in healthy male subjects. Br J Clin Pharmacol. 2008;65(s1):60-7.

267. Walker D, Abel S, Comby P, Muirhead G, Nedderman A, Smith D. Species differences in the disposition of the ccr5 antagonist, uk-427,857, a new potential treatment for hiv. Drug Metab Dispos. 2005;33(4):587.

268. De La Torre R, Farre M, Ortuno J, Mas M, Brenneisen R, Roset P, et al. Non‐linear pharmacokinetics of mdma (‘ecstasy’) in humans. Br J Clin Pharmacol. 2000;49(2):104-9.

269. de la Torre R, Farré M, Roset P, Pizarro N, Abanades S, Segura M, et al. Human pharmacology of mdma: Pharmacokinetics, metabolism, and disposition. Ther Drug Monit. 2004;26(2):137.

270. De Letter E, De Paepe P, Clauwaert K, Belpaire F, Lambert W, Van Bocxlaer J, et al. Is vitreous humour useful for the interpretation of 3, 4-methylenedioxymethamphetamine (mdma) blood levels? Experimental approach with rabbits. Int J Legal Med. 2000;114(1-2):29.

271. Johansson LC, Andersson M, Fager G, Gustafsson D, Eriksson UG. No influence of ethnic origin on the pharmacokinetics and pharmacodynamics of melagatran following oral administration of ximelagatran, a novel oral direct thrombin inhibitor, to healthy male volunteers. Clin Pharmacokinet. 2003;42(5):475-84.

272. Eriksson UG, Johansson S, Attman P-O, Mulec H, Frison L, Vager G, et al. Influence of severe renal impairment on the pharmacokinetics and pharmacodynamics of oral ximelagatran and subcutaneous melagatran. Clin Pharmacokinet. 2003;42(8):743-53.

273. Eriksson UG, Bredberg U, Hoffmann K-J, Thuresson A, Gabrielsson M, Ericsson H, et al. Absorption, distribution, metabolism, and excretion of ximelagatran, an oral direct thrombin inhibitor, in rats, dogs, and humans. Drug Metab Dispos. 2003;31(3):294-305.

274. Eriksson UG, Bredberg U, Gislén K, Johansson LC, Frison L, Ahnoff M, et al. Pharmacokinetics and pharmacodynamics of ximelagatran, a novel oral direct thrombin inhibitor, in young healthy male subjects. Eur J Clin Pharmacol. 2003;59(1):35-43.

275. Freudenthaler S, Meineke I, Schreeb K, Boakye E, Gundert-Remy U, Gleiter C. Influence of urine pH and urinary flow on the renal excretion of memantine. Br J Clin Pharmacol. 1998;46:541-6.

276. Jarvis B, Figgitt D. Memantine. Drug Aging. 2003;20(6):465.

277. Kornhuber J, Quack G. Cerebrospinal fluid and serum concentrations of the n-methyl-d-aspartate (nmda) receptor antagonist memantine in man. Neurosci Lett. 1995;195(2):137.

278. Periclou A, Ventura D, Rao N, Abramowitz W. Pharmacokinetic study of memantine in healthy and renally impaired subjects. Clin Pharmacol Ther. 2006;79(1):134-43.

279. Cutler M, Urquhart B, Velenosi T, Schwabedissen H, Dresser G, Leake B, et al. In vitro and in vivo assessment of renal drug transporters in the disposition of mesna and dimesna. J Clin Pharmacol. 2012;52(4):530-42.

280. James C, Mant T, Rogers H. Pharmacokinetics of intravenous and oral sodium 2‐mercaptoethane sulphonate (mesna) in normal subjects. Br J Clin Pharmacol. 1987;23(5):561-8.

281. Shaw I, Graham M. Mesna--a short review. Cancer Treat Rev. 1987;14(2):67.

282. Pentikäinen P, Neuvonen P, Penttilä A. Pharmacokinetics of metformin after intravenous and oral administration to man. Eur J Clin Pharmacol. 1979;16(3):195-202.

283. Pentikäinen P, Neuvonen P, Penttilä A. Pharmacokinetics of metformin after intravenous and oral administration to man. Eur J Clin Pharmacol. 1979;16(3):195.

284. Sambol NC, Chiang J, O'Conner M, Liu CY, Lin ET, Goodman AM, et al. Pharmacokinetics and pharmacodynamics of metformin in healthy subjects and patients with noninsulin‐dependent diabetes mellitus. J Clin Pharmacol. 1996;36(11):1012-21.

285. Tucker G, Casey C, Phillips P, Connor H, Ward J, Woods H. Metformin kinetics in healthy subjects and in patients with diabetes mellitus. Br J Clin Pharmacol. 1981;12(2):235-46.

286. Tzvetkov M, Vormfelde S, Balen D, Meineke I, Schmidt T, Sehrt D, et al. The effects of genetic polymorphisms in the organic cation transporters OCT1, OCT2, and OCT3 on the renal clearance of metformin. Clin Pharmacol Ther. 2009;86(3):299-306.

287. Abramson F. Methadone plasma protein binding: Alterations in cancer and displacement from alpha 1-acid glycoprotein. Clin Pharmacol Ther. 1982;32(5):652.

288. Foster DJ, Somogyi AA, Dyer KR, White JM, Bochner F. Steady-state pharmacokinetics of (r)-and (s)-methadone in methadone maintenance patients. Br J Clin Pharmacol. 2000;50(5):427-40.

289. Kharasch ED, Bedynek PS, Park S, Whittington D, Walker A, Hoffer C. Mechanism of ritonavir changes in methadone pharmacokinetics and pharmacodynamics: I. Evidence against CYP3A mediation of methadone clearance. Clin Pharmacol Ther. 2008;84(4):497-505.

290. Kharasch ED, Hoffer C, Whittington D, Sheffels P. Role of hepatic and intestinal cytochrome P450 3A and 2b6 in the metabolism, disposition, and miotic effects of methadone. Clin Pharmacol Ther. 2004;76(3):250-69.

291. Lugo R, Satterfield K, Kern S. Pharmacokinetics of methadone. J Pain Palliat Care Pharmacother. 2005;19(4):13.

292. Nilsson M-I, Widerlöv E, Meresaar U, Änggård E. Effect of urinary pH on the disposition of methadone in man. Eur J Clin Pharmacol. 1982;22(4):337-42.

293. Kirchheiner J, Heesch C, Bauer S, Meisel C, Seringer A, Goldammer M, et al. Impact of the ultrarapid metabolizer genotype of cytochrome P450 2d6 on metoprolol pharmacokinetics and pharmacodynamics. Clin Pharmacol Ther. 2004;76(4):302-12.

294. Jack DB, Kendall MJ, Dean S, Laugher SJ, Zaman R, Tenneson ME. The effect of hydralazine on the pharmacokinetics of three different beta adrenoceptor antagonists: Metoprolol, nadolol, and acebutolol. Biopharm Drug Dispos. 1982;3(1):47-54.

295. Regårdh CG, Borg KO, Johansson R, Johnsson G, Palmer L. Pharmacokinetic studies on the selectiveβ 1-receptor antagonist metoprolol in man. J Pharmacokinet Biopharm. 1974;2(4):347-64.

296. Loft S, Døssing M, Poulsen H, Sonne J, Olesen K-L, Simonsen K, et al. Influence of dose and route of administration on disposition of metronidazole and its major metabolites. Eur J Clin Pharmacol. 1986;30(4):467-73.

297. Loft S, Sonne J, Poulsen H, Petersen K, Jørgensen B, Døssing M. Inhibition and induction of metronidazole and antipyrine metabolism. Eur J Clin Pharmacol. 1987;32(1):35-41.

298. Ralph ED, Clarke JT, Libke RD, Luthy RP, Kirby WM. Pharmacokinetics of metronidazole as determined by bioassay. Antimicrob Agents Chemother. 1974;6(6):691-6.

299. Sanvordeker D, Chien Y, Lin T, Lambert H. Binding of metronidazole and its derivatives to plasma proteins: An assessment of drug binding phenomenon. J Pharm Sci. 1975;64(11):1797.

300. Grech-Belanger O, Turgeon J, Gilbert M. Stereoselective disposition of mexiletine in man. Br J Clin Pharmacol. 1986;21(5):481-7.

301. Kwok D, Kerr C, McErlane K. Pharmacokinetics of mexiletine enantiomers in healthy human subjects. A study of the in vivo serum protein binding, salivary excretion and red blood cell distribution of the enantiomers. Xenobiotica. 1995;25(10):1127.

302. Mitchell B, Clements J, Pottage A, Prescott L. Mexiletine disposition: Individual variation in response to urine acidification and alkalinisation. Br J Clin Pharmacol. 1983;16(3):281-4.

303. Pentikäinen P, Koivula I, Hiltunen H. Effect of rifampicin treatment on the kinetics of mexiletine. Eur J Clin Pharmacol. 1982;23(3):261-6.

304. Talbot R, Nimmo J, Julian D, Clark R, Neilson J, Prescott L. Treatment of ventricular arrhythmias with mexiletine (kö 1173). Lancet. 1973;2(7826):399.

305. Härtter S, Dingemanse J, Baier D, Ziegler G, Hiemke C. The role of cytochrome P450 2d6 in the metabolism of moclobemide. Eur Neuropsychopharmacol. 1996;6(3):225.

306. Yu K-S, Yim D-S, Cho J-Y, Park SS, Park JY, Lee K-H, et al. Effect of omeprazole on the pharmacokinetics of moclobemide according to the genetic polymorphism of cyp2c19. Clin Pharmacol Ther. 2001;69(4):266-73.

307. Fromm MF, Eckhardt K, Li S, Schänzle G, Hofmann U, Mikus G, et al. Loss of analgesic effect of morphine due to coadministration of rifampin. Pain. 1997;72(1):261-7.

308. Glare P, Walsh T. Clinical pharmacokinetics of morphine. Ther Drug Monit. 1991;13(1):1.

309. Hasselström J, Säwe J. Morphine pharmacokinetics and metabolism in humans. Clin Pharmacokinet. 1993;24(4):344-54.

310. Milne R, Nation R, Somogyi A, Bochner F, Griggs W. The influence of renal function on the renal clearance of morphine and its glucuronide metabolites in intensive-care patients. Br J Clin Pharmacol. 1992;34(1):53.

311. Bolton WK, Scheld WM, Spyker DA, Overby TL, Sande M. Pharmacokinetics of moxalactam in subjects with various degrees of renal dysfunction. Antimicrob Agents Chemother. 1980;18(6):933-8.

312. DeSante KA, Israel KS, Brier GL, Wolny JD, Hatcher BL. Effect of probenecid on the pharmacokinetics of moxalactam. Antimicrob Agents Chemother. 1982;21(1):58-61.

313. Kemmerich B, Lode H, Belmega G, Jendroschek T, Borner K, Koeppe P. Comparative pharmacokinetics of cefoperazone, cefotaxime, and moxalactam. Antimicrob Agents Chemother. 1983;23(3):429-34.

314. Peterson L, Bean B, Fasching C, Korchik W, Gerding D. Pharmacokinetics, protein binding, and predicted extravascular distribution of moxalactam in normal and renal failure subjects. Antimicrob Agents Chemother. 1981;20(3):378.

315. Scheld W, Spyker D, Donowitz G, Bolton W, Sande M. Moxalactam and cefazolin: Comparative pharmacokinetics in normal subjects. Antimicrob Agents Chemother. 1981;19(4):613-9.

316. Srinivasan S, Fu K, Neu H. Pharmacokinetics of moxalactam and cefazolin compared in normal volunteers. Antimicrob Agents Chemother. 1981;19(2):302-5.

317. Standiford H, Drusano G, Bustamante C, Rivera G, Forrest A, Tatem B, et al. Imipenem coadministered with cilastatin compared with moxalactam: Integration of serum pharmacokinetics and microbiologic activity following single-dose administration to normal volunteers. Antimicrob Agents Chemother. 1986;29(3):412.

318. Stass H, Dalhoff A, Kubitza D, Schühly U. Pharmacokinetics, safety, and tolerability of ascending single doses of moxifloxacin, a new 8-methoxy quinolone, administered to healthy subjects. Antimicrob Agents Chemother. 1998;42(8):2060-5.

319. Stass H, Kubitza D. Pharmacokinetics and elimination of moxifloxacin after oral and intravenous administration in man. J Antimicrob Chemother. 1999;43(suppl 2):83-90.

320. Stass H, Kubitza D, Halabi A, Delesen H. Pharmacokinetics of moxifloxacin, a novel 8‐methoxy‐quinolone, in patients with renal dysfunction. Br J Clin Pharmacol. 2002;53(3):232-7.

321. Stass H, Kubitza D, Möller JG, Delesen H. Influence of activated charcoal on the pharmacokinetics of moxifloxacin following intravenous and oral administration of a 400 mg single dose to healthy males. Br J Clin Pharmacol. 2005;59(5):536-41.

322. Zeitlinger M, Sauermann R, Fille M, Hausdorfer J, Leitner I, Müller M. Plasma protein binding of fluoroquinolones affects antimicrobial activity. J Antimicrob Chemoth. 2008;61(3):561.

323. Waller E, Sharanevych M, Yakatan G. The effect of probenecid on nafcillin disposition. J Clin Pharmacol. 1982;22(10):482.

324. Lode H, Höffken G, Olschewski P, Sievers B, Kirch A, Borner K, et al. Pharmacokinetics of ofloxacin after parenteral and oral administration. Antimicrob Agents Chemother. 1987;31(9):1338-42.

325. Yuk J, Nightingale C, Quintiliani R, Sweeney K. Bioavailability and pharmacokinetics of ofloxacin in healthy volunteers. Antimicrob Agents Chemother. 1991;35(2):384.

326. Schwocho L, Masonson H. Pharmacokinetics of cs-866, a new angiotensin II receptor blocker, in healthy subjects. J Clin Pharmacol. 2001;41(5):515.

327. Gardner S, Franks A. Olmesartan medoxomil: The seventh angiotensin receptor antagonist. Ann Pharmacother. 2003;37(1):99.

328. Chrysant S, Chrysant G. Antihypertensive efficacy of olmesartan medoxomil alone and in combination with hydrochlorothiazide. Expert Opin Pharmacother. 2004;5(3):657.

329. He G, Massarella J, Ward P. Clinical pharmacokinetics of the prodrug oseltamivir and its active metabolite ro 64-0802. Clin Pharmacokinet. 1999;37(6):471.

330. Brennan B, Davies B, Cirrincione-Dall G, Morcos P, Beryozkina A, Chappey C, et al. Safety, tolerability, and pharmacokinetics of intravenous oseltamivir: Single-and multiple-dose phase i studies with healthy volunteers. Antimicrob Agents Chemother. 2012;56(9):4729.

331. Laethem M, Lefebvre R, Belpaire F, Vanhoe H, Bogaert M. Stereoselective pharmacokinetics of oxprenolol and its glucuronides in humans. Clin Pharmacol Ther. 1995;57(4):419.

332. Green R, Brown J, Calvert R. The disposition of four tetracyclines in normal subjects. Eur J Clin Pharmacol. 1976;10(3):245-50.

333. Kunin CM, Dornbush A, Finland M. Distribution and excretion of four tetracycline analogues in normal young men. J Clin Invest. 1959;38(11):1950.

334. Barre J, Houin G, Tillement J. Dose-dependent pharmacokinetic study of pefloxacin, a new antibacterial agent, in humans. J Pharm Sci. 1984;73(10):1379.

335. Montay G, Goueffon Y, Roquet F. Absorption, distribution, metabolic fate, and elimination of pefloxacin mesylate in mice, rats, dogs, monkeys, and humans. Antimicrob Agents Chemother. 1984;25(4):463.

336. Frydman A, Le Roux Y, Lefebvre M, Djebbar F, Fourtilllan J, Gaillot J. Pharmacokinetics of pefloxacin after repeated intravenous and oral administration (400 mg bid) in young healthy volunteers. J Antimicrob Chemother. 1986;17(suppl B):65-79.

337. Pue M, Pratt S, Fairless A, Fowles S, Laroche J, Georgiou P, et al. Linear pharmacokinetics of penciclovir following administration of single oral doses of famciclovir 125, 250, 500 and 750 mg to healthy volunteers. J Antimicrob Chemoth. 1994;33(1):119.

338. Vinh D, Aoki F. Famciclovir for the treatment of recurrent genital herpes: A clinical and pharmacological perspective. Expert Opin Pharmacother. 2006;7(16):2271.

339. Takabatake T, Ohta H, Yamamoto Y, Ishida Y, Hara H, Ushiogi Y, et al. Pharmacokinetics of sun 1165, a new antiarrhythmic agent, in renal dysfunction. Eur J Clin Pharmacol. 1991;40(4):411.

340. Shiga T, Hashiguchi M, Urae A, Kasanuki H, Rikihisa T. Effect of cimetidine and probenecid on pilsicainide renal clearance in humans. Clin Pharmacol Ther. 2000;67(3):222.

341. Kim B, Kim J, Lim K, Kim J, Kim K, Hong J, et al. An open-label, single-dose, parallel-group, dose-increasing study comparing the pharmacokinetics and tolerability of pilsicainide hydrochloride in healthy korean and japanese male subjects. Clin Ther. 2009;31(3):609.

342. Taylor E, Turner P. The distribution of propranolol, pindolol and atenolol between human erythrocytes and plasma. Br J Clin Pharmacol. 1981;12(4):543.

343. Hsyu P-H, Giacomini KM. Stereoselective renal clearance of pindolol in humans. J Clin Invest. 1985;76(5):1720.

344. Somogyi A, Bochner F, Sallustio B. Stereoselective inhibition of pindolol renal clearance by cimetidine in humans. Clin Pharmacol Ther. 1992;51(4):379.

345. Ujhelyi M, Bottorff M, Schur M, Roll K, Zhang H, Stewart J, et al. Aging effects on the organic base transporter and stereoselective renal clearance. Clin Pharmacol Ther. 1997;62(2):117.

346. Aronoff G, Sloan R, Brier M, Luft F. The effect of piperacillin dose on elimination kinetics in renal impairment. Eur J Clin Pharmacol. 1983;24(4):543.

347. Kyrklund C, Backman J, Neuvonen M, Neuvonen P. Gemfibrozil increases plasma pravastatin concentrations and reduces pravastatin renal clearance. Clin Pharmacol Ther. 2003;73(6):538.

348. Halstenson C, Triscari J, DeVault A, Shapiro B, Keane W, Pan H. Single-dose pharmacokinetics of pravastatin and metabolites in patients with renal impairment. J Clin Pharmacol. 1992;32(2):124.

349. Singhvi S, Pan H, Morrison R, Willard D. Disposition of pravastatin sodium, a tissue-selective hmg-coa reductase inhibitor, in healthy subjects. Br J Clin Pharmacol. 1990;29(2):239.

350. Powell L, Axelsen E. Corticosteroids in liver disease: Studies on the biological conversion of prednisone to prednisolone and plasma protein binding. Gut. 1972;13(9):690.

351. Ağabeyoğlu I, Bergstrom R, Gillespie W, Wagner J, Kay D. Plasma protein binding of prednisolone in normal volunteers and arthritic patients. Eur J Clin Pharmacol. 1979;16(6):399.

352. Rose J, Yurchak A, Jusko W. Dose dependent pharmacokinetics of prednisone and prednisolone in man. J Pharmacokinet Biopharm. 1981;9(4):389.

353. Garg V, Blum R, Wilner K, Jusko W. Effect of the anti-inflammatory agent tenidap on the pharmacokinetics and pharmacodynamics of prednisolone. J Clin Pharmacol. 1992;32(3):222.

354. Boudinot F, Jusko W. Plasma protein binding interaction of prednisone and prednisolone. J Steroid Biochem. 1984;21(3):337.

355. Emanuelsson B, Beermann B, Paalzow L. Non-linear elimination and protein binding of probenecid. Eur J Clin Pharmacol. 1987;32(4):395.

356. Vree T, Van Ewijk-Beneken KE, Wuis E, Hekster Y. Capacity-limited renal glucuronidation of probenecid by humans. A pilot vmax-finding study. Pharm Weekbl Sci. 1992;14(5):325.

357. Vree T, Van Ewijk-Beneken KE, Wuis E, Hekster Y, Broekman M. Interindividual variation in the capacity-limited renal glucuronidation of probenecid by humans. Pharm World Sci. 1993;15(5):197.

358. Rodvold K, Paloucek F, Jung D, Gallastegui J. Interaction of steady-state procainamide with h2-receptor antagonists cimetidine and ranitidine. Ther Drug Monit. 1987;9(4):378.

359. Rocci Jr M, Kosoglou T, Ferguson R, Vlasses P. Ranitidine-induced changes in the renal and hepatic clearances of procainamide are correlated. J Pharmacol Exp Ther. 1989;248(3):923.

360. Lam Y, Boyd R, Chin S, Chang D, Giacomini K. Effect of probenecid on the pharmacokinetics and pharmacodynamics of procainamide. J Clin Pharmacol. 1991;31(5):429.

361. Sarre S, Van Belle K, Smolders I, Krieken G, Michotte Y. The use of microdialysis for the determination of plasma protein binding of drugs. J Pharm Biomed Anal. 1992;10(10-12):735.

362. Martin D, Shen J, Griener J, Raasch R, Patterson J, Cascio W. Effects of ofloxacin on the pharmacokinetics and pharmacodynamics of procainamide. J Clin Pharmacol. 1996;36(1):85.

363. Bauer LA, Black DJ, Lill JS, Garrison J, Raisys VA, Hooton TM. Levofloxacin and ciprofloxacin decrease procainamide and n-acetylprocainamide renal clearances. Antimicrob Agents Chemother. 2005;49(4):1649.

364. DiGregorio G, Ruch E. Human whole blood and parotid saliva concentrations of oral and intramuscular promethazine. J Pharm Sci. 1980;69(12):1457.

365. Taylor G, Houston J, Shaffer J, Mawer G. Pharmacokinetics of promethazine and its sulphoxide metabolite after intravenous and oral administration to man. Br J Clin Pharmacol. 1983;15(3):287.

366. Chan G, Axelson J, Price J, McErlane K, Kerr C. In vitro protein binding of propafenone in normal and uraemic human sera. Eur J Clin Pharmacol. 1989;36(5):495.

367. Vozeh S, Haefeli W, Ha H, Vlcek J, Follath F. Nonlinear kinetics of propafenone metabolites in healthy man. Eur J Clin Pharmacol. 1990;38(5):509.

368. Dilger K, Greiner B, Fromm M, Hofmann U, Kroemer H, Eichelbaum M. Consequences of rifampicin treatment on propafenone disposition in extensive and poor metabolizers of CYP2D6. Pharmacogenetics. 1999;9(5):551.

369. Chen X, Zhong D, Blume H. Stereoselective pharmacokinetics of propafenone and its major metabolites in healthy chinese volunteers. Eur J Pharm Sci. 2000;10(1):11.

370. Komura H, Iwaki M. Nonlinear pharmacokinetics of propafenone in rats and humans: Application of a substrate depletion assay using hepatocytes for assessment of nonlinearity. Drug Metab Dispos. 2005;33(6):726.

371. Giles H, Roberts E, Orrego H, Sellers E. Disposition of intravenous propylthiouracil. J Clin Pharmacol. 1981;21(11-12 Pt 1):466.

372. Kampmann J, Hansen JM. Serum protein binding of propylthiouracil. Br J Clin Pharmacol. 1983;16(5):549.

373. Lacroix C, Guyonnaud C, Chaou M, Duwoos H, Lafont O. Interaction between allopurinol and pyrazinamide. Eur Respir J. 1988;1(9):807.

374. Lacroix C, Hoang T, Nouveau J, Guyonnaud C, Laine G, Duwoos H, et al. Pharmacokinetics of pyrazinamide and its metabolites in healthy subjects. Eur J Clin Pharmacol. 1989;36(4):395.

375. Woo J, Cheung W, Chan R, Chan H, Cheng A, Chan K. In vitro protein binding characteristics of isoniazid, rifampicin, and pyrazinamide to whole plasma, albumin, and alpha-1-acid glycoprotein. Clin Biochem. 1996;29(2):175.

376. Fremstad D, Bergerud K, Haffner J, Lunde P. Increased plasma binding of quinidine after surgery: A preliminary report. Eur J Clin Pharmacol. 1976;10(6):441.

377. Greenblatt D, Pfeifer H, Ochs H, Franke K, MacLaughlin D, Smith T, et al. Pharmacokinetics of quinidine in humans after intravenous, intramuscular and oral administration. J Pharmacol Exp Ther. 1977;202(2):365.

378. Fremstad D, Nilsen O, Storstein L, Amlie J, Jacobsen S. Pharmacokinetics of quinidine related to plasma protein binding in man. Eur J Clin Pharmacol. 1979;15(3):187.

379. Woo E, Greenblatt D. Pharmacokinetic and clinical implications of quinidine protein binding. J Pharm Sci. 1979;68(4):466.

380. Rakhit A, Holford N, Guentert T, Maloney K, Riegelman S. Pharmacokinetics of quinidine and three of its metabolites in man. J Pharmacokinet Biopharm. 1984;12(1):1.

381. Kaukonen K, Olkkola K, Neuvonen P. Itraconazole increases plasma concentrations of quinidine. Clin Pharmacol Ther. 1997;62(5):510.

382. Damkier P, Hansen L, Brøsen K. Effect of fluvoxamine on the pharmacokinetics of quinidine. Eur J Clin Pharmacol. 1999;55(6):451.

383. Damkier P, Hansen LL, Brøsen K. Effect of diclofenac, disulfiram, itraconazole, grapefruit juice and erythromycin on the pharmacokinetics of quinidine. Br J Clin Pharmacol. 1999;48(6):829.

384. Iwamoto M, Wenning L, Petry A, Laethem M, De Smet M, Kost J, et al. Safety, tolerability, and pharmacokinetics of raltegravir after single and multiple doses in healthy subjects. Clin Pharmacol Ther. 2008;83(2):293.

385. Laufer R, Paz O, Di Marco A, Bonelli F, Monteagudo E, Summa V, et al. Quantitative prediction of human clearance guiding the development of raltegravir (mk-0518, isentress) and related hiv integrase inhibitors. Drug Metab Dispos. 2009;37(4):873.

386. Widerlöv E, Termander B, Nilsson M. Effect of urinary pH on the plasma and urinary kinetics of remoxipride in man. Eur J Clin Pharmacol. 1989;37(4):359.

387. Movin-Osswald G, Hammarlund-Udenaes M. Remoxipride: Pharmacokinetics and effect on plasma prolactin. Br J Clin Pharmacol. 1991;32(3):355.

388. Movin-Osswald G, Boelaert J, Hammarlund-Udenaes M, Nilsson L. The pharmacokinetics of remoxipride and metabolites in patients with various degrees of renal function. Br J Clin Pharmacol. 1993;35(6):615.

389. Yisak W, Farde L, von Bahr C, Nilsson L, Fredriksson G, Ogenstad S. Interaction study between remoxipride and biperiden. Psychopharmacology. 1993;111(1):27.

390. N'soukpoe-Kossi C, St-Louis C, Beauregard M, Subirade M, Carpentier R, Hotchandani S, et al. Resveratrol binding to human serum albumin. J Biomol Struct Dyn. 2006;24(3):277.

391. Boocock D, Faust G, Patel K, Schinas A, Brown V, Ducharme M, et al. Phase i dose escalation pharmacokinetic study in healthy volunteers of resveratrol, a potential cancer chemopreventive agent. Cancer Epidemiol Biomarkers Prev. 2007;16(6):1246.

392. Burkon A, Somoza V. Quantification of free and protein-bound trans-resveratrol metabolites and identification of trans-resveratrol-c/o-conjugated diglucuronides-two novel resveratrol metabolites in human plasma. Mol Nutr Food Res. 2008;52(5):549.

393. Kantesaria B, Glue P. Exploring the influence of renal dysfunction on the pharmaco-kinetics of ribavirin after oral and intravenous dosing. Drug Discov Ther. 2014;8(2):89.

394. Glue P, Schenker S, Gupta S, Clement RP, Zambas D, Salfi M. The single dose pharmacokinetics of ribavirin in subjects with chronic liver disease. Br J Clin Pharmacol. 2000;49(5):417.

395. Preston SL, Drusano GL, Glue P, Nash J, Gupta S, McNamara P. Pharmacokinetics and absolute bioavailability of ribavirin in healthy volunteers as determined by stable-isotope methodology. Antimicrob Agents Chemother. 1999;43(10):2451.

396. Blaschke T, Skinner M. The clinical pharmacokinetics of rifabutin. Clin Infect Dis. 1996;22:S15.

397. Polk RE, Brophy DF, Israel DS, Patron R, Sadler BM, Chittick GE, et al. Pharmacokinetic interaction between amprenavir and rifabutin or rifampin in healthy males. Antimicrob Agents Chemother. 2001;45(2):502.

398. Boman G, Ringberger V. Binding of rifampicin by human plasma proteins. Eur J Clin Pharmacol. 1974;7(5):369.

399. Peloquin C, Namdar R, Singleton M, Nix D. Pharmacokinetics of rifampin under fasting conditions, with food, and with antacids. Chest. 1999;115(1):12.

400. Huang M, Van Peer A, Woestenborghs R, De Coster R, Heykants J, Jansen A, et al. Pharmacokinetics of the novel antipsychotic agent risperidone and the prolactin response in healthy subjects. Clin Pharmacol Ther. 1993;54(3):257.

401. Mannens G, Huang M, Meuldermans W, Hendrickx J, Woestenborghs R, Heykants J. Absorption, metabolism, and excretion of risperidone in humans. Drug Metab Dispos. 1993;21(6):1134.

402. Snoeck E, Van Peer A, Sack M, Horton M, Mannens G, Woestenborghs R, et al. Influence of age, renal and liver impairment on the pharmacokinetics of risperidone in man. Psychopharmacology. 1995;122(3):223.

403. Weinz C, Buetehorn U, Daehler H, Kohlsdorfer C, Pleiss U, Sandmann S, et al. Pharmacokinetics of bay 59-7939--an oral, direct factor xa inhibitor--in rats and dogs. Xenobiotica. 2005;35(9):891.

404. Kubitza D, Becka M, Mueck W, Halabi A, Maatouk H, Klause N, et al. Effects of renal impairment on the pharmacokinetics, pharmacodynamics and safety of rivaroxaban, an oral, direct factor xa inhibitor. Br J Clin Pharmacol. 2010;70(5):703.

405. Perzborn E, Roehrig S, Straub A, Kubitza D, Mueck W, Laux V. Rivaroxaban: A new oral factor xa inhibitor. Atertio Thromb Vasc Biol. 2010;30(3):376.

406. Lee A, Fagan D, Lamont M, Tucker G, Halldin M, Scott D. Disposition kinetics of ropivacaine in humans. Anesth Analg. 1989;69(6):736.

407. Jokinen M, Ahonen J, Neuvonen P, Olkkola K. The effect of erythromycin, fluvoxamine, and their combination on the pharmacokinetics of ropivacaine. Anesth Analg. 2000;91(5):1207.

408. Jokinen M, Olkkola K, Ahonen J, Neuvonen P. Effect of rifampin and tobacco smoking on the pharmacokinetics of ropivacaine. Clin Pharmacol Ther. 2001;70(4):344.

409. Pere P, Salonen M, Jokinen M, Rosenberg P, Neuvonen P, Haasio J. Pharmacokinetics of ropivacaine in uremic and nonuremic patients after axillary brachial plexus block. Anesth Analg. 2003;96(2):563.

410. Pere P, Ekstrand A, Salonen M, Honkanen E, Sjövall J, Henriksson J, et al. Pharmacokinetics of ropivacaine in patients with chronic renal failure. Br J Anaesth. 2011;106(4):512.

411. White C. A review of the pharmacologic and pharmacokinetic aspects of rosuvastatin. J Clin Pharmacol. 2002;42(9):963.

412. Martin P, Warwick M, Dane A, Brindley C, Short T. Absolute oral bioavailability of rosuvastatin in healthy white adult male volunteers. Clin Ther. 2003;25(10):2553.

413. Imbimbo B, Broccali G, Cesana M, Crema F, Attardo-Parrinello G. Inter-and intrasubject variabilities in the pharmacokinetics of rufloxacin after single oral administration to healthy volunteers. Antimicrob Agents Chemother. 1991;35(2):390.

414. Kisicki J, Griess R, Ott C, Cohen G, McCormack R, Troetel W, et al. Multiple-dose pharmacokinetics and safety of rufloxacin in normal volunteers. Antimicrob Agents Chemother. 1992;36(6):1296.

415. Segre G, Cerretani D, Moltoni L, Urso R. Pharmacokinetics of rufloxacin in healthy volunteers. Eur J Clin Pharmacol. 1992;42(1):101.

416. Ward JK, Dow J, Dallow N, Eynott P, Milleri S, Ventresca GP. Enantiomeric disposition of inhaled, intravenous and oral racemic-salbutamol in man—no evidence of enantioselective lung metabolism. Br J Clin Pharmacol. 2000;49(1):15.

417. Morgan D, Paull J, Richmond B, Wilson-Evered E, Ziccone S. Pharmacokinetics of intravenous and oral salbutamol and its sulphate conjugate. Br J Clin Pharmacol. 1986;22(5):587.

418. Shi J, Ripley E, Gehr T, Sica D, Dandekar K, Hinderling P. Pharmacokinetics of sematilide in renal failure. J Clin Pharmacol. 1996;36(2):131.

419. Hinderling P, Dilea C, Koziol T, Millington G. Comparative kinetics of sematilide in four species. Drug Metab Dispos. 1993;21(4):662.

420. Herman G, Stevens C, Van Dyck K, Bergman A, Yi B, De Smet M, et al. Pharmacokinetics and pharmacodynamics of sitagliptin, an inhibitor of dipeptidyl peptidase IV, in healthy subjects: Results from two randomized, double-blind, placebo-controlled studies with single oral doses. Clin Pharmacol Ther. 2005;78(6):675.

421. Bergman A, Stevens C, Zhou Y, Yi B, Laethem M, De Smet M, et al. Pharmacokinetic and pharmacodynamic properties of multiple oral doses of sitagliptin, a dipeptidyl peptidase-IV inhibitor: A double-blind, randomized, placebo-controlled study in healthy male volunteers. Clin Ther. 2006;28(1):55.

422. Bergman A, Cote J, Yi B, Marbury T, Swan S, Smith W, et al. Effect of renal insufficiency on the pharmacokinetics of sitagliptin, a dipeptidyl peptidase-4 inhibitor. Diabetes Care. 2007;30(7):1862.

423. Herman G, Stein P, Thornberry N, Wagner J. Dipeptidyl peptidase-4 inhibitors for the treatment of type 2 diabetes: Focus on sitagliptin. Clin Pharmacol Ther. 2007;81(5):761.

424. Montay G, Bruno R, Vergniol J, Ebmeier M, Le Roux Y, Guimart C, et al. Pharmacokinetics of sparfloxacin in humans after single oral administration at doses of 200, 400, 600, and 800 mg. J Clin Pharmacol. 1994;34(11):1071.

425. Ritz M, Lode H, Fassbender M, Borner K, Koeppe P, Nord C. Multiple-dose pharmacokinetics of sparfloxacin and its influence on fecal flora. Antimicrob Agents Chemother. 1994;38(3):455.

426. Kamberi M, Kotegawa T, Tsutsumi K, Nakamura K, Nakano S. Sparfloxacin pharmacokinetics in healthy volunteers: The influence of acidification and alkalinization. Eur J Clin Pharmacol. 1998;54(8):633.

427. Dorr M, Johnson R, Jensen B, Magner D, Marbury T, Talbot G. Pharmacokinetics of sparfloxacin in patients with renal impairment. Clin Ther. 1999;21(7):1202.

428. Sharpstone P. The renal handling of trimethoprim and sulphamethoxazole in man. Postgrad Med J. 1969;45:Suppl: 38.

429. Vree T, Hekster Y, Baars A, Damsma J, Kleijin E. Determination of trimethoprim and sulfamethoxazole (co-trimoxazole) in body fluids of man by means of high-performance liquid chromatography. J Chromatogr. 1978;146(1):103.

430. Stevens R, Laizure S, Sanders P, Stein D. Multiple-dose pharmacokinetics of 12 milligrams of trimethoprim and 60 milligrams of sulfamethoxazole per kilogram of body weight per day in healthy volunteers. Antimicrob Agents Chemother. 1993;37(3):448.

431. Vree TB, van der Ven AJ, Koopmans PP, Kolmer EWvE-B, Verwey-van Wissen CP. Pharmacokinetics of sulfamethoxazole with its hydroxy metabolites and n4-acetyl-, n1-glucuronide conjugates in healthy human volunteers. Clin Drug Invest. 1995;9(1):43-53.

432. Hu P, Jiang J, Wang H, Pietropaolo K, Chao G, Brown N, et al. Single-dose and multiple-dose pharmacokinetics and safety of telbivudine after oral administration in healthy chinese subjects. J Clin Pharmacol. 2006;46(9):999.

433. Zhou X-J, Fielman BA, Lloyd DM, Chao GC, Brown NA. Pharmacokinetics of telbivudine in healthy subjects and absence of drug interaction with lamivudine or adefovir dipivoxil. Antimicrob Agents Chemother. 2006;50(7):2309.

434. Zhou X-J, Swan S, Smith WB, Marbury TC, Dubuc-Patrick G, Chao GC, et al. Pharmacokinetics of telbivudine in subjects with various degrees of renal impairment. Antimicrob Agents Chemother. 2007;51(12):4231.

435. Granneman G, Carpentier P, Morrison P, Pernet A. Pharmacokinetics of temafloxacin in humans after single oral doses. Antimicrob Agents Chemother. 1991;35(3):436.

436. Granneman G, Carpentier P, Morrison P, Pernet A. Pharmacokinetics of temafloxacin in humans after multiple oral doses. Antimicrob Agents Chemother. 1992;36(2):378.

437. Slocombe B, Basker M, Bentley P, Clayton J, Cole M, Comber K, et al. Brl 17421, a novel beta-lactam antibiotic, highly resistant to beta-lactamases, giving high and prolonged serum levels in humans. Antimicrob Agents Chemother. 1981;20(1):38.

438. Overbosch D, van Gulpen C, Mattie H. Renal clearance of temocillin in volunteers. Drugs. 1985;29:128.

439. Kearney B, Flaherty J, Shah J. Tenofovir disoproxil fumarate: Clinical pharmacology and pharmacokinetics. Clin Pharmacokinet. 2004;43(9):595.

440. Goicoechea M, Best B. Efavirenz/emtricitabine/tenofovir disoproxil fumarate fixed-dose combination: First-line therapy for all? Expert Opin Pharmacother. 2007;8(3):371.

441. Jaffe JM, Colaizzi JL, Poust RI, McDonald Jr RH. Effect of altered urinaryph on tetracycline and doxycycline excretion in humans. J Pharmacokinet Biopharm. 1973;1(4):267-82.

442. Hallén B, Guilbaud O, Strömberg S, Lindeke B. Single-dose pharmacokinetics of terodiline, including a stable isotope technique for improvement of statistical evaluations. Biopharm Drug Dispos. 1988;9(3):229.

443. Hallén B, Gabrielsson J, Nyambati S, Johansson A, Larsson E, Guilbaud O. Concomitant single-dose and multiple-dose pharmacokinetics of terodiline in man, with a note on its enantiomers and major metabolites. Pharmacol Toxicol. 1995;76(3):171.

444. Webster R, Allan G, Anto-Awuakye K, Harrison A, Kidd T, Leishman D, et al. Pharmacokinetic/pharmacodynamic assessment of the effects of e4031, cisapride, terfenadine and terodiline on monophasic action potential duration in dog. Xenobiotica. 2001;31(8-9):633.

445. Buss D, Leopold D, Smith A, Routledge P. Determinants of the plasma protein binding of theophylline in health. Br J Clin Pharmacol. 1983;15(4):399.

446. Birkett D, Dahlqvist R, Miners J, Lelo A, Billing B. Comparison of theophylline and theobromine metabolism in man. Drug Metab Dispos. 1985;13(6):725.

447. Vanholder R, Van Landschoot N, De Smet R, Schoots A, Ringoir S. Drug protein binding in chronic renal failure: Evaluation of nine drugs. Kidney Int. 1988;33(5):996.

448. Liu L, Pan X, Liu H-y, Liu X-d, Yang H-w, Xie L, et al. Modulation of pharmacokinetics of theophylline by antofloxacin, a novel 8-amino-fluoroquinolone, in humans. Acta Pharmacol Sin. 2011;32(10):1285.

449. Fourtillan J, Courtois P, Lefebvre M, Girault J. Pharmacokinetics of oral timolol studied by mass fragmentography. Eur J Clin Pharmacol. 1981;19(3):193.

450. Mäntylä R, Männistö P, Nykänen S, Koponen A, Lamminsivu U. Pharmacokinetic interactions of timolol with vasodilating drugs, food and phenobarbitone in healthy human volunteers. Eur J Clin Pharmacol. 1983;24(2):227.

451. Wood S, John B, Chasseaud L, Brodie R, Baker J, Faulkner J, et al. Pharmacokinetics and metabolism of 14c-tinidazole in humans. J Antimicrob Chemoth. 1986;17(6):801.

452. Wood B, Faulkner J, Monro A. The pharmacokinetics, metabolism and tissue distribution of tinidazole. J Antimicrob Chemoth. 1982;10:43.

453. Chaikin P, Alton K, Sampson C, Weintraub H. Pharmacokinetics of tinidazole in male and female subjects. J Clin Pharmacol. 1982;22(11-12):562.

454. Wagstaff A, Bryson H. Tizanidine. A review of its pharmacology, clinical efficacy and tolerability in the management of spasticity associated with cerebral and spinal disorders. Drugs. 1997;53(3):435.

455. Shellenberger M, Groves L, Shah J, Novack G. A controlled pharmacokinetic evaluation of tizanidine and baclofen at steady state. Drug Metab Dispos. 1999;27(2):201.

456. Granfors M, Backman J, Laitila J, Neuvonen P. Oral contraceptives containing ethinyl estradiol and gestodene markedly increase plasma concentrations and effects of tizanidine by inhibiting cytochrome P450 1a2. Clin Pharmacol Ther. 2005;78(4):400.

457. Lalka D, Meyer M, Duce B, Elvin A. Kinetics of the oral antiarrhythmic lidocaine congener, tocainide. Clin Pharmacol Ther. 1976;19(6):757.

458. Graffner C, Conradson T, Hofvendahl S, Rydén L. Tocainide kinetics after intravenous and oral administration in healthy subjects and in patients with acute myocardial infarction. Clin Pharmacol Ther. 1980;27(1):64.

459. Sedman A, Bloedow D, Gal J. Serum binding of tocainide and its enantiomers in human subjects. Res Commun Chem Pathol Pharmacol. 1982;38(1):165.

460. Braun J, Sörgel F, Engelmaier F, Gluth W, Gessler U. Pharmacokinetics of tocainide in patients with severe renal failure. Eur J Clin Pharmacol. 1985;28(6):665.

461. McErlane K, Axelson J, Vaughan R, Kerr C, Price J, Igwemezie L, et al. Stereoselective pharmacokinetics of tocainide in human uraemic patients and in healthy subjects. Eur J Clin Pharmacol. 1990;39(4):373.

462. Mallalieu NL, Lennon S, Guy T, Liu M, Luedin E, Davies BE. Lack of age and gender effects on single-dose pharmacokinetics of tomopenem (ro4908463/cs-023), a novel carbapenem. Br J Clin Pharmacol. 2009;67(4):469.

463. Mallalieu NL, Lennon S, Liu M, Kirkpatrick C, Robson R, Luedin E, et al. Effect of impaired renal function on the pharmacokinetics of tomopenem (ro4908463/cs-023), a novel carbapenem. Antimicrob Agents Chemother. 2008;52(7):2360.

464. Shibayama T, Matsushita Y, Hirota T, Ikeda T, Kuwahara S. Pharmacokinetics of cs-023 (ro4908463), a novel parenteral carbapenem, in healthy male caucasian volunteers. Antimicrob Agents Chemother. 2006;50(12):4186.

465. Doose D, Walker S, Gisclon L, Nayak R. Single-dose pharmacokinetics and effect of food on the bioavailability of topiramate, a novel antiepileptic drug. J Clin Pharmacol. 1996;36(10):884.

466. Perucca E. Pharmacokinetic profile of topiramate in comparison with other new antiepileptic drugs. Epilepsia. 1996;37:S8.

467. Johannessen S. Pharmacokinetics and interaction profile of topiramate: Review and comparison with other newer antiepileptic drugs. Epilepsia. 1997;38:S18.

468. Manitpisitkul P, Curtin CR, Shalayda K, Wang S-S, Ford L, Heald DL. Pharmacokinetics of topiramate in patients with renal impairment, end-stage renal disease undergoing hemodialysis, or hepatic impairment. Epilepsy Res. 2014;108(5):891-901.

469. Baethke R, Golde G, Gahl G. Sulphamethoxazole/trimethoprim: Pharmacokinetic studies in patients with chronic renal failure. Eur J Clin Pharmacol. 1972;4(4):233-40.

470. Andreasen F, Elsborg L, Husted S, Thomsen O. Pharmacokinetics of sulfadiazine and trimethoprim in man. Eur J Clin Pharmacol. 1978;14(1):57.

471. Wijkström A, Westerlund D. Plasma protein binding of sulphadiazine, sulphamethoxazole and trimethoprim determined by ultrafiltration. J Pharm Biomed Anal. 1983;1(3):293.

472. Teng R, Harris S, Nix D, Schentag J, Foulds G, Liston T. Pharmacokinetics and safety of trovafloxacin (cp-99,219), a new quinolone antibiotic, following administration of single oral doses to healthy male volunteers. J Antimicrob Chemoth. 1995;36(2):385.

473. Teng R, Liston T, Harris S. Multiple-dose pharmacokinetics and safety of trovafloxacin in healthy volunteers. J Antimicrob Chemoth. 1996;37(5):955.

474. Vincent J, Venitz J, Teng R, Baris B, Willavize S, Polzer R, et al. Pharmacokinetics and safety of trovafloxacin in healthy male volunteers following administration of single intravenous doses of the prodrug, alatrofloxacin. J Antimicrob Chemoth. 1997;39:75.

475. Gugler R, Schell A, Eichelbaum M, Fröscher W, Schulz H. Disposition of valproic acid in man. Eur J Clin Pharmacol. 1977;12(2):125.

476. Cramer J, Mattson R. Valproic acid: In vitro plasma protein binding and interaction with phenytoin. Ther Drug Monit. 1979;1(1):105.

477. Colussi D, Parisot C, Rossolino M, Brunner L, Lefèvre G. Protein binding in plasma of valsartan, a new angiotensin II receptor antagonist. J Clin Pharmacol. 1997;37(3):214.

478. Flesch G, Müller P, Lloyd P. Absolute bioavailability and pharmacokinetics of valsartan, an angiotensin II receptor antagonist, in man. Eur J Clin Pharmacol. 1997;52(2):115.

479. Faessel H, Gibbs M, Clark D, Rohrbacher K, Stolar M, Burstein A. Multiple-dose pharmacokinetics of the selective nicotinic receptor partial agonist, varenicline, in healthy smokers. J Clin Pharmacol. 2006;46(12):1439.

480. Feng B, Obach R, Burstein A, Clark D, de Morais S, Faessel H. Effect of human renal cationic transporter inhibition on the pharmacokinetics of varenicline, a new therapy for smoking cessation: An in vitro-in vivo study. Clin Pharmacol Ther. 2008;83(4):567.

481. Faessel H, Obach R, Rollema H, Ravva P, Williams K, Burstein A. A review of the clinical pharmacokinetics and pharmacodynamics of varenicline for smoking cessation. Clin Pharmacokinet. 2010;49(12):799.

482. Rollema H, Shrikhande A, Ward K, Tingley III F, Coe J, O'Neill B, et al. Pre-clinical properties of the α4β2 nicotinic acetylcholine receptor partial agonists varenicline, cytisine and dianicline translate to clinical efficacy for nicotine dependence. Br J Pharmacol. 2010;160(2):334.

483. Kikkawa H, Maruyama N, Fujimoto Y, Hasunuma T. Single-and multiple-dose pharmacokinetics of the selective nicotinic receptor partial agonist, varenicline, in healthy japanese adult smokers. J Clin Pharmacol. 2011;51(4):527.

484. DeVane C. Pharmacokinetics of the newer antidepressants: Clinical relevance. Am J Med. 1994;97(6A):13S.

485. Troy S, Schultz R, Parker V, Chiang S, Blum R. The effect of renal disease on the disposition of venlafaxine. Clin Pharmacol Ther. 1994;56(1):14.

486. Troy S, Parker V, Hicks D, Boudino F, Chiang S. Pharmacokinetic interaction between multiple-dose venlafaxine and single-dose lithium. J Clin Pharmacol. 1996;36(2):175.

487. Lessard E, Yessine M, Hamelin B, Gauvin C, Labbé L, O'Hara G, et al. Diphenhydramine alters the disposition of venlafaxine through inhibition of CYP2D6 activity in humans. J Clin Psychopharmacol. 2001;21(2):175.

488. Lombardo F, Obach R, Shalaeva M, Gao F. Prediction of human volume of distribution values for neutral and basic drugs. 2. Extended data set and leave-class-out statistics. J Med Chem. 2004;47(5):1242.

489. Mikus G, Eichelbaum M, Fischer C, Gumulka S, Klotz U, Kroemer H. Interaction of verapamil and cimetidine: Stereochemical aspects of drug metabolism, drug disposition and drug action. J Pharmacol Exp Ther. 1990;253(3):1042.

490. Lutsar I, Roffey S, Troke P. Voriconazole concentrations in the cerebrospinal fluid and brain tissue of guinea pigs and immunocompromised patients. Clin Infect Dis. 2003;37(5):728.

491. Mikus G, Schöwel V, Drzewinska M, Rengelshausen J, Ding R, Riedel K, et al. Potent cytochrome P450 2c19 genotype-related interaction between voriconazole and the cytochrome P450 3a4 inhibitor ritonavir. Clin Pharmacol Ther. 2006;80(2):126.

492. Scholz I, Oberwittler H, Riedel K-D, Burhenne J, Weiss J, Haefeli WE, et al. Pharmacokinetics, metabolism and bioavailability of the triazole antifungal agent voriconazole in relation to cyp2c19 genotype. Br J Clin Pharmacol. 2009;68(6):906.

493. Rengelshausen J, Banfield M, Riedel K, Burhenne J, Weiss J, Thomsen T, et al. Opposite effects of short-term and long-term st john's wort intake on voriconazole pharmacokinetics. Clin Pharmacol Ther. 2005;78(1):25.

494. Roffey S, Cole S, Comby P, Gibson D, Jezequel S, Nedderman A, et al. The disposition of voriconazole in mouse, rat, rabbit, guinea pig, dog, and human. Drug Metab Dispos. 2003;31(6):731.

495. Weiss J, Ten Hoevel M, Burhenne J, Walter-Sack I, Hoffmann M, Rengelshausen J, et al. Cyp2c19 genotype is a major factor contributing to the highly variable pharmacokinetics of voriconazole. J Clin Pharmacol. 2009;49(2):196.

496. Cass L, Brown J, Pickford M, Fayinka S, Newman S, Johansson C, et al. Pharmacoscintigraphic evaluation of lung deposition of inhaled zanamivir in healthy volunteers. Clin Pharmacokinet. 1999;36:21.

497. Weller S, Jones LS, Lou Y, Peppercorn A, Ng-Cashin J. Pharmacokinetics of zanamivir following intravenous administration to subjects with and without renal impairment. Antimicrob Agents Chemother. 2013;57(7):2967.

498. Cass L, Efthymiopoulos C, Bye A. Pharmacokinetics of zanamivir after intravenous, oral, inhaled or intranasal administration to healthy volunteers. Clin Pharmacokinet. 1999;36:1.

499. Daniel M, Barnett J, Pearson B. The low potential for drug interactions with zanamivir. Clin Pharmacokinet. 1999;36:41.

500. Cass L, Efthymiopoulos C, Marsh J, Bye A. Effect of renal impairment on the pharmacokinetics of intravenous zanamivir. Clin Pharmacokinet. 1999;36:13.

501. Singlas E, Pioger J, Taburet A, Colin J, Fillastre J. Zidovudine disposition in patients with severe renal impairment: Influence of hemodialysis. Clin Pharmacol Ther. 1989;46(2):190.

502. Hedaya M, Elmquist W, Sawchuk R. Probenecid inhibits the metabolic and renal clearances of zidovudine (azt) in human volunteers. Pharm Res. 1990;7(4):411.

503. Luzier A, Morse G. Intravascular distribution of zidovudine: Role of plasma proteins and whole blood components. Antiviral Res. 1993;21(3):267.

504. Fernandez C, Gimenez F, Thuillier A, Farinotti R. Stereoselective binding of zopiclone to human plasma proteins. Chirality. 1999;11(2):129.

505. Fernandez C, Maradeix V, Gimenez F, Thuillier A, Farinotti R. Pharmacokinetics of zopiclone and its enantiomers in caucasian young healthy volunteers. Drug Metab Dispos. 1993;21(6):1125.

506. Gaillot J, Heusse D, Hougton G, Marc AJ, Dreyfus J. Pharmacokinetics and metabolism of zopiclone. Pharmacology. 1983;27:76.

507. Tornio A, Neuvonen P, Backman J. The cyp2c8 inhibitor gemfibrozil does not increase the plasma concentrations of zopiclone. Eur J Clin Pharmacol. 2006;62(8):645.

508. Marc-Aurele J, Caille G, Bourgoin J. Comparison of zopiclone pharmacokinetics in patients with impaired renal function and normal subjects. Effect of hemodialysis. Sleep. 1987;10:22.
