# Supplementary material for: Novel minimal physiologically-based model for the prediction of passive tubular reabsorption and renal excretion clearance
Source: Eur J Pharm Sci. 2016 Oct 30;94:59–71. doi: 10.1016/j.ejps.2016.03.018 (PMC5074076; doi:10.1016/j.ejps.2016.03.018)
Supplement: Supplementary file 3 — Supplementary Results (Table S3.1) [file mmc3.docx]

**Supplementary Results (Table S3.1) for the manuscript: “Novel minimal physiologically-based model for the prediction of passive tubular reabsorption and renal excretion clearance”**

Daniel Scotcher ^a^, Christopher Jones ^b^, Amin Rostami-Hodjegan ^a,c^ and Aleksandra Galetin ^a^

^a^ Centre for Applied Pharmacokinetic Research, Manchester Pharmacy School, University of Manchester, Manchester, United Kingdom

^b^ Oncology iMed, AstraZeneca, Alderley Park, United Kingdom

^c^ Simcyp Limited (a Certara Company), Sheffield, United Kingdom

**Table S3.1. Human kidney drug transporters reported to interact with drugs in database of 157 drugs (at substrate level)**.

| **Drug** | **Kidney Transporters** | **References** |
| --- | --- | --- |
| Acebutolol |  |  |
| Acecainide |  |  |
| Acetaminophen |  |  |
| Acyclovir | OAT1, MATE1, MATE2K | (1, 2) |
| Adefovir | OAT1, OAT3, MRP4 | (1-3) |
| Allopurinol |  |  |
| Almotriptan |  |  |
| Amantadine | OCT2 | (1, 2, 4) |
| Amifloxacin |  |  |
| Amoxicillin | Conflicting data on OAT1 | (1, 5, 6) |
| Ampicillin | MRP2, MRP4 | (7-9) |
| Antipyrine |  |  |
| Apalcillin |  |  |
| Aprindine |  |  |
| Atenolol | OCT2 | (10) |
| Azlocillin | In vivo DDI evidence only | (11) |
| Aztreonam |  |  |
| Benzylpenicillin | OAT1, OAT3, MRP2, MRP4, OATP4C1 | (2, 9, 12-15) |
| Betamethasone | P-gp | (2) |
| Betaxolol |  |  |
| Bisoprolol | P-gp, In vivo DDI | (16, 17) |
| Caffeine |  |  |
| Captopril | OAT1, OAT3 | (18-20) |
| Carbenicillin | In vivo DDI evidence only | (21) |
| Cefamandole | In vivo DDI evidence; Conflicting data on MRP2 | (22, 23) |
| Cefazolin | OAT3, MRP4 | (19, 20, 24, 25) |
| Cefepime |  |  |
| Cefixime | Weak MRP4 interaction | (2, 26) |
| Cefmetazole | MRP4, In vivo DDI | (1, 9, 24) |
| Cefodizime |  |  |
| Cefonicid | In vivo DDI evidence only | (1) |
| Cefoperazone | MRP2, MRP4 | (24, 27, 28) |
| Ceforanide |  |  |
| Cefotaxime | OAT3, MRP4 | (24, 29) |
| Cefotetan | MRP2 | (28) |
| Cefotiam | OAT3, MRP2 | (28, 29) |
| Cefpirome |  |  |
| Ceftazidime | MRP4 | (9) |
| Ceftizoxime | OAT1, OAT3, MRP4 | (1, 20, 24) |
| Chloroquine | MATE1 | (30) |
| Chlorpheniramine |  |  |
| Chlorpropamide |  |  |
| Chlorthalidone |  |  |
| Cimetidine | OAT1, OAT3, OCT2, MATE1, MATE2K, P-gp, BCRP | (1, 2, 31) |
| Ciprofloxacin | MATE-1, P-gp, BCRP, In vivo DDI | (1, 32-36) |
| Citalopram |  |  |
| Clinafloxacin |  |  |
| Dapsone |  |  |
| Desipramine |  |  |
| Dexrazoxane |  |  |
| Difloxacin |  |  |
| Digoxin | P-gp, OATP4C1 | (1, 2, 37) |
| Diltiazem | P-gp | (2) |
| Dofetilide | In vivo DDI evidence only | (1) |
| Doxepin |  |  |
| Enoxacin | Weak P-gp interaction, In vivo DDI | (33, 38-40) |
| Enoximone |  |  |
| Enprofylline | In vivo DDI evidence only | (41) |
| Fexofenadine | OAT3, MATE1, P-gp, In vivo DDI | (1, 2, 42-45) |
| Fleroxacin | Weak P-gp interaction; In vivo DDI | (46, 47) |
| Flucloxacillin | In vivo DDI evidence only | (48) |
| Fluconazole | P-gp | (49) |
| Frovatriptan | OCT2 | (50) |
| Furosemide | OAT1, OAT3, MRP2, MRP4, In vivo DDI | (1, 2, 12, 51, 52) |
| Gabapentin | OCTN1 | (53) |
| Galantamine |  |  |
| Garenoxacin |  |  |
| Gatifloxacin | In vivo DDI evidence only | (54) |
| Gefitinib | P-gp, BCRP | (55-57) |
| Gemifloxacin | P-gp, MRP2, BCRP, In vivo DDI | (58-60) |
| Grepafloxacin | P-gp | (61, 62) |
| Imipramine |  |  |
| Irbesartan |  |  |
| Isoxicam |  |  |
| Lamivudine | OCT2, MATE1, MATE2K, BCRP, Weak P-gp interaction | (1, 63-69) |
| Lamotrigine | P-gp | (70-72) |
| Lenalidomide | Conflicting data on P-gp | (73, 74) |
| Levetiracetam |  |  |
| Levofloxacin | P-gp | (19) |
| Linezolid |  |  |
| Lomefloxacin | In vivo DDI evidence only | (1, 38) |
| Lorazepam |  |  |
| Maraviroc | P-gp | (75) |
| MDMA |  |  |
| Melagatran | P-gp | (76) |
| Memantine | OCT2 | (1, 2, 4) |
| Mesna | MATE1, P-gp, MRP2, OAT4, In vivo DDI | (77) |
| Metformin | OCT2, MATE1 MATE2K | (1, 19, 35, 78-82) |
| Methadone | Conflicting data on P-gp | (2, 83-86) |
| Metoprolol | OCT2 | (87) |
| Metronidazole |  |  |
| Mexiletine |  |  |
| Moclobemide |  |  |
| Morphine | P-gp | (2) |
| Moxalactam |  |  |
| Moxifloxacin | P-gp, MRP2 | (88, 89) |
| Nafcillin | P-gp, Weak BCRP interaction, In vivo DDI | (34, 90, 91) |
| Ofloxacin | OCT2 | (10) |
| Olmesartan | OAT1, OAT3, MRP2, MRP4 | (1, 2, 92, 93) |
| Oseltamivir carboxylate | OAT1, OAT3, MRP4 | (5, 94) |
| Oxprenolol |  |  |
| Oxytetracycline | P-gp | (95) |
| Pefloxacin | P-gp | (96) |
| Penciclovir | MRP4; Conflicting data on OAT1 and OAT3 | (97-99) |
| Pilsicainide | In vivo DDI evidence only | (1) |
| Pindolol | OCT2, In vivo DDI | (1, 10) |
| Piperacillin | MRP4 | (9) |
| Pravastatin | OAT3, OAT4, MRP2, MRP4 | (1, 9, 100, 101) |
| Prednisolone | P-gp | (2, 102) |
| Prednisone | Weak P-gp interaction | (2, 102) |
| Probenecid |  |  |
| Procainamide | OCT2, MATE1, MATE2K, In vivo DDI | (1, 103-105) |
| Promethazine |  |  |
| Propafenone |  |  |
| Propylthiouracil |  |  |
| Pyrazinamide |  |  |
| Quinidine | OCTN1, OCTN2 | (1, 106, 107) |
| Raltegravir | OAT1 | (108) |
| Remoxipride |  |  |
| Resveratrol | BCRP | (109) |
| Ribavirin |  |  |
| Rifabutin |  |  |
| Rifampin |  |  |
| Risperidone | P-gp | (110) |
| Rivaroxaban | P-gp | (111) |
| Ropivacaine |  |  |
| Rosuvastatin | OAT3, P-gp, MRP2, MRP4, BCRP | (1, 112-114) |
| Rufloxacin |  |  |
| Salbutamol | OCT2 | (115) |
| Sematilide |  |  |
| Sitagliptin | OAT3, P-gp, OATP4C1 | (116) |
| Sparfloxacin | P-gp | (46) |
| Sulfamethoxazole |  |  |
| Telbivudine |  |  |
| Temafloxacin | In vivo evidence only | (117) |
| Temocillin |  |  |
| Tenofovir | OAT1, OAT3, P-gp, MRP4, BCRP | (3, 118, 119) |
| Terodiline |  |  |
| Tetracycline | Weak OAT3 interaction | (1, 120) |
| Theophylline |  |  |
| Timolol |  |  |
| Tinidazole |  |  |
| Tizanidine |  |  |
| Tocainide |  |  |
| Tomopenem |  |  |
| Topiramate | MATE2K | (121) |
| Trimethoprim |  |  |
| Trovafloxacin |  |  |
| Valproic Acid |  |  |
| Valsartan | Weak P-gp interaction, MRP2 | (1, 122) |
| Varenicline | OCT2, MATE1, MATE2K | (1, 123, 124) |
| Venlafaxine |  |  |
| Verapamil | P-gp, OCTN1, OCTN2 | (107, 125, 126) |
| Voriconazole |  |  |
| Zanamivir |  |  |
| Zidovudine | OAT1, OAT3, OAT4, Weak P-gp interaction | (1, 64, 65, 127) |
| Zopiclone |  |  |

**References**

1. Morrissey K, Wen C, Johns S, Zhang L, Huang S, Giacomini K. The UCSF-FDA TransPortal: A Public Drug Transporter Database. Clin Pharmacol Ther. 2012;92(5):545.

2. Ozawa N, Shimizu T, Morita R, Yokono Y, Ochiai T, Munesada K, et al. Transporter database, TP-Search: a web-accessible comprehensive database for research in pharmacokinetics of drugs. Pharm Res. 2004;21(11):2133.

3. Uwai Y, Ida H, Tsuji Y, Katsura T, Inui K. Renal transport of adefovir, cidofovir, and tenofovir by SLC22A family members (hOAT1, hOAT3, and hOCT2). Pharm Res. 2007;24(4):811.

4. Busch A, Karbach U, Miska D, Gorboulev V, Akhoundova A, Volk C, et al. Human neurons express the polyspecific cation transporter hOCT2, which translocates monoamine neurotransmitters, amantadine, and memantine. Mol Pharmacol. 1998;54(2):342.

5. Hill G, Cihlar T, Oo C, Ho E, Prior K, Wiltshire H, et al. The anti-influenza drug oseltamivir exhibits low potential to induce pharmacokinetic drug interactions via renal secretion-correlation of in vivo and in vitro studies. Drug Metab Dispos. 2002;30(1):13.

6. Li M, Anderson G, Phillips B, Kong W, Shen D, Wang J. Interactions of amoxicillin and cefaclor with human renal organic anion and peptide transporters. Drug Metab Dispos. 2006;34(4):547.

7. Gerk P, Vore M. Regulation of expression of the multidrug resistance-associated protein 2 (MRP2) and its role in drug disposition. J Pharmacol Exp Ther. 2002;302(2):407.

8. Jedlitschky G, Hoffmann U, Kroemer H. Structure and function of the MRP2 (ABCC2) protein and its role in drug disposition. Expert Opin Drug Metab Toxicol. 2006;2(3):351.

9. Uchida Y, Kamiie J, Ohtsuki S, Terasaki T. Multichannel liquid chromatography-tandem mass spectrometry cocktail method for comprehensive substrate characterization of multidrug resistance-associated protein 4 transporter. Pharm Res. 2007;24(12):2281.

10. Ciarimboli G, Schröter R, Neugebauer U, Vollenbröker B, Gabriëls G, Brzica H, et al. Kidney transplantation down-regulates expression of organic cation transporters, which translocate β-blockers and fluoroquinolones. Mol Pharm. 2013;10(6):2370.

11. Leroy A, Humbert G, Fillastre J. Pharmacokinetics of azlocillin in healthy subjects. Scand J Infect Dis Suppl. 1981;29:49.

12. Bakos E, Evers R, Sinkó E, Váradi A, Borst P, Sarkadi B. Interactions of the human multidrug resistance proteins MRP1 and MRP2 with organic anions. Mol Pharmacol. 2000;57(4):760.

13. Choi M, Kim H, Han Y, Song I, Shim C. Involvement of Mrp2/MRP2 in the species different excretion route of benzylpenicillin between rat and human. Xenobiotica. 2009;39(2):171.

14. Tahara H, Shono M, Kusuhara H, Kinoshita H, Fuse E, Takadate A, et al. Molecular cloning and functional analyses of OAT1 and OAT3 from cynomolgus monkey kidney. Pharm Res. 2005;22(4):647.

15. Zelcer N, Huisman M, Reid G, Wielinga P, Breedveld P, Kuil A, et al. Evidence for two interacting ligand binding sites in human multidrug resistance protein 2 (ATP binding cassette C2). J Biol Chem. 2003;278(26):23538.

16. Bachmakov I, Werner U, Endress B, Auge D, Fromm M. Characterization of beta-adrenoceptor antagonists as substrates and inhibitors of the drug transporter P-glycoprotein. Fundam Clin Pharmacol. 2006;20(3):273.

17. Kirch W, Rose I, Klingmann I, Pabst J, Ohnhaus E. Interaction of bisoprolol with cimetidine and rifampicin. Eur J Clin Pharmacol. 1986;31(1):59.

18. Kelety B, Diekert K, Tobien J, Watzke N, Dörner W, Obrdlik P, et al. Transporter assays using solid supported membranes: a novel screening platform for drug discovery. Assay Drug Dev Technol. 2006;4(5):575.

19. Tanihara Y, Masuda S, Sato T, Katsura T, Ogawa O, Inui K. Substrate specificity of MATE1 and MATE2-K, human multidrug and toxin extrusions/H (+)-organic cation antiporters. Biochem Pharmacol. 2007;74(2):359.

20. Ueo H, Motohashi H, Katsura T, Inui K. Human organic anion transporter hOAT3 is a potent transporter of cephalosporin antibiotics, in comparison with hOAT1. Biochem Pharmacol. 2005;70(7):1104.

21. Itoh T, Ishida M, Onuki Y, Tsuda Y, Shimada H, Yamada H. Stereoselective renal tubular secretion of carbenicillin. Antimicrob Agents Chemother. 1993;37(11):2327.

22. Griffith R, Black H, Brier G, Wolny J. Effect of Probenecid on the Blood Levels and Urinary Excretion of Cefamandole. Antimicrob Agents Chemother. 1977;11(5):809.

23. Pedersen J, Matsson P, Bergström C, Norinder U, Hoogstraate J, Artursson P. Prediction and identification of drug interactions with the human ATP-binding cassette transporter multidrug-resistance associated protein 2 (MRP2; ABCC2). J Med Chem. 2008;51(11):3275.

24. Ci L, Kusuhara H, Adachi M, Schuetz J, Takeuchi K, Sugiyama Y. Involvement of MRP4 (ABCC4) in the luminal efflux of ceftizoxime and cefazolin in the kidney. Mol Pharmacol. 2007;71(6):1591.

25. Sakurai Y, Motohashi H, Ueo H, Masuda S, Saito H, Okuda M, et al. Expression levels of renal organic anion transporters (OATs) and their correlation with anionic drug excretion in patients with renal diseases. Pharm Res. 2004;21(1):61.

26. Russel F, Koenderink J, Masereeuw R. Multidrug resistance protein 4 (MRP4/ABCC4): a versatile efflux transporter for drugs and signalling molecules. Trends Pharmacol Sci. 2008;29(4):200.

27. Akanuma S, Uchida Y, Ohtsuki S, Kamiie J, Tachikawa M, Terasaki T, et al. Molecular-weight-dependent, anionic-substrate-preferential transport of β-lactam antibiotics via multidrug resistance-associated protein 4. Drug Metab Pharmacokinet. 2011;26(6):602.

28. Kato Y, Takahara S, Kato S, Kubo Y, Sai Y, Tamai I, et al. Involvement of multidrug resistance-associated protein 2 (Abcc2) in molecular weight-dependent biliary excretion of beta-lactam antibiotics. Drug Metab Dispos. 2008;36(6):1088.

29. Yee SW, Nguyen AN, Brown C, Savic RM, Zhang Y, Castro RA, et al. Reduced Renal Clearance of Cefotaxime in Asians with a Low-Frequency Polymorphism of OAT3 (SLC22A8). J Pharm Sci. 2013;102(9):3451.

30. Müller F, König J, Glaeser H, Schmidt I, Zolk O, Fromm MF, et al. Molecular Mechanism of Renal Tubular Secretion of the Antimalarial Drug Chloroquine. Antimicrob Agents Chemother. 2011;55(7):3091.

31. Pavek P, Merino G, Wagenaar E, Bolscher E, Novotna M, Jonker J, et al. Human breast cancer resistance protein: interactions with steroid drugs, hormones, the dietary carcinogen 2-amino-1-methyl-6-phenylimidazo (4, 5-b) pyridine, and transport of cimetidine. J Pharmacol Exp Ther. 2005;312(1):144.

32. Cavet M, West M, Simmons N. Fluoroquinolone (ciprofloxacin) secretion by human intestinal epithelial (Caco-2) cells. Br J Pharmacol. 1997;121(8):1567.

33. Maeda T, Takahashi K, Ohtsu N, Oguma T, Ohnishi T, Atsumi R, et al. Identification of influx transporter for the quinolone antibacterial agent levofloxacin. Mol Pharm. 2007;4(1):85-94.

34. Merino G, Alvarez A, Pulido M, Molina A, Schinkel A, Prieto J. Breast cancer resistance protein (BCRP/ABCG2) transports fluoroquinolone antibiotics and affects their oral availability, pharmacokinetics, and milk secretion. Drug Metab Dispos. 2006;34(4):690.

35. Meyer zSH, Verstuyft C, Kroemer H, Becquemont L, Kim R. Human multidrug and toxin extrusion 1 (MATE1/SLC47A1) transporter: functional characterization, interaction with OCT2 (SLC22A2), and single nucleotide polymorphisms. Am J Physiol Renal Physiol. 2010;298(4):F997.

36. Park MS, Okochi H, Benet LZ. Is Ciprofloxacin a Substrate of P-glycoprotein? Arch Drug Inf. 2011;4(1):1.

37. Mikkaichi T, Suzuki T, Onogawa T, Tanemoto M, Mizutamari H, Okada M, et al. Isolation and characterization of a digoxin transporter and its rat homologue expressed in the kidney. Proc Natl Acad Sci U S A. 2004;101(10):3569.

38. Crivori P, Reinach B, Pezzetta D, Poggesi I. Computational models for identifying potential P-glycoprotein substrates and inhibitors. Mol Pharm. 2006;3(1):33.

39. Misiak P, Eldon M, Toothaker R, Sedman A. Effects of oral cimetidine or ranitidine on the pharmacokinetics of intravenous enoxacin. J Clin Pharmacol. 1993;33(1):53.

40. Wijnands W, Vree T, Baars A, van Herwaarden C. Pharmacokinetics of enoxacin and its penetration into bronchial secretions and lung tissue. J Antimicrob Chemoth. 1988;21:67.

41. Borgå O, Larsson R, Lunell E. Effects of probenecid on enprofylline kinetics in man. Eur J Clin Pharmacol. 1986;30(2):221.

42. Kusuhara H, Miura M, Yasui-Furukori N, Yoshida K, Akamine Y, Yokochi M, et al. Effect of coadministration of single and multiple doses of rifampicin on the pharmacokinetics of fexofenadine enantiomers in healthy subjects. Drug Metab Dispos. 2013;41(1):206.

43. Matsushima S, Maeda K, Inoue K, Ohta K, Yuasa H, Kondo T, et al. The inhibition of human multidrug and toxin extrusion 1 is involved in the drug-drug interaction caused by cimetidine. Drug Metab Dispos. 2009;37(3):555.

44. Tahara H, Kusuhara H, Maeda K, Koepsell H, Fuse E, Sugiyama Y. Inhibition of oat3-mediated renal uptake as a mechanism for drug-drug interaction between fexofenadine and probenecid. Drug Metab Dispos. 2006;34(5):743.

45. Yasui-Furukori N, Uno T, Sugawara K, Tateishi T. Different effects of three transporting inhibitors, verapamil, cimetidine, and probenecid, on fexofenadine pharmacokinetics. Clin Pharmacol Ther. 2005;77(1):17.

46. de Lange E, Marchand S, van den Berg D, van der Sandt I, de Boer A, Delon A, et al. In vitro and in vivo investigations on fluoroquinolones; effects of the P-glycoprotein efflux transporter on brain distribution of sparfloxacin. Eur J Pharm Sci. 2000;12(2):85.

47. Shiba K, Saito A, Shimada J, Hori S, Kaji M, Miyahara T, et al. Renal handling of fleroxacin in rabbits, dogs, and humans. Antimicrob Agents Chemother. 1990;34(1):58.

48. Landersdorfer CB, Kirkpatrick CM, Kinzig M, Bulitta JB, Holzgrabe U, Sörgel F. Inhibition of flucloxacillin tubular renal secretion by piperacillin. Br J Clin Pharmacol. 2008;66(5):648.

49. Wang E-j, Lew K, Casciano CN, Clement RP, Johnson WW. Interaction of Common Azole Antifungals with P Glycoprotein. Antimicrob Agents Chemother. 2002;46(1):160.

50. Ito S, Ando H, Ose A, Kitamura Y, Ando T, Kusuhara H, et al. Relationship between the urinary excretion mechanisms of drugs and their physicochemical properties. J Pharm Sci. 2013;102(9):3294.

51. Hasannejad H, Takeda M, Taki K, Shin H, Babu E, Jutabha P, et al. Interactions of human organic anion transporters with diuretics. J Pharmacol Exp Ther. 2004;308(3):1021.

52. Hasegawa M, Kusuhara H, Adachi M, Schuetz J, Takeuchi K, Sugiyama Y. Multidrug resistance-associated protein 4 is involved in the urinary excretion of hydrochlorothiazide and furosemide. J Am Soc Neph. 2007;18(1):37.

53. Urban T, Brown C, Castro R, Shah N, Mercer R, Huang Y, et al. Effects of genetic variation in the novel organic cation transporter, OCTN1, on the renal clearance of gabapentin. Clin Pharmacol Ther. 2008;83(3):416.

54. Nakashima M, Uematsu T, Kosuge K, Kusajima H, Ooie T, Masuda Y, et al. Single-and multiple-dose pharmacokinetics of AM-1155, a new 6-fluoro-8-methoxy quinolone, in humans. Antimicrob Agents Chemother. 1995;39(12):2635.

55. Agarwal S, Sane R, Gallardo JL, Ohlfest JR, Elmquist WF. Distribution of Gefitinib to the Brain Is Limited by P-glycoprotein (ABCB1) and Breast Cancer Resistance Protein (ABCG2)-Mediated Active Efflux. J Pharmacol Exp Ther. 2010;334(1):147.

56. Cusatis G, Gregorc V, Li J, Spreafico A, Ingersoll R, Verweij J, et al. Pharmacogenetics of ABCG2 and adverse reactions to gefitinib. J Natl Cancer Inst. 2006;98(23):1739.

57. Ozvegy-Laczka C, Hegedus T, Várady G, Ujhelly O, Schuetz J, Váradi A, et al. High-affinity interaction of tyrosine kinase inhibitors with the ABCG2 multidrug transporter. Mol Pharmacol. 2004;65(6):1485.

58. Jin H, Song B, Kim S, Shim W, Kim D, Chong S, et al. Transport of gemifloxacin, a 4th generation quinolone antibiotic, in the Caco-2 and engineered MDCKII cells, and potential involvement of efflux transporters in the intestinal absorption of the drug. Xenobiotica. 2013;43(4):355.

59. Landersdorfer CB, Kirkpatrick CM, Kinzig M, Bulitta JB, Holzgrabe U, Drusano GL, et al. Competitive Inhibition of Renal Tubular Secretion of Gemifloxacin by Probenecid. Antimicrob Agents Chemother. 2009;53(9):3902.

60. Vadlapatla RK, Vadlapudi AD, Kwatra D, Pal D, Mitra AK. Differential effect of P-gp and MRP2 on cellular translocation of gemifloxacin. Int J Pharm. 2011;420(1):26.

61. Lowes S, Simmons NL. Multiple pathways for fluoroquinolone secretion by human intestinal epithelial (Caco-2) cells. Br J Pharmacol. 2002;135(5):1263.

62. Naruhashi K, Tamai I, Inoue N, Muraoka H, Sai Y, Suzuki N, et al. Active intestinal secretion of new quinolone antimicrobials and the partial contribution of P-glycoprotein. J Pharm Pharmacol. 2001;53(5):699.

63. Anderson P, Lamba J, Aquilante C, Schuetz E, Fletcher C. Pharmacogenetic characteristics of indinavir, zidovudine, and lamivudine therapy in HIV-infected adults: a pilot study. J Acquir Immune Defic Syndr. 2006;42(4):441.

64. de Souza J, Benet L, Huang Y, Storpirtis S. Comparison of bidirectional lamivudine and zidovudine transport using MDCK, MDCK-MDR1, and Caco-2 cell monolayers. J Pharm Sci. 2009;98(11):4413.

65. Jung N, Lehmann C, Rubbert A, Knispel M, Hartmann P, van Lunzen J, et al. Relevance of the organic cation transporters 1 and 2 for antiretroviral drug therapy in human immunodeficiency virus infection. Drug Metab Dispos. 2008;36(8):1616.

66. Kim H-S, Sunwoo YE, Ryu JY, Kang H-J, Jung H-E, Song I-S, et al. The effect of ABCG2 V12M, Q141K and Q126X, known functional variants in vitro, on the disposition of lamivudine. Br J Clin Pharmacol. 2007;64(5):645.

67. Minuesa G, Volk C, Molina-Arcas M, Gorboulev V, Erkizia I, Arndt P, et al. Transport of lamivudine [(-)-beta-L-2', 3'-dideoxy-3'-thiacytidine] and high-affinity interaction of nucleoside reverse transcriptase inhibitors with human organic cation transporters 1, 2, and 3. J Pharmacol Exp Ther. 2009;329(1):252.

68. Müller F, König J, Hoier E, Mandery K, Fromm M. Role of organic cation transporter OCT2 and multidrug and toxin extrusion proteins MATE1 and MATE2-K for transport and drug interactions of the antiviral lamivudine. Biochem Pharmacol. 2013;86(6):808.

69. Wang X, Furukawa T, Nitanda T, Okamoto M, Sugimoto Y, Akiyama S, et al. Breast cancer resistance protein (BCRP/ABCG2) induces cellular resistance to HIV-1 nucleoside reverse transcriptase inhibitors. Mol Pharmacol. 2003;63(1):65.

70. Crowe A, Teoh Y. Limited P-glycoprotein mediated efflux for anti-epileptic drugs. J Drug Target. 2006;14(5):291.

71. Luna-Tortós C, Fedrowitz M, Löscher W. Several major antiepileptic drugs are substrates for human P-glycoprotein. Neuropharmacology. 2008;55(8):1364.

72. Luna-Tortós C, Fedrowitz M, Löscher W. Evaluation of transport of common antiepileptic drugs by human multidrug resistance-associated proteins (MRP1, 2 and 5) that are overexpressed in pharmacoresistant epilepsy. Neuropharmacology. 2010;58(7):1019.

73. Hofmeister CC, Yang X, Pichiorri F, Chen P, Rozewski DM, Johnson AJ, et al. Phase I Trial of Lenalidomide and CCI-779 in Patients With Relapsed Multiple Myeloma: Evidence for Lenalidomide–CCI-779 Interaction via P-Glycoprotein. J Clin Oncol. 2011;29(25):3427.

74. Kumar G, Surapeneni S, Lau H, Laskin O, Fox L. Interaction of lenalidomide with human drug transporters in vitro. Drug Metab Rev. 2008;S3:282-3.

75. Walker D, Abel S, Comby P, Muirhead G, Nedderman A, Smith D. Species differences in the disposition of the CCR5 antagonist, UK-427,857, a new potential treatment for HIV. Drug Metab Dispos. 2005;33(4):587.

76. Darnell M, Karlsson J, Owen A, Hidalgo I, Li J, Zhang W, et al. Investigation of the involvement of P-glycoprotein and multidrug resistance-associated protein 2 in the efflux of ximelagatran and its metabolites by using short hairpin RNA knockdown in Caco-2 cells. Drug Metab Dispos. 2010;38(3):491.

77. Cutler M, Urquhart B, Velenosi T, Meyer ZSH, Dresser G, Leake B, et al. In vitro and in vivo assessment of renal drug transporters in the disposition of mesna and dimesna. J Clin Pharmacol. 2012;52(4):530.

78. Chen Y, Teranishi K, Li S, Yee SW, Hesselson S, Stryke D, et al. Genetic Variants in Multidrug and Toxic Compound Extrusion 1, hMATE1, Alter Transport Function. Pharmacogenom J. 2009;9(2):127.

79. Choi M, Jin Q, Jin H, Shim C, Cho D, Shin J, et al. Effects of tetraalkylammonium compounds with different affinities for organic cation transporters on the pharmacokinetics of metformin. Biopharm Drug Dispos. 2007;28(9):501.

80. Kimura N, Masuda S, Tanihara Y, Ueo H, Okuda M, Katsura T, et al. Metformin is a superior substrate for renal organic cation transporter OCT2 rather than hepatic OCT1. Drug Metab Pharmacokinet. 2005;20(5):379.

81. Masuda S, Terada T, Yonezawa A, Tanihara Y, Kishimoto K, Katsura T, et al. Identification and functional characterization of a new human kidney-specific H+/organic cation antiporter, kidney-specific multidrug and toxin extrusion 2. J Am Soc Neph. 2006;17(8):2127.

82. Zolk O, Solbach T, König J, Fromm M. Functional characterization of the human organic cation transporter 2 variant p. 270Ala> Ser. Drug Metab Dispos. 2009;37(6):1312.

83. Crettol S, Digon P, Golay K, Brawand M, Eap C. In vitro P-glycoprotein-mediated transport of (R)-,(S)-,(R, S)-methadone, LAAM and their main metabolites. Pharmacology. 2007;80(4):304.

84. Kharasch ED, Hoffer C, Whittington D. The effect of quinidine, used as a probe for the involvement of P-glycoprotein, on the intestinal absorption and pharmacodynamics of methadone. Br J Clin Pharmacol. 2004;57(5):600.

85. Nanovskaya T, Nekhayeva I, Karunaratne N, Audus K, Hankins GD, Ahmed MS. Role of P-glycoprotein in transplacental transfer of methadone. Biochem Pharmacol. 2005;69(12):1869.

86. Störmer E, Perloff M, von Moltke L, Greenblatt D. Methadone inhibits rhodamine123 transport in Caco-2 cells. Drug Metab Dispos. 2001;29(7):954.

87. Dudley AJ, Bleasby K, Brown CD. The organic cation transporter OCT2 mediates the uptake of β-adrenoceptor antagonists across the apical membrane of renal LLC-PK1 cell monolayers. Br J Pharmacol. 2000;131(1):71.

88. Barot M, Gokulgandhi M, Pal D, Mitra A. In vitro moxifloxacin drug interaction with chemotherapeutics: implications for retinoblastoma management. Exp Eye Res. 2014;118:61.

89. Brillault J, De Castro WV, Harnois T, Kitzis A, Olivier J-C, Couet W. P-Glycoprotein-Mediated Transport of Moxifloxacin in a Calu-3 Lung Epithelial Cell Model. Antimicrob Agents Chemother. 2009;53(4):1457.

90. Chang C, Bahadduri P, Polli J, Swaan P, Ekins S. Rapid identification of P-glycoprotein substrates and inhibitors. Drug Metab Dispos. 2006;34(12):1976.

91. Waller E, Sharanevych M, Yakatan G. The effect of probenecid on nafcillin disposition. J Clin Pharmacol. 1982;22(10):482.

92. Kamiyama E, Nakai D, Mikkaichi T, Okudaira N, Okazaki O. Interaction of angiotensin II type 1 receptor blockers with P-gp substrates in Caco-2 cells and hMDR1-expressing membranes. Life Sci. 2010;86(1-2):52.

93. Yamada A, Maeda K, Kamiyama E, Sugiyama D, Kondo T, Shiroyanagi Y, et al. Multiple human isoforms of drug transporters contribute to the hepatic and renal transport of olmesartan, a selective antagonist of the angiotensin II AT1-receptor. Drug Metab Dispos. 2007;35(12):2166.

94. Ose A, Ito M, Kusuhara H, Yamatsugu K, Kanai M, Shibasaki M, et al. Limited brain distribution of [3R, 4R, 5S]-4-acetamido-5-amino-3-(1-ethylpropoxy)-1-cyclohexene-1-carboxylate phosphate (Ro 64-0802), a pharmacologically active form of oseltamivir, by active efflux across the blood-brain barrier mediated by organic anion transporter 3 (Oat3/Slc22a8) and multidrug resistance-associated protein 4 (Mrp4/Abcc4). Drug Metab Dispos. 2009;37(2):315.

95. Schrickx J, Fink-Gremmels J. P-glycoprotein-mediated transport of oxytetracycline in the Caco-2 cell model. J Vet Pharmacol Ther. 2007;30(1):25.

96. Brillault J, De Castro W, Couet W. Relative contributions of active mediated transport and passive diffusion of fluoroquinolones with various lipophilicities in a Calu-3 lung epithelial cell model. Antimicrob Agents Chemother. 2010;54(1):543.

97. Cheng Y, Vapurcuyan A, Shahidullah M, Aleksunes L, Pelis R. Expression of organic anion transporter 2 in the human kidney and its potential role in the tubular secretion of guanine-containing antiviral drugs. Drug Metab Dispos. 2012;40(3):617.

98. Natrillo A, Vapurcuyan A, Cheng Y, Pelis R. Interaction of penciclovir with renal drug transporters (P334). 17th North American Regional ISSX Meeting October 16 - 20, 2011; Atlanta, GA, US2011.

99. Uwai Y, Honjo H, Iwamoto K. Interaction and transport of kynurenic acid via human organic anion transporters hOAT1 and hOAT3. Pharmacol Res. 2012;65(2):254-60.

100. Nakagomi-Hagihara R, Nakai D, Tokui T. Inhibition of human organic anion transporter 3 mediated pravastatin transport by gemfibrozil and the metabolites in humans. Xenobiotica. 2007;37(4):416.

101. Sasaki M, Suzuki H, Ito K, Abe T, Sugiyama Y. Transcellular transport of organic anions across a double-transfected Madin-Darby canine kidney II cell monolayer expressing both human organic anion-transporting polypeptide (OATP2/SLC21A6) and Multidrug resistance-associated protein 2 (MRP2/ABCC2). J Biol Chem. 2002;277(8):6497.

102. Yates C, Chang C, Kearbey J, Yasuda K, Schuetz E, Miller D, et al. Structural determinants of P-glycoprotein-mediated transport of glucocorticoids. Pharm Res. 2003;20(11):1794.

103. Bauer LA, Black DJ, Lill JS, Garrison J, Raisys VA, Hooton TM. Levofloxacin and Ciprofloxacin Decrease Procainamide and N-Acetylprocainamide Renal Clearances. Antimicrob Agents Chemother. 2005;49(4):1649.

104. Sato T, Masuda S, Yonezawa A, Tanihara Y, Katsura T, Inui K. Transcellular transport of organic cations in double-transfected MDCK cells expressing human organic cation transporters hOCT1/hMATE1 and hOCT2/hMATE1. Biochem Pharmacol. 2008;76(7):894.

105. Somogyi A, McLean A, Heinzow B. Cimetidine-procainamide pharmacokinetic interaction in man: evidence of competition for tubular secretion of basic drugs. Eur J Clin Pharmacol. 1983;25(3):339.

106. Ohashi R, Tamai I, Yabuuchi H, Nezu J, Oku A, Sai Y, et al. Na (+)-dependent carnitine transport by organic cation transporter (OCTN2): its pharmacological and toxicological relevance. J Pharmacol Exp Ther. 1999;291(2):778.

107. Yabuuchi H, Tamai I, Nezu J, Sakamoto K, Oku A, Shimane M, et al. Novel membrane transporter OCTN1 mediates multispecific, bidirectional, and pH-dependent transport of organic cations. J Pharmacol Exp Ther. 1999;289(2):768.

108. Moss DM, San Kwan W, Liptrott NJ, Smith DL, Siccardi M, Khoo SH, et al. Raltegravir Is a Substrate for SLC22A6: a Putative Mechanism for the Interaction between Raltegravir and Tenofovir. Antimicrob Agents Chemother. 2011;55(2):879.

109. Breedveld P, Pluim D, Cipriani G, Dahlhaus F, van Eijndhoven M, de Wolf C, et al. The effect of low pH on breast cancer resistance protein (ABCG2)-mediated transport of methotrexate, 7-hydroxymethotrexate, methotrexate diglutamate, folic acid, mitoxantrone, topotecan, and resveratrol in in vitro drug transport models. Mol Pharmacol. 2007;71(1):240.

110. Zhu H, Wang J, Markowitz J, Donovan J, Gibson B, DeVane C. Risperidone and paliperidone inhibit p-glycoprotein activity in vitro. Neuropsychopharmacology. 2007;32(4):757.

111. Gnoth M, Buetehorn U, Muenster U, Schwarz T, Sandmann S. In vitro and in vivo P-glycoprotein transport characteristics of rivaroxaban. J Pharmacol Exp Ther. 2011;338(1):372.

112. Li J, Volpe D, Wang Y, Zhang W, Bode C, Owen A, et al. Use of transporter knockdown Caco-2 cells to investigate the in vitro efflux of statin drugs. Drug Metab Dispos. 2011;39(7):1196.

113. Verhulst A, Sayer R, De Broe M, D'Haese P, Brown C. Human proximal tubular epithelium actively secretes but does not retain rosuvastatin. Mol Pharmacol. 2008;74(4):1084.

114. Windass A, Lowes S, Wang Y, Brown C. The contribution of organic anion transporters OAT1 and OAT3 to the renal uptake of rosuvastatin. J Pharmacol Exp Ther. 2007;322(3):1221.

115. Hendrickx R, Johansson J, Lohmann C, Jenvert R, Blomgren A, Börjesson L, et al. Identification of novel substrates and structure-activity relationship of cellular uptake mediated by human organic cation transporters 1 and 2. J Med Chem. 2013;56(18):7232.

116. Chu X, Bleasby K, Yabut J, Cai X, Chan G, Hafey M, et al. Transport of the dipeptidyl peptidase-4 inhibitor sitagliptin by human organic anion transporter 3, organic anion transporting polypeptide 4C1, and multidrug resistance P-glycoprotein. J Pharmacol Exp Ther. 2007;321(2):673.

117. Stein G. Drug interactions with fluoroquinolones. Am J Med. 1991;91(6A):81S.

118. Imaoka T, Kusuhara H, Adachi M, Schuetz J, Takeuchi K, Sugiyama Y. Functional involvement of multidrug resistance-associated protein 4 (MRP4/ABCC4) in the renal elimination of the antiviral drugs adefovir and tenofovir. Mol Pharmacol. 2007;71(2):619.

119. Neumanova Z, Cerveny L, Ceckova M, Staud F. Interactions of tenofovir and tenofovir disoproxil fumarate with drug efflux transporters ABCB1, ABCG2, and ABCC2; role in transport across the placenta. AIDS. 2014;28(1):9.

120. Babu E, Takeda M, Narikawa S, Kobayashi Y, Yamamoto T, Cha S, et al. Human organic anion transporters mediate the transport of tetracycline. Jap J Pharmacol. 2002;88(1):69.

121. Luna-Tortós C, Rambeck B, Jürgens U, Löscher W. The antiepileptic drug topiramate is a substrate for human P-glycoprotein but not multidrug resistance proteins. Pharm Res. 2009;26(11):2464.

122. Yamashiro W, Maeda K, Hirouchi M, Adachi Y, Hu Z, Sugiyama Y. Involvement of transporters in the hepatic uptake and biliary excretion of valsartan, a selective antagonist of the angiotensin II AT1-receptor, in humans. Drug Metab Dispos. 2006;34(7):1247.

123. Feng B, Obach R, Burstein A, Clark D, de Morais S, Faessel H. Effect of human renal cationic transporter inhibition on the pharmacokinetics of varenicline, a new therapy for smoking cessation: an in vitro-in vivo study. Clin Pharmacol Ther. 2008;83(4):567.

124. Kajiwara M, Masuda S, Watanabe S, Terada T, Katsura T, Inui K. Renal tubular secretion of varenicline by multidrug and toxin extrusion (MATE) transporters. Drug Metab Pharmacokinet. 2012;27(6):563.

125. Kimura Y, Kioka N, Kato H, Matsuo M, Ueda K. Modulation of drug-stimulated ATPase activity of human MDR1/P-glycoprotein by cholesterol. Biochem J. 2007;401(Pt 2):597.

126. Ohashi R, Tamai I, Inano A, Katsura M, Sai Y, Nezu J, et al. Studies on functional sites of organic cation/carnitine transporter OCTN2 (SLC22A5) using a Ser467Cys mutant protein. J Pharmacol Exp Ther. 2002;302(3):1286.

127. Takeda M, Khamdang S, Narikawa S, Kimura H, Kobayashi Y, Yamamoto T, et al. Human organic anion transporters and human organic cation transporters mediate renal antiviral transport. J Pharmacol Exp Ther. 2002;300(3):918.
