# Supplementary material for: Novel minimal physiologically-based model for the prediction of passive tubular reabsorption and renal excretion clearance
Source: Eur J Pharm Sci. 2016 Oct 30;94:59–71. doi: 10.1016/j.ejps.2016.03.018 (PMC5074076; doi:10.1016/j.ejps.2016.03.018)
Supplement: Supplementary file 4 — Supplementary Results [file mmc4.docx]

**Supplementary Results for the manuscript: “Novel minimal physiologically-based model for the prediction of passive tubular reabsorption and renal excretion clearance”**

Daniel Scotcher ^a^, Christopher Jones ^b^, Amin Rostami-Hodjegan ^a,c^ and Aleksandra Galetin ^a^

^a^ Centre for Applied Pharmacokinetic Research, Manchester Pharmacy School, University of Manchester, Manchester, United Kingdom

^b^ Oncology iMed, AstraZeneca, Alderley Park, United Kingdom

^c^ Simcyp Limited (a Certara Company), Sheffield, United Kingdom

Contents

[1. Figures 2](#_Toc427762794)

[2. Tables 7](#_Toc427762795)

[3. References 11](#_Toc427762796)

# 1. Figures

**
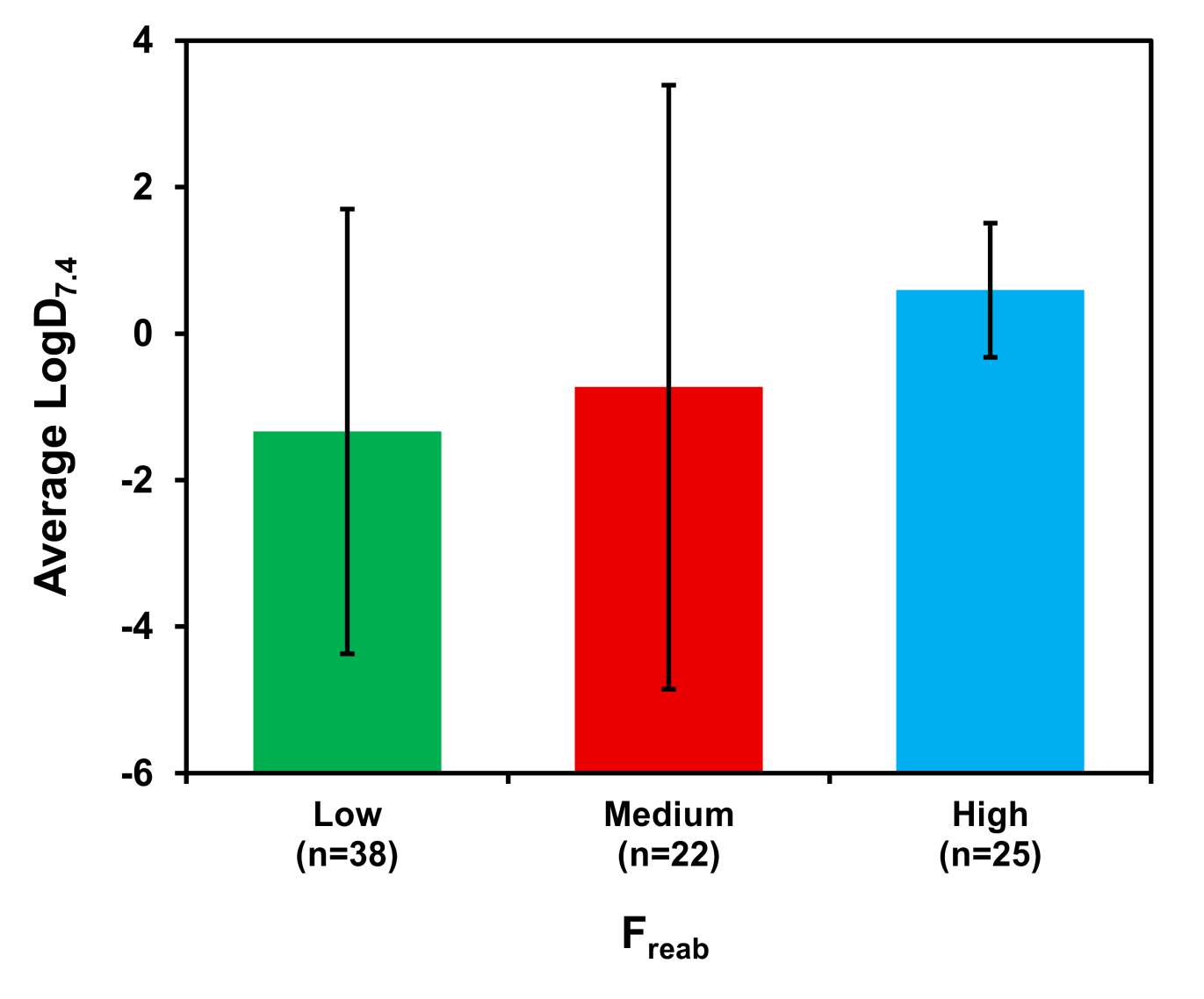
**

**Figure S4.1. Average LogD_oct­_ (pH 7.4) of drugs grouped by low (< 0.25), medium (0.25 – 0.75) and high (0.75 - 1) F_reab_.** The values for the classification system were arbitrarily chosen. Error bars indicate 1 standard deviation. Both measured and calculated LogD_oct_ (pH 7.4) data were included.

**
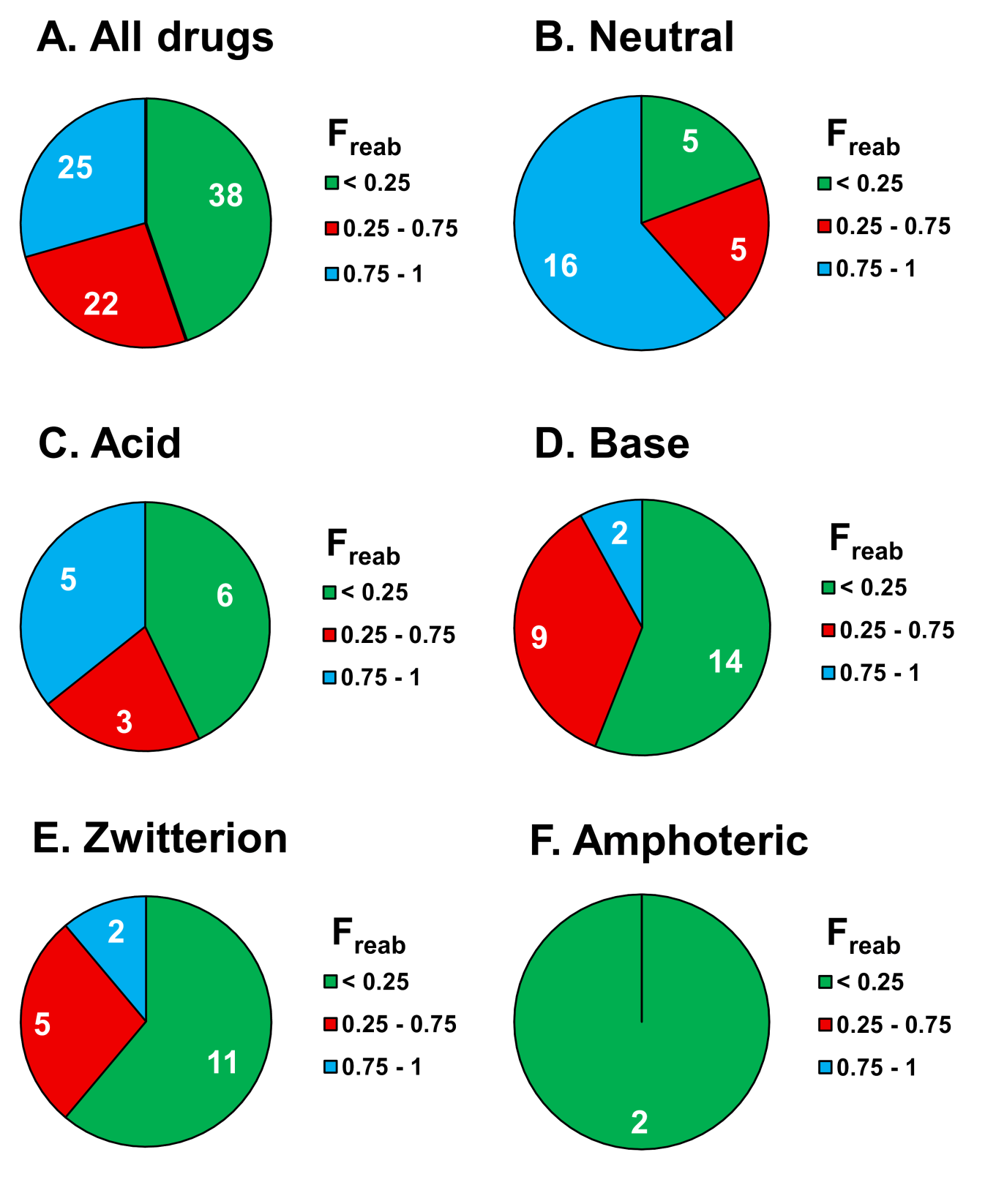
**

**Figure S4.2. Number of drugs classified as having high (0.75 - 1; blue), moderate (0.25 – 0.75; red) and low (<0.25; green) observed F_reab­­_ values.** The values for the classification system were arbitrarily chosen. (**A**) All drugs for which filtration of reabsorption was the dominant mechanism of CL_R_ (n=85). Drugs predominantly neutral (n=24) (**B**) or ionised as acids (n=14) (**C**), bases (n=25) (**D**), zwitterions (n=18) (**E**) or amphoteric (n=2) (**F**) at pH 6.5.


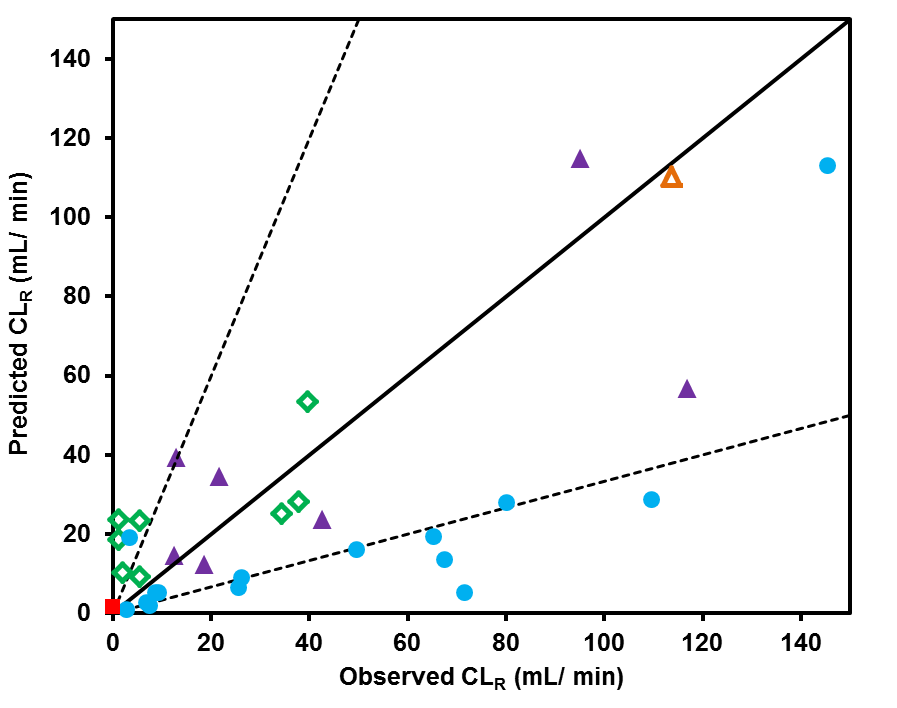


**Figure S4.3. Comparison of CL_R_ predicted using the minimal model of tubular reabsorption in combination with Caco-2 P_app_ from the pH7.4:7.4 configured assay, with observed CL_R_ (n=32 drugs).** Green open diamonds, blue solid circles, red solid squares, purple solid triangles and orange open triangles represent neutral, basic, acidic, zwitterion and amphoteric drugs respectively. Solid and dashed lines represent line of unity and 3-fold error respectively. P_app_ data was obtained from historical average values reported by AstraZeneca internal databases. Data were acquired using a variety of assay formats. AAFE was 3.09 for all drugs, with predicted CL_R_ within 3-fold of observed values for 18 drugs. Less than half of basic drugs were predicted within 3-fold of observed CL_R_.


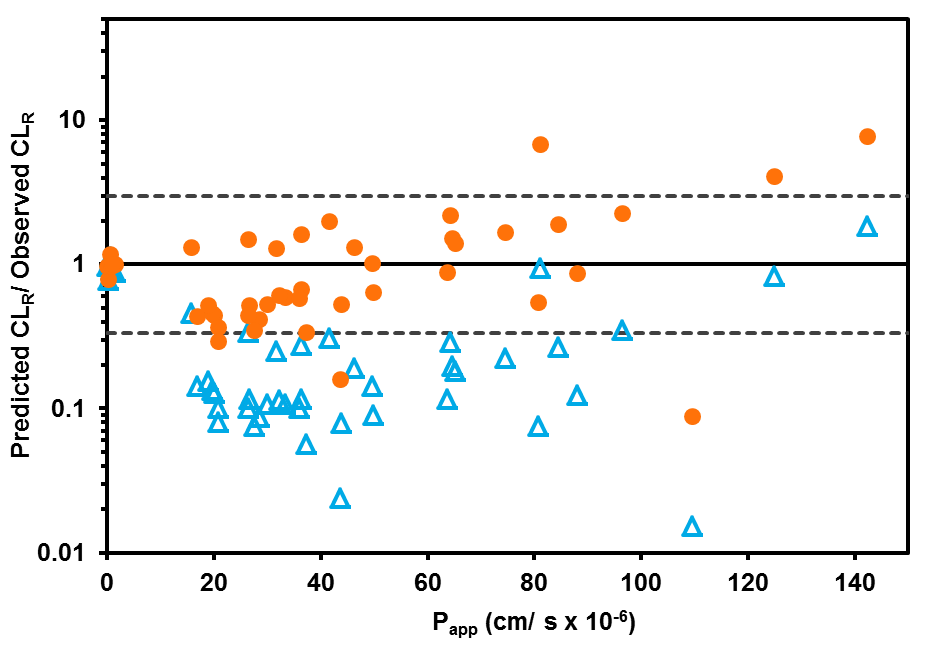


**Figure S4.4. Comparison of the predicted CL_R_/ observed CL_R_ ratio obtained using the reabsorption model with (orange circles) and without (blue open triangles) correction for the presence/ absence of microvilli.** Predicted CL_R_/ observed CL_R_ ratios of 1 (solid), 0.33 and 3 (dashed) are indicated by horizontal lines (i.e. unity and 3-fold error respectively). The model presented in the main text includes a correction factor (7.5-fold reduction), applied to the tubular surface area parameter (TSA_i_) of the loop of Henle, distal tubule and collecting duct compartments of the model. This correction factor accounts for the scarcity/ absence of microvilli in these tubular sections, in contrast to Caco-2 cells (and proximal tubule cells), which have a distinctive brush border.


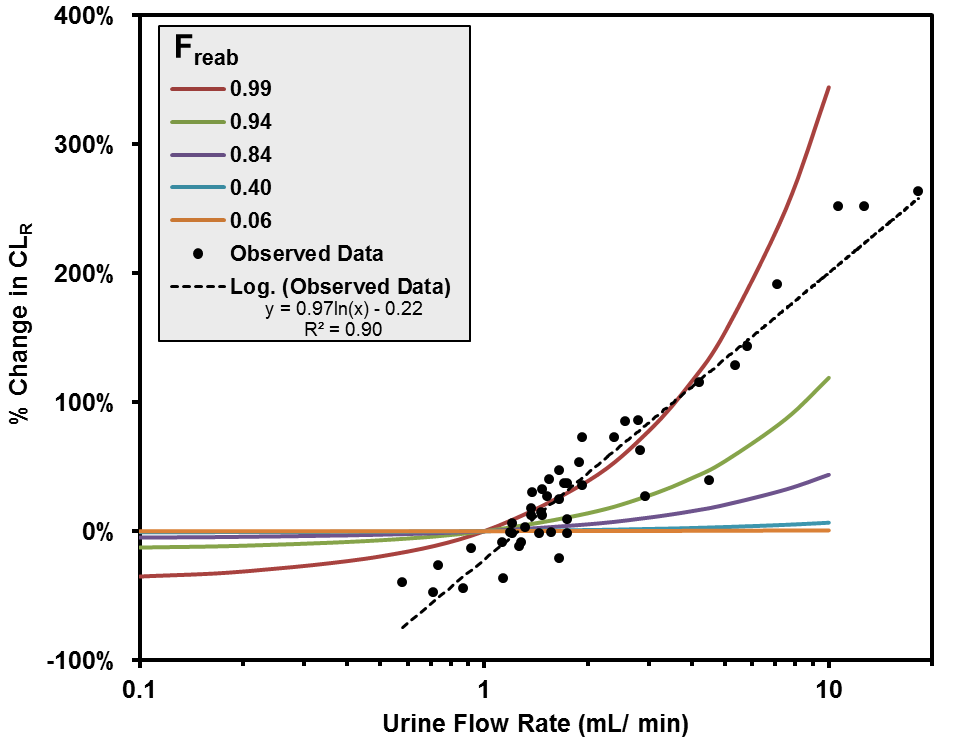


**Figure S4.5. Sensitivity analysis of urine flow rate on predicted CL_R_ for hypothetical drugs compared against observed data for theophylline.** Hypothetical drugs had Caco-2 P_app_ values ranging from 1 – 200 cm/ s × 10^-6^, resulting in predicted F_reab_ values ranging from 0.06 – 0.99 assuming a urine flow rate of 1 mL/ min (using Eq. 10 – 12 in the main text). Predicted F_reab_, and subsequently CL_R_, were re-calculated by changing the urine flow rate parameter to values ranging from 0.1 – 10 mL/ min (Eq. 9), and TFR_CD_ (Eq 11) after re-calculation of the mid-point tubular flow rate (see Supplementary Methods, Section 1 and Table S1.1). The % change in CL_R_ is calculated using CL_R_ predicted when urine flow rate = 1 mL/ min as baseline. Observed data for urine flow and matching theophylline CL_R_ were extracted from ([Tang-Liu et al., 1982](#_ENREF_5)), and % change was calculated, using as baseline the overall weighted mean theophylline CL_R_ obtained from the literature survey in the present study (Supplementary Results Table S2.1). The logarithm line of best fit for the observed data was plotted using MS Excel.

# 2. Tables

**Table S4.1. Specific studies or trials from the database of CL_R_ values, and reasons for exclusion**

| Study/ trial | Reason |
| --- | --- |
| Atenolol 10 mg IV infusion ([Fitzgerald et al., 1978](#_ENREF_1)) | Anomalous result. CL_R_ values reported in this trial were much higher than other studies for this drug, including other trials in the same publication. |
| Pilsicainide 50 mg Oral dose ([Tsuruoka et al., 2006](#_ENREF_6)) | Anomalous result. CL_R_ values reported in this study were much higher than other studies for this drug. |
| Verapamil ([Ho et al., 2000](#_ENREF_2)) | Much higher renal clearance reported compared with other available data ([Mikus et al., 1990](#_ENREF_3)). Other studies report a low fraction of dose excreted in urine ([Mooy et al., 1985](#_ENREF_4)). |

**Table S4.2. Overview of various CL_R­_ predictions for 45 drugs compared with observed CL_R_.**

| **Drug Name** | **Apparent F_reab_** | **Apparent F_reab_’** | **Reference drug for P_app_-F_reab_ calibration?** | **CL_R_ (mL/ min)** | | | | | |
| --- | --- | --- | --- | --- | --- | --- | --- | --- | --- |
|  |  |  |  | **Observed ^a^** | **Filtration only ^b^** | **Reabsorption model ^c^** | **P_app_-F_reab_ calibration ^d^** | **Proximal tubule only ^e^** | **No correction for microvilli ^f^** |
| Antipyrine | 0.989 | 0.997 | Yes | 1.22 | 106.80 | 4.98 | 1.98 | 16.92 | 1.00 |
| Aprindine | 0.785 | 0.791 | No | 1.28 | 5.96 | 1.66 | 3.10 | 2.48 | 0.32 |
| Atenolol | -0.247 | -0.249 | No | 145.33 | 116.52 | 114.79 | 116.52 | 115.21 | 112.16 |
| Betamethasone | 0.780 | 0.787 | No | 9.50 | 43.20 | 14.15 | 26.95 | 19.94 | 3.17 |
| Betaxolol | 0.081 | 0.081 | No | 49.64 | 54.00 | 21.98 | 40.78 | 28.64 | 6.28 |
| Caffeine | 0.987 | 0.995 | Yes | 1.06 | 79.80 | 7.20 | 5.78 | 17.71 | 0.99 |
| Chlorpheniramine | 0.260 | 0.262 | Yes | 26.20 | 35.40 | 10.86 | 20.63 | 15.69 | 2.28 |
| Chlorpropamide | 0.907 | 0.915 | No | 0.56 | 6.06 | 0.48 | 0.34 | 1.26 | 0.07 |
| Citalopram | -0.086 | -0.087 | No | 65.17 | 60.00 | 23.70 | 44.27 | 31.21 | 6.53 |
| Dapsone | 0.830 | 0.837 | No | 5.50 | 32.28 | 5.57 | 8.36 | 10.17 | 0.78 |
| Difloxacin | 0.939 | 0.947 | No | 4.54 | 74.25 | 7.56 | 6.96 | 17.56 | 1.01 |
| Doxepin | 0.638 | 0.643 | No | 9.75 | 26.94 | 6.54 | 11.80 | 10.35 | 1.12 |
| Fluconazole | 0.848 | 0.855 | No | 15.68 | 102.96 | 25.08 | 45.30 | 39.66 | 4.29 |
| Gabapentin | 0.183 | 0.185 | No | 95.05 | 116.40 | 111.97 | 116.37 | 113.03 | 105.43 |
| Grepafloxacin | 0.350 | 0.353 | No | 47.60 | 73.20 | 4.19 | 2.00 | 12.87 | 0.73 |
| Imipramine | 0.547 | 0.551 | No | 6.80 | 15.01 | 3.98 | 7.37 | 6.08 | 0.73 |
| Irbesartan | 0.623 | 0.628 | No | 2.37 | 6.29 | 1.25 | 2.06 | 2.15 | 0.19 |
| Isoxicam | 0.995 | 1.003 | No | 0.02 | 4.50 | 0.17 | 0.06 | 0.64 | 0.04 |
| Levetiracetam | 0.636 | 0.641 | No | 39.35 | 108.00 | 51.56 | 90.55 | 63.65 | 17.91 |
| Linezolid | 0.576 | 0.581 | Yes | 39.54 | 93.36 | 22.90 | 41.45 | 36.10 | 3.94 |
| Melagatran | -0.021 | -0.021 | No | 113.74 | 111.42 | 110.48 | 111.42 | 110.70 | 109.03 |
| Metoprolol | -0.050 | -0.050 | No | 109.58 | 104.40 | 47.50 | 85.07 | 59.58 | 15.52 |
| Metronidazole | 0.918 | 0.925 | No | 9.64 | 117.19 | 14.50 | 16.39 | 30.75 | 1.89 |
| Mexiletine | -0.252 | -0.254 | No | 71.61 | 57.20 | 11.42 | 18.84 | 19.61 | 1.71 |
| Moclobemide | 0.943 | 0.951 | No | 3.42 | 60.00 | 7.47 | 8.50 | 15.80 | 0.97 |
| Moxifloxacin | 0.372 | 0.375 | No | 42.65 | 67.89 | 22.10 | 42.10 | 31.22 | 4.92 |
| Oxprenolol | 0.443 | 0.447 | Yes | 9.35 | 16.80 | 4.93 | 9.32 | 7.25 | 0.99 |
| Oxytetracycline | 0.019 | 0.019 | Yes | 90.80 | 92.52 | 88.09 | 92.48 | 89.15 | 81.64 |
| Pefloxacin | 0.857 | 0.864 | No | 12.86 | 89.87 | 11.36 | 13.11 | 23.85 | 1.48 |
| Prednisolone | 0.099 | 0.100 | No | 34.30 | 38.07 | 15.70 | 29.03 | 20.37 | 4.55 |
| Prednisone | 0.299 | 0.301 | No | 37.87 | 54.00 | 12.79 | 22.88 | 20.46 | 2.14 |
| Probenecid | 0.955 | 0.963 | No | 0.50 | 11.22 | 0.95 | 0.72 | 2.41 | 0.13 |
| Propafenone | 0.258 | 0.260 | No | 7.37 | 9.93 | 3.26 | 6.21 | 4.59 | 0.73 |
| Propylthiouracil | 0.833 | 0.840 | No | 3.20 | 19.20 | 1.74 | 1.41 | 4.27 | 0.24 |
| Ribavirin | 0.084 | 0.085 | Yes | 109.94 | 120.00 | 109.85 | 119.81 | 112.26 | 95.83 |
| Ropivacaine | 0.547 | 0.552 | No | 2.73 | 6.03 | 1.65 | 3.08 | 2.49 | 0.31 |
| Sparfloxacin | 0.731 | 0.737 | Yes | 21.69 | 80.70 | 13.88 | 20.80 | 25.39 | 1.93 |
| Sulfamethoxazole | 0.894 | 0.901 | No | 4.52 | 42.42 | 8.94 | 15.18 | 15.00 | 1.38 |
| Tetracycline | 0.053 | 0.053 | No | 86.38 | 91.20 | 85.63 | 91.13 | 86.96 | 77.68 |
| Theophylline | 0.912 | 0.920 | Yes | 5.50 | 62.61 | 7.67 | 8.59 | 16.34 | 1.00 |
| Tocainide | 0.181 | 0.183 | Yes | 67.50 | 82.44 | 34.83 | 63.95 | 44.80 | 10.39 |
| Topiramate | 0.856 | 0.863 | No | 15.05 | 104.40 | 19.59 | 31.14 | 34.53 | 2.84 |
| Venlafaxine | 0.088 | 0.089 | No | 80.00 | 87.70 | 27.69 | 52.69 | 39.56 | 5.98 |
| Verapamil | -0.354 | -0.357 | No | 25.60 | 18.90 | 7.45 | 13.93 | 9.82 | 2.05 |
| Voriconazole | 0.969 | 0.977 | Yes | 1.57 | 50.40 | 3.51 | 2.09 | 9.79 | 0.54 |

^a^ Observed CL_R_ is overall weighted mean of values obtained from a literature survey; ^b^ CL_R,filt_ calculated using Eq. 7 in main text; ^c^ CL_R_ predicted using tubular reabsorption model, as per Eq. 10 – 12 of main text; ^d^ CL_R_ predicted using the tubular reabsorption model after calibration of P_app_ data using Eq. 14; ^e^ CL_R_ predicted using a model with only one tubular compartment representing proximal tubule (main contributor to reabsorption predicted by the model); ^f^ No correction was made for surface area attributable to presence/ absence of microvilli when calculating CL_R,int,reab,i_.

# 3. References

Fitzgerald, J., Ruffin, R., Smedstad, K., Roberts, R., McAinsh, J., 1978. Studies on the pharmacokinetics and pharmacodynamics of atenolol in man. Eur J Clin Pharmacol 13 (2), 81.

Ho, P., Ghose, K., Saville, D., Wanwimolruk, S., 2000. Effect of grapefruit juice on pharmacokinetics and pharmacodynamics of verapamil enantiomers in healthy volunteers. Eur J Clin Pharmacol 56 (9-10), 693.

Mikus, G., Eichelbaum, M., Fischer, C., Gumulka, S., Klotz, U., Kroemer, H., 1990. Interaction of verapamil and cimetidine: stereochemical aspects of drug metabolism, drug disposition and drug action. J Pharmacol Exp Ther 253 (3), 1042.

Mooy, J., Schols, M., Muytjens, A., Rahn, K., 1985. Pharmacokinetics of verapamil in patients with renal failure. Eur J Clin Pharmacol 28 (4), 405.

Tang-Liu, D.D.-S., Tozer, T.N., Riegelman, S., 1982. Urine flow-dependence of theophylline renal clearance in man. J Pharmacokinet Biopharm 10 (4), 351-364.

Tsuruoka, S., Ioka, T., Wakaumi, M., Sakamoto, K., Ookami, H., Fujimura, A., 2006. Severe arrhythmia as a result of the interaction of cetirizine and pilsicainide in a patient with renal insufficiency: first case presentation showing competition for excretion via renal multidrug resistance protein 1 and organic cation transporter 2. Clin Pharmacol Ther 79 (4), 389.
